# DNA-encoded library versus RNA-encoded library selection enables design of an oncogenic noncoding RNA inhibitor

**DOI:** 10.1073/pnas.2114971119

**Published:** 2022-02-02

**Authors:** Raphael I. Benhamou, Blessy M. Suresh, Yuquan Tong, Wesley G. Cochrane, Valerie Cavett, Simon Vezina-Dawod, Daniel Abegg, Jessica L. Childs-Disney, Alexander Adibekian, Brian M. Paegel, Matthew D. Disney

**Affiliations:** ^a^Department of Chemistry, The Scripps Research Institute, Jupiter, FL 33458;; ^b^Department of Chemistry and Pharmaceutical Sciences, University of California, Irvine, CA 92617

**Keywords:** RNA, drug design, nucleic acids, RNA folding

## Abstract

Nature evolves molecular interaction networks through persistent perturbation and selection, in stark contrast to drug discovery, which evaluates candidates one at a time by screening. Here, nature’s highly parallel ligand-target search paradigm is recapitulated in a screen of a DNA-encoded library (DEL; 73,728 ligands) against a library of RNA structures (4,096 targets). In total, the screen evaluated ∼300 million interactions and identified numerous bona fide ligand–RNA three-dimensional fold target pairs. One of the discovered ligands bound a 5′GAG/3′CCC internal loop that is present in primary microRNA-27a (pri-miR-27a), the oncogenic precursor of microRNA-27a. The DEL-derived pri-miR-27a ligand was cell active, potently and selectively inhibiting pri-miR-27a processing to reprogram gene expression and halt an otherwise invasive phenotype in triple-negative breast cancer cells. By exploiting evolutionary principles at the earliest stages of drug discovery, it is possible to identify high-affinity and selective target–ligand interactions and predict engagements in cells that short circuit disease pathways in preclinical disease models.

## Significance

Drug discovery generally investigates one target at a time, in sharp contrast to living organisms, which mold ligands and targets by evolution of highly complex molecular interaction networks. We recapitulate this modality of discovery by encoding drug structures in DNA, allowing the entire DNA-encoded library to interact with thousands of RNA fold targets, and then decoding both drug and target by sequencing. This information serves as a filter to identify human RNAs aberrantly produced in cancer that are also binding partners of the discovered ligand, leading to a precision medicine candidate that selectively ablates an oncogenic noncoding RNA, reversing a disease-associated phenotype in cells.

Traditional drug discovery entails screening a single or a few targets for binding to a library of small molecules (1). The dominant high-throughput screening paradigm is responsible for generating countless new lead molecules. These screening data sets and the accompanying compound library files guide medicinal chemistry by way of off-target effects gleaned from prior screens and deep physicochemical and pharmacokinetic analyses that all library members undergo, ultimately focusing the library on “drug-like” chemical matter (2, 3). High-throughput screens are limited to the same “drug-like” chemical matter (2, 3), leading to the classification of a target as “druggable” or “undruggable.” However, the screened chemical matter, developed for “druggable” protein and enzyme targets, is not appropriate for all potential target classes.

Genetic encoding of targets and small molecules has greatly increased both the scope of library property space and the information content obtained from such screens (4–8). DNA-encoded library (DEL) technology permits the design and synthesis of highly diverse (∼10^6^) collections of small molecules, each bound to a structure-encoding DNA tag (4, 5, 9–12). Affinity selection and sequencing of the bound species’ DNA tags affords potent ligands and rich structure–activity data (13, 14). Complementarily, RNA sequencing technology has become a powerful tool for identifying selective RNA ligands (15–19). For example, two-dimensional combinatorial screening (2DCS) presents a library of RNA three-dimensional ( 3D) folds to a small molecule microarray; bound RNAs are excised from the microarray and sequenced (20, 21). Thousands of compounds can be screened for binding to thousands of RNA targets, but each compound must be individually synthesized, purified, and assayed for RNA library binding.

Herein, we integrate 2DCS with solid-phase DEL in a massively parallel screening pipeline to probe affinity landscapes between RNA folds and small molecules. In brief, fluorescence-activated cell sorting (FACS) identified DEL beads that specifically bind to RNA folds. Selective small-molecule RNA ligands were isolated in a FACS analysis of DEL beads (73,728 members) that were incubated with two differentially labeled RNAs: 1) a library displaying the randomized region in a 3 × 3 nucleotide internal loop pattern (3 × 3 ILL; 4,096 members) and 2) a fully base-paired RNA counter screen target. Sequencing and informatic analysis revealed affinity landscapes and candidate target miRNAs for each discovered ligand. A compound with nanomolar affinity for oncogenic primary microRNA-27a (pri-miR-27a) was discovered and shown to inhibit miRNA biogenesis and rescue a migratory phenotype in triple-negative breast cancer (TNBC) cells.

## Results

### Design of the DNA-Encoded Small-Molecule Library and the RNA 3D Fold Library.

The DEL was synthesized and validated following published protocols (14, 22). Each bead contained sites for compound synthesis and sites for enzymatic ligation of the encoding oligonucleotide. During encoded combinatorial synthesis, each chemical building block was assigned a unique short DNA sequence that was ligated to the beads after coupling the building block. The PCR primer binding sites were installed prior to initiating and after completing encoded combinatorial synthesis, yielding DEL beads that display PCR-amplifiable DNA encoding tags (*SI Appendix*, Scheme S1, Tables S1 and S2).

**Fig. 1.**
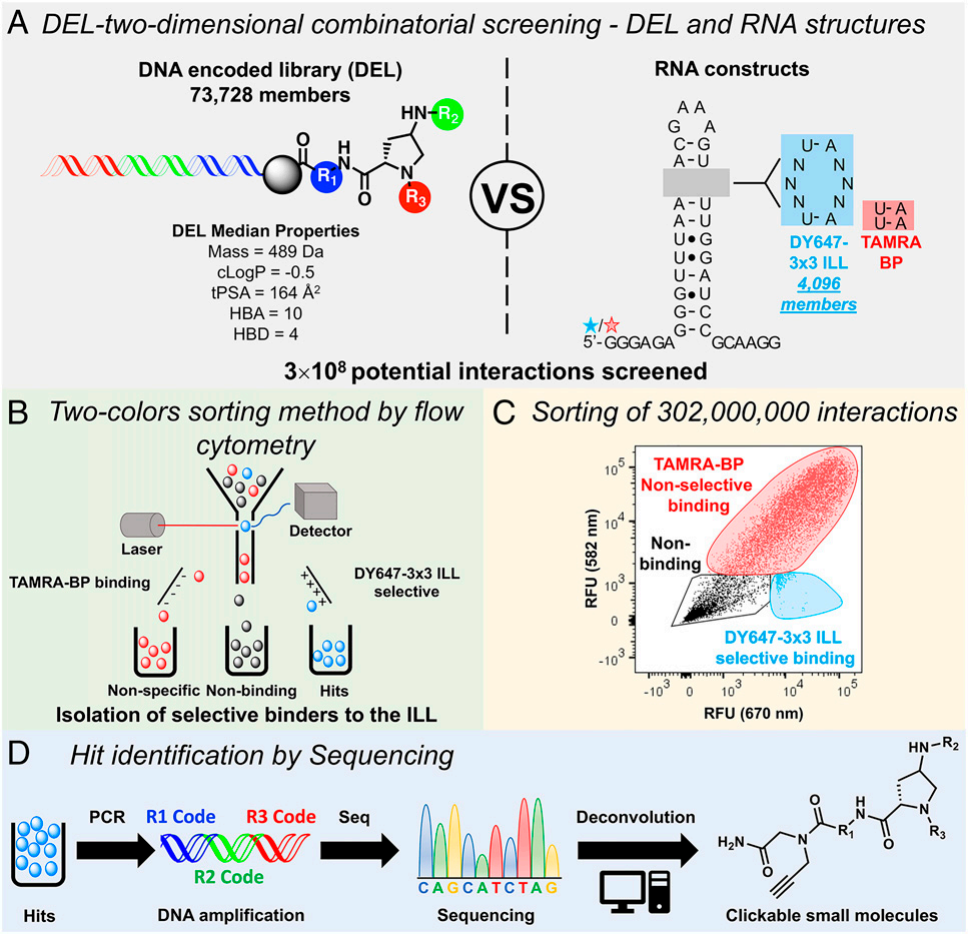
Identification of RNA-binding ligands using a DEL and 2DCS. (*A*) Schematic of the solid-phase DEL (73,728 unique compounds) screened for binding to a DY647-labeled RNA library with a randomized region in a 3 × 3 nucleotide internal loop pattern, DY647-3 × 3 ILL (4,096 unique RNA 3D folds). The screen was completed in the presence of an orthogonally labeled base-paired control RNA, TAMRA-BP. The DEL was designed to contain molecules with RNA-binding or “antibacterial-like” properties. (*B*) Two-color FACS identified beads that specifically bound DY647-3 × 3 ILL. (*C*) The FACS histogram collection gate (cyan) highlights beads that selectively bound DY647-3 × 3 ILL. (*D*) Identification of the hit structures that bound selectively to DY647-3 × 3 ILL library by sequencing their DNA tags.

The physical properties of RNA-binding antibacterial natural products, some of which exhibit unconventional physiochemical properties, inspired the DEL design and building block pool composition. Each library member contains 1 of 96 amino acids (R1), a central Fmoc-Pro(N_3_)-OH hub, then 1 of 192 carboxylic acids. The carboxylic acid was installed after either Fmoc deprotection and acylation of the pendant secondary amine (R2) or Staudinger reduction of the azide and acylation of the pendant primary amine (R3). The remaining pendant Fmoc groups were removed, and azides reduced to yield a 73,728-member DEL of diverse primary or secondary amine-containing compounds. A stoichiometric mixture of two Fmoc-Pro(N_3_)-OH diastereomers was used for the central hub coupling, and this position was not encoded. Amino acid and carboxylic acid building blocks were selected that display diverse tertiary amine and hydroxyl functionalities. Thus, DEL members tended to be higher in molecular weight, more hydrophilic, and more functionalized with hydrogen bond donors and acceptors compared to chemical matter contained in previous solid-phase DEL screening studies (*SI Appendix*, Table S3 and Figs. S1 and S2; Fig. 1*A*) (23, 24).

We screened the DEL using a previously described RNA fold library (20, 25). The RNA structure library displays a randomized region in a 3 × 3 nucleotide internal loop pattern (4,096 members) fluorescently labeled with DY647 (DY647-3 × 3 ILL) (Fig. 1*A*). The randomized region is embedded into a unimolecular hairpin with single-stranded 5′ and 3′ tails that contain primer binding sites for PCR amplification and sequencing (25). As a counter screen to eliminate molecules that bind RNA nonselectively, we used a tetramethylrhodamine-labeled RNA in which the 3 × 3 nucleotide randomized region was replaced with base-paired nucleotides (TAMRA-BP; Fig. 1*A*).

### FACS Analysis of Solid-Phase DEL RNA Structure Library Binding Assay.

An aliquot of the DEL (∼750,000 beads) was incubated with DY647-3 × 3 ILL and TAMRA-BP, bovine serum albumin, and transfer RNA (tRNA). Two-color FACS analysis isolated DEL beads that bound specifically to DY647-3 × 3 ILL by sorting beads with high DY647 fluorescence (λ_ex_/λ_em_ = 652/673 nm) and low TAMRA fluorescence (λ_ex_/λ_em_ = 555/580 nm) (Fig. 1 *B* and *C*; *SI Appendix*, Fig. S3). The analysis yielded 102 DY647 sort events (0.01% hit rate) and 60 TAMRA sort events (0.008% hit rate); most RNA-binding beads bound both DY647- and TAMRA-labeled RNA [i.e., nonselectively (*SI Appendix*, Fig. S4)].

**Fig. 2.**
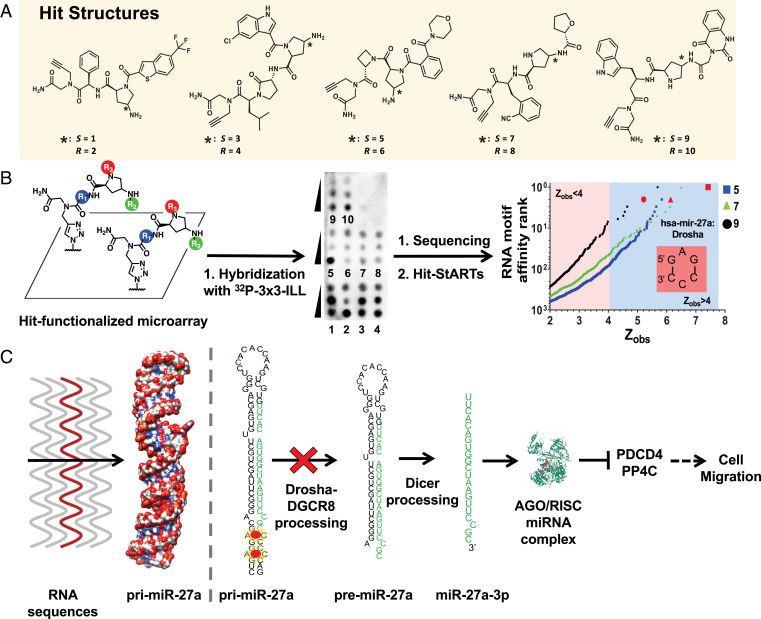
2DCS selection to generate transcriptome-wide structure–activity relationships across the human miRnome. (*A*) Several structural families were discovered after the 2DCS selection process, which was completed under highly stringent conditions. (*B*) Schematics present the construction of 2DCS microarrays, representative microarray image of the 2DCS selection of hit compounds with ^32^P-labeled 3 × 3 ILL, and the affinity landscapes for **5**, **7**, and **9**. All three compounds bound the 5′GAG/3′CCC internal loop present in pri-miR-27a’s Drosha processing site. (*C*) Transcriptome-wide mining analysis identified pri-miR-27a as a druggable RNA target, containing the 5′GAG/3′CCC internal loop in its Drosha processing site. Mature miR-27 regulates the expression of PDCD4 and PP4C, the repression of which contributes to migration and oncogenicity.

DEL bead hits were pooled, amplified, sequenced, and decoded to identify hit structures for structure–activity analysis and for prioritization for synthesis and subsequent validation. Sequence pattern matching identified unique DY647 and TAMRA hit beads (23), and hit structures were clustered according to Tanimoto chemical similarity (0.8) (26). Hits were then ranked in each cluster according to their replicate (*k*) class; that is, the number of times the same compound was observed as a hit on different FACS-sorted beads (Fig. 1*D*; *SI Appendix*, Fig. S5) (22, 27). Each library member was screened as a mixture of two Pro(N_3_) diastereomers, thus each hit was synthesized as optically pure compounds in diastereomer pairs (e.g., compounds **1** and **2**). Hits **1** to **10** are the highest *k* class representatives of five of the top six most populous clusters of DY647 hits. Hits **5**/**6** (*k* = 6) represent the largest hit cluster (16 additional k > 1 hit structures). Hits **3**/**4** (*k* = 9), **7**/**8** (*k* = 5), and **9**/**10** (*k* = 8) were also high *k* class hits and were the representative of small (one- or two-member) clusters (Fig. 2*A*).

Several common features of hit structures appeared to be important for selectively binding the RNA structure library, guiding our selection of hits for scaled synthesis and further exploration. The top three represented cycle 1 building blocks were the Freidinger’s lactam of hits **3**/**4**, D-threonine, and the azetidine of hits **5**/**6** (40, 21, and 14 hits of the 181 DY647 hits, respectively). The top represented cycle 2 building blocks included the chloroindole of hits **3**/**4**, the morpholine-carbonyl benzoic acid of hits **5**/**6**, the trifluoromethyl benzothiophene of hits **1**/**2**, the tetrahydrofuran of hits **7**/**8**, and the dioxo-dihydroquinazoline of hits **9**/**10**. The dioxo-dihydroquinazoline representation resulted from a single high *k* class hit structure, indicating that this structure was important for RNA binding and likely required the specific cycle 1 *L*-β-homotryptophan context. Overrepresentation in cycle 2 is notable as this is the highest diversity cycle (192 acids). Acylation at the C_ɑ_ N via Fmoc deprotection was preferred to acylation at the C_ɣ_ N via azide reduction for ILL-selective ligands (137 versus 44 DY647 hit beads, respectively). Thus, hit structures collectively explored overrepresented building blocks and acylation position in the context of high *k* class hit structures.

Owing to the DEL design, the physicochemical properties of compounds **1** to **10** are similar to known RNA-binding small molecules. LogD and LogP tend to be lower, and the number of hydrogen bond donors and acceptors tend to be higher compared to DELs that were designed and synthesized to sample canonical Lipinski–Veber drug-like chemical space (*SI Appendix*, Fig. S2 and Table S4) (2, 3, 23). Despite these similarities, however, **1** to **10** are chemically dissimilar to known RNA-binding compounds. The average Tanimoto score for each compound against all small molecules housed in Inforna, an expansive database of known ligand–RNA interactions, is 0.3 ± 0.01 (*SI Appendix*, Fig. S5). Therefore, the DEL screen identified chemotypes that bind RNA 3D folds.

### Identification of the RNA 3D Folds that Bind to SPDEL Compounds via 2DCS.

The interaction landscape for the 10 representative DEL compounds emerging from the FACS screen were then defined by 2DCS (20). In 2DCS, small molecules are site specifically conjugated to a microarray surface and then probed for binding to an RNA library under highly stringent conditions (Fig. 2*B*; *SI Appendix*, Fig. S6). That is, the array is coincubated with a radioactively labeled RNA library and an excess of competitor oligonucleotides that mimic regions common to all library members (28), as compared both to the total number of moles of compound delivered to the array surface and the RNA library. To enable site-specific immobilization of compounds **1** to **10**, a C-terminal alkyne handle was installed for copper(I)-catalyzed azide alkyne cycloaddition (CuAAC) click (28) coupling to azide-displaying array surfaces. Installation of the alkyne was accomplished by coupling of bromoacetic acid onto rink amide resin followed by nucleophilic displacement with propargylamine prior to the synthesis of the SPDEL compounds (Fig. 2*A*).

All 10 compounds bound to the 3 × 3 ILL under the highly stringent conditions, almost all dose dependently (Fig. 2*B*). The RNAs bound to each small molecule were harvested, reverse transcribed into DNA, and identified by RNA sequencing. The resulting data set was statistically analyzed by using high-throughput structure–activity relationships through sequencing (Hit-StARTS) (29), in which the relative frequency of each selected RNA is compared to the frequency of that RNA in the starting library (pooled population comparison) (29). The statistical significance of the enrichment of selected RNAs is quantified by Z_obs_, which correlates with affinity and selectivity (29). Here, we employed a cutoff of Z_obs_ > 4, which was empirically determined from measured affinities via evaluation of compound **9** binding with potential RNA motifs extracted from the 2DCS (Fig. 2*B*; *SI Appendix*, Figs. S7 and S8). Notably we demonstrated the direct correlation between Z_obs_ and binding affinities (*SI Appendix*, Fig. S8). This cutoff afforded, on average, 60 ± 40 (range: 6 to 155) preferred, or privileged, RNA 3D folds for each compound. Compound **6** bound to largest number of RNA targets (*n* = 155), while compound **9** bound the fewest (*n* = 6) (*SI Appendix*, Fig. S8). Interestingly, each compound bound a set of RNAs that is unique, ranging from 2 (compounds **4** and **9**) to 41 motifs (compound **3**). Altogether, **1** to **10** bound 212 unique 3D folds, of which 31 motifs (14%) previously had no known ligand binding partner. Of the 212 motifs identified, none is present in human tRNAs, and only 24 are present in human ribosomal RNA (30).

Sequence logos (LOGOS), which graphically depicts sequence preferences as “bits” of information, were generated for each DEL-derived ligand (*SI Appendix*, Fig. S9) (31). Difference logos (DiffLOGO) (32) revealed that some diastereomers of the same compound (**1** and **2**; **3** and **4**, etc.; odd numbers are the S diastereomer, even numbers are the R diastereomer) are partial to similar sequences while others are very different (*SI Appendix*, Fig. S9). For example, **9** prefers internal loops with A’s while **10** selects pyrimidine-rich loops. In contrast, the four loops with the highest Z_obs_ scores for **1** and **2** are very similar, and **3** and **4** both selected pyrimidine-rich loops, particularly cytosines (*SI Appendix*, Fig. S8). Interestingly, three of the R diastereomers, **6**, **8**, and **10**, have similar LOGOS with strong preferences for U in positions 1 and 2 and C in position 3. Furthermore, these trends are not observed in their R diastereomers (which generally have less sequence preference throughout all positions of the randomized region), suggesting that the proline stereochemistry influences its preferred RNA 3D folds (*SI Appendix*, Fig. S9). Thus, our data suggest the stereochemistry of a ligand influences its molecular recognition fingerprints for RNA targets.

### SAR on the Human Transcriptome.

Inforna provides unprecedented ability to define structure-activity relationship (SAR) on the human transcriptome. A compound’s selectivity is a function of the number and type of RNA folds that it binds, the frequency of the folds in the transcriptome, and the expression level of the RNAs in which they reside. We therefore mined the human miRnome in miRBase (33) for the 212 RNA motifs with Z_obs_ > 4.0 that bound **1** to **10**. Of these motifs, 123 (58%) were present in human miRnome, and 8 were in Drosha or Dicer processing sites of a disease-associated miRNA (*SI Appendix*, Fig. S6). Interestingly, three compounds, **5**, **7**, and **9**, were predicted to bind to the 5′GAG/3′CCC internal loop present in the Drosha processing site of pri-miR-27a, albeit with different affinities based on their Z_obs_ scores; miR-27a is an oncogenic miRNA with established roles in breast and prostate cancers (34–36). In addition to the 5′GAG/3′CCC loop present in pri-miR-27a’s Drosha site, a second copy of the loop is also present nearby (*SI Appendix*, Fig. S10). Binding of its Drosha site with a ligand might inhibit miR-27a biogenesis and ablate oncogenic phenotypes in cancer cell lines (Fig. 2*C*). Although the 5′GAG/3′CCC motif of interest is present in nine human miRNAs, it is only present in processing sites of miR-27a and miR-409 (Drosha) and, as such, would only be predicted to affect the processing of these targets (*SI Appendix*, Fig. S10). Of the two, only miR-27a is significantly expressed across various cell lines/types, as indicated by the number of reads per million from the sequencing data available in the miRBase 22.1 (October 2018) (33); miR-27a is expressed at ∼220-fold–greater levels on average than miR-409 (for miR-27a: 85,900 reads per million, 3,554,414 total reads analyzed over 159 experiments; for miR-409a: 391 reads per million, 19,718 reads analyzed over 128 experiments) (33). We therefore further pursued studies on the small molecule targeting of pri-miR-27a with **5**, **7**, and **9**.

First, we measured the in vitro binding affinity of these compounds toward the putative preferred binding motif, 5′GAG/3′CCC, by microscale thermophoresis (*SI Appendix*, Fig. S11). Compound **9** demonstrated the highest binding affinity with K_d_ of 90 ± 20 nM, followed by **5** and **7** with K_d_s of 700 ± 100 and 530 ± 90 nM, respectively. To study the potential importance of the closing base pairs, we mined the affinity landscape for related internal loops that did not bind the small molecules. None of the molecules were predicted to bind an AC internal loop in which the 5′ closing base pair was mutated from GC to CG (indicated in bold), that is 5′**C**AG/3′**G**CC. Interestingly, this loop is adjacent to the 5′GAG/3′CCC that binds **5**, **7**, and **9** in pri-miR-27a (Fig. 2*C*). Saturable binding was not observed for any of the three compounds for the mutated loop (K_d_ > 50 µM) or for a fully paired RNA (*SI Appendix*, Fig. S11). Moreover, **10**, a stereoisomer of **9**, binds to none of the three RNAs, indicating the crucial role of **9**’s stereochemistry for binding (*SI Appendix*, Fig. S11).

To measure the affinity of **9** for the 5′GAG/3′CCC loop in the context of pri-miR-27a, we used a competitive binding assay with a constant concentration of compound (100 nM) and the Cy5-labeled model of pri-miR-27a’s Drosha site and varying concentrations of unlabeled miR-27a precursor as reported by miRBase (33). That is, the miR-27a precursor competes with the Cy5-labeled model of pri-miR-27a’s Drosha site for binding **9**. In this assay, **9** bound to pri-miR-27a with a K_d_ of 40 ± 30 nM; binding was abolished when the two 5′GAG/3′CCC loops were mutated to base pairs (*SI Appendix*, Fig. S12). Thus, the compounds identified by using DEL-2DCS can bind selectively to biologically relevant RNA targets identified by Inforna.

### Compound 9 Inhibits the Cellular Processing of pri-miR-27a.

**Fig. 3.**
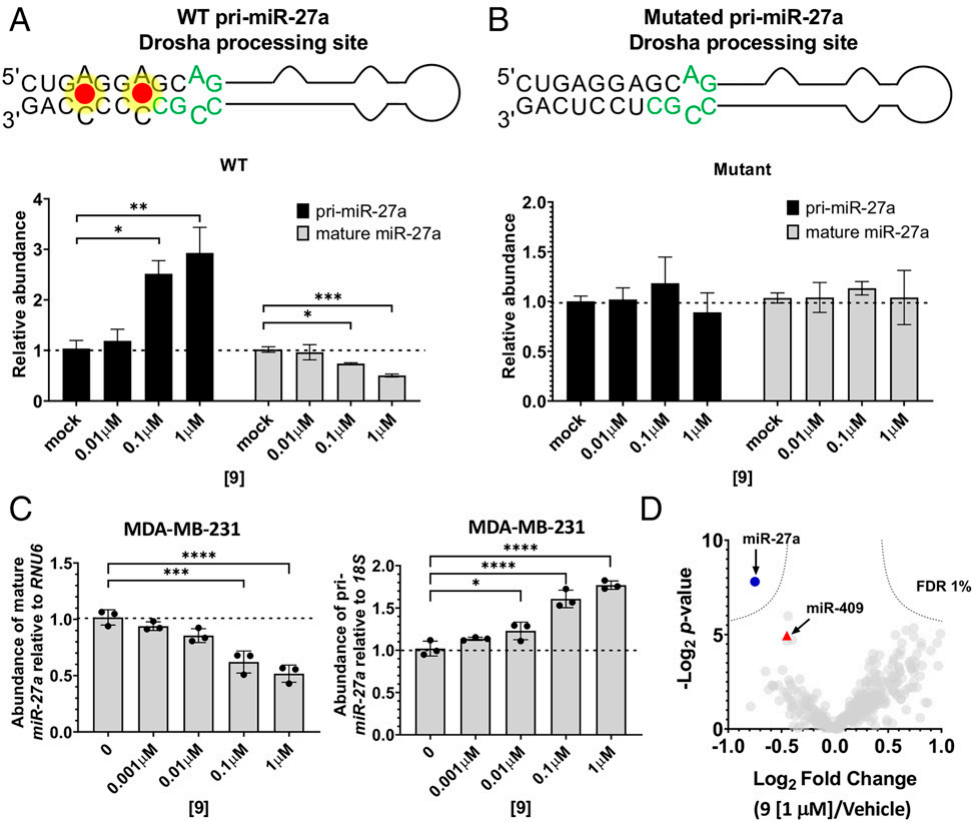
Compound 9 inhibits the biogenesis of pri-miR-27a in MCF-10a (forced expression) and MDA-MB-231 (endogenous) cells. (*A*) The secondary structure of WT pri-miR-27a contains tandem **9** binding sites. WT primary and mature miR-27a expression levels in transfected MCF-10a cells changed significantly and in a dose-dependent fashion upon treatment with **9** (RT-qPCR, *n* = 3). MCF-10a cells do not otherwise appreciably express miR-27a (C_t_ > 31). “Mock” indicates mock-transfected, vehicle-treated cells. (*B*) Binding sites for **9** were eliminated in a synthetic base-paired mutant pri-miR-27a. Base-paired mutant primary and mature miR-27a expression levels in transfected MCF-10a cells did not change in response to treatment with **9** (RT-1PCR, *n* = 3). “Mock” indicates mock-transfected, vehicle-treated cells. (*C*) Endogenous pri-miR-27a biogenesis and mature miR-27a accumulation in MDA-MB-231 TNBC cells increased and decreased, respectively, in a dose-dependent fashion (RT-qPCR, *n* = 3). (*D*) miRnome analysis of MDA-MB-231 cells treated with **9** (1 µM) revealed highly selective attenuation of mature miR-27a levels (FDR). The error bars are reported as SD for all panels. *P < 0.05; **P < 0.01; ***P < 0.001; ****P < 0.0001, as determined by a one-way ANOVA relative to “0” (untreated cells).

To gauge the specificity of **9** for pri-miR-27a, we first studied inhibition of biogenesis in a cellular model of healthy breast epithelium, MCF-10a, in which miR-27a expression is not detectable (C_t_ > 31). MCF-10a cells were transfected with a plasmid encoding wild-type (WT) pri-miR-27a or mutant in which the two 5′GAG/3′CCC internal loops at and nearby the Drosha processing site were mutated to base pairs. The mutant is properly processed by Dicer and Drosha to produce the mature miR-27a; however, by nature of the mutation in the small-molecule binding site, it should not be affected by the small molecule. Indeed, in the absence of compound treatment, both primary miRNA transcripts were processed similarly, generating mature miR-27a, as determined by RT-qPCR. Notably, expression of both WT pri-miR-27a and the mutant conferred migratory characteristics to MCF-10a cells, the same phenotype observed in TNBC cells that is caused by miR-27a overexpression (*SI Appendix*, Fig. S13) (34). Importantly, **9** only inhibited the biogenesis of WT pri-miR-27a, as evidenced by reduction of mature miR-27a levels and an increase of pri-miR-27a levels, as determined by RT-qPCR (Fig. 3*A*). In contrast, **9** did not inhibit biogenesis of the mutated pri-miR-27a in MCF-10A (Fig. 3*B*) or rescue the migratory phenotype induced by its forced expression (*SI Appendix*, Fig. S13). Collectively, these studies in MCF-10a show that **9** specifically binds the 5′GAG/3′CCC internal loop present in pri-miR-27a’s Drosha processing site.

Given these favorable results in MCF-10a cells, we studied whether **9** could inhibit pri-miR-27a biogenesis (i.e., reduce mature miR-27a abundance) in MDA-MB-231 TNBC cells, in which its overexpression is tied to oncogenesis (34). In agreement with its putative mode of action, **9** dose dependently reduced mature miR-27a levels with a half maximal inhibitory concentration (IC_50_) of ∼1 μM (Fig. 3*C*) and increased pri-miR-27a levels, as determined by RT-qPCR (Fig. 3*C*). Furthermore, in agreement with their in vitro binding affinities, **9** inhibited miR-27a to a greater extent than **5** and **7**, while **9**’s diastereomer, **10**, was inactive (*SI Appendix*, Figs. S14–S16). Notably, **9** inhibited miR-27a biogenesis dose dependently as assessed by its effect on pri- and mature miR-27a levels in three other cancer cell lines in which miR-27a is aberrantly expressed: MCF-7 (breast cells) (37), LNCaP (prostate adenocarcinoma) (35), and HeLa (cervical cancer) (38) (*SI Appendix*, Fig. S17). Significant inhibition of miR-27a processing was observed at a dose as low as of 100 nM in all three cancer cell lines (*SI Appendix*, Fig. S17).

### Specificity of 9 for the Functional Inhibition of miR-27a.

Interestingly, pri-miR-27a is part of a larger pri-miRNA cluster, transcribed as a single transcript along with pri-miR-23a and pri-miR-24, which may act individually or cooperatively to regulate gene expression (39). The processing of each transcript appears to occur independently, as varying expression levels have been observed in different cell types, and miR-27a can be down-regulated independently of the other two (40). For example, previous studies showed that forced expression of the cluster in HEK293T cells increased levels of miR-27a and miR-24-2 but not miR-23a, indicating a block in the processing of pri-miR-23a (39); this block was not observed in HeLa or P19 (mouse teratocarcinoma) cells (41). As expected from these previous studies, **9** had no effect on the levels of mature miR-24-2 or miR-23a upon treatment of MDA-MB-231 cells (*SI Appendix*, Fig. S18).

A broader view of **9**’s selectivity across the miRnome was afforded by profiling the levels of all miRNAs detectable in MDA-MB-231 cells, represented as a volcano plot, a logarithmic plot of fold change as a function of statistical significance (Fig. 3*D*). The only statistically significant effect observed upon treatment with 1 μM of **9** (false discovery rate [FDR] < 1%; *P* < 0.01) was the down-regulation of miR-27a. Notably, the levels of miR-409, which has the same Drosha processing site as miR-27a, were not affected, likely due to its significantly lower expression levels in MDA-MB-231 cells (128-fold less abundant) and hence lower cellular occupancy by **9**. It has been previously shown that target expression level influences bioactivity (16). That is, the highly expressed RNA is occupied by the small molecule to a greater extent than an RNA with the same binding site of lower abundance; hence a greater effect on the former’s function is observed. Furthermore, the abundance of other oncogenic miRNAs, such as miR-96, miR-155, and miR-21 known to be also correlated with TNBC, were not affected (Fig. 3*D*) (42–44).

### Effect of 9 on the Proteome.

As our miRnome-wide studies indicated that **9** is specific for inhibition of miR-27a biogenesis, we investigated whether these effects translated to the proteome. Notably, miR-27a has been implicated in the regulation of various pathways, including oncogenesis (45, 46). In particular, Zinc Finger and BTB Domain Containing 10 (ZBTB10, also known as RINZF) is known to be down-regulated by miR-27a in breast cancer cells, particularly MDA-MB-231 cells (39, 46). Additionally, TargetScan (47) predicts that Protein Phosphatase 4 Catalytic (PP4C) and Programmed Cell Death 4 (PDCD4) are direct targets of miR-27a, both of which have been directly correlated with migratory functions of breast cancer cell lines (48, 49).

**Fig. 4.**
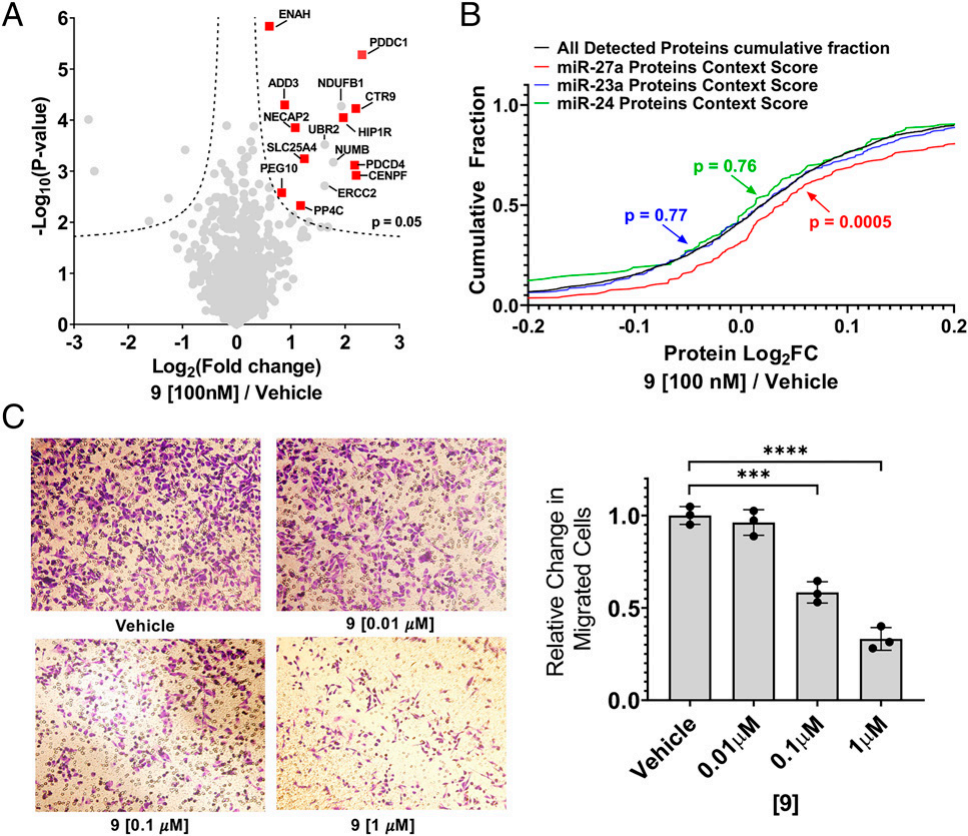
Effect of **9** proteome wide and on the migratory nature of MDA-MB-231 cells. (*A*) Proteomic analysis of MDA-MB-231 cells treated with **9** (liquid chromatography with tandem mass spectrometry (LC-MS/MS), fold change relative to vehicle treatment) confirmed the increased abundance of proteins under regulation of miR-27a expression (red squares; *n* = 3). The dotted lines indicate FDR = 5% and S0 of 0.1, where S0 is the minimum fold change required for significance; adjusted *P* = 0.05. (*B*) Cumulative distribution plots of the fold change of proteins in **9**- versus vehicle-treated samples indicated a significant up-regulation of only miR-27a targets (red), while no significant change was observed with miR-23a targets (green) and miR-24 (blue), relative to the cumulative distribution of all proteins (black). TargetScanHuman version 7.2 was used to predict downstream protein targets whose messenger RNAs contain conserved sites for miR-27a-3p (*n* = 1,421), miR-23a-3p (*n* = 247), and miR-24-3p (*n* = 1,342). Expression levels of miR-27a-5p, miR-23a-5p, and miR-24-5p are very low in MDA-MB-231 cells (C_t_ ∼28 to 32). Of these predicted targets, 220 downstream targets of miR-27 were detectable in proteomics analysis (∼15%), while 247 (∼18%) and 122 (∼16%) were detectable for miR-23a and miR-24, respectively. Note miR-27a is transcribed as part of a cluster with miR-23a and miR-24. *P* values between distributions were calculated using a two-tailed Kolmogorov–Smirnov test. (*C*) Representative micrographs capture the reduction in migrated MDA-MB-231 cells as a function of [**9**]. Image analysis indicated dose-dependent attenuation of the migration phenotype normalized to vehicle-treated cells. The error bars are reported as SD; *n* = 3 replicates from two independent experiments; three fields of view analyzed per sample. ***P < 0.001; ****P < 0.0001, as determined by a one-way ANOVA relative to “vehicle.”

To investigate the overall effect of **9** on the proteome including miR-27a’s direct targets, MDA-MB-231 cells were treated with 100 nM of the compound and subjected to global proteomics analysis as previously described (50). Of the 3,127 detectable proteins, only 15 were significantly up-regulated and 5 significantly down-regulated (FDR < 5%; Fig. 4*A*). A total of 11 of the 15 up-regulated proteins are putative direct targets of miR-27a [as per TargetScan (47)] including ENAH Actin Regulator (ENAH), Adducin 3 (ADD3), NECAP Endocytosis Associated 2 (NECAP2), Paternally Expressed 10 (PEG10), PP4C, and PDCD4. Only one of these up-regulated proteins is directly targeted by miR-23a (Solute Carrier Family 25 Member 4; SLC25A4) or miR-24 (*SI Appendix*, Fig. S19); however, this protein is also directly regulated by miR-27a (*SI Appendix*, Fig. S19). We confirmed the up-regulation of PP4C, PDCD4, and ZBTB10 by Western blotting (*SI Appendix*, Fig. S20), which mirrored the dose-dependent reduction of mature miR-27a levels.

We next analyzed the changes in expression levels across the entire proteome to quantify the effects of **9** on protein levels encoded by messenger RNAs that are regulated by miR-27a, miR-24, or miR-23a, as determined by the corresponding context score reported by TargetScan (51). This analysis showed that the changes observed proteome-wide were correlated with the targets of miR-27a but not with the targets of miR-24 or miR-23a (Fig. 4*B*; *SI Appendix*, Fig. S19).

As the proteomics data indicated a specific effect on miR-27a–regulated proteins, the effect of **9** on phenotype, namely the migratory mature of MDA-MB-231 cells, was assessed. A dose-dependent reduction in the number of migrated cells was observed upon treatment with **9**, with statistically significant phenotype reduction using as little as 100 nM small molecule (Fig. 4 *C* and *D*; *SI Appendix*, Fig. S21). In conjunction with studies in MCF-10a cells forced to express WT or mutant pri-miR-27a (Fig. 3), these studies collectively demonstrate rescue of the miR-27a–mediated oncogenic phenotype by **9**.

## Discussion

Herein, we have described and validated an approach to cross reference combinatorially synthesized chemical matter with RNA 3D fold space. Deep sequencing of both the DNA-encoded ligands and the RNA targets proved transformative in the same manner that sequencing has enabling technologies such as ribosome profiling (52, 53) and cell atlasing (54), which provide high-definition summaries of cellular metabolism and fate (55). Our approach integrates two orthogonal and yet highly complementary library analyses, deploying evolutionary principles (56–59) at the earliest stage of probe discovery. Screening hits are identified with confidence by pairing redundant hit isolation in FACS (27) with in vitro selection–based RNA motif ranking by sequence abundance (20).

This study represents a major capability for DEL screening in the way of RNA library interrogation. Nucleic acid binding protein and nucleic acid targets have long been eschewed due to problems of DEL-nonspecific binding to other nucleic acids. As of this writing, there is only one record of targeting a nucleic acid during DEL selection, and it involves the highly structured DNA G-quartets (60). Here, we simultaneously decouple the DNA encoding tag and encoded library member through substoichiometric functionalization of each DEL bead (13) and leverage dual-channel simultaneous counter screening to triage nonselective RNA ligands by way of the base-paired control, a strategy that was also highly effective in identifying selective IgG ligands from plasma (22). Radiometric and high-throughput 2DCS-based RNA simultaneously affords an orthogonal mode of validation and an added layer of statistical hypothesis testing in the form of RNA target abundance analysis of the selection.

Collectively, this approach substantiates a compelling platform for evaluating novel and designed chemical matter for its suitability to target RNA, a major ongoing initiative in the field of RNA-targeted drug discovery. Using this DEL-enabled approach, it will be possible to design and synthesize arbitrarily diverse libraries for screening and potentially map whole chemical spaces and their proclivity to bind RNA 3D folds in addition to mapping specific small-molecule structures to transcriptomic binding sites of interest as in this study. With additional analyses of divergent libraries, it may become possible to predict the principal components that confer RNA-binding properties.

Our screening results demonstrate that DEL-2DCS may be poised to deliver a trove of structures with unexpected propensity to bind nucleic acids functionally in cells. Many RNA-binding molecules have tended to be flat, interacting with nucleic acids via π-stacking interactions, although there are many examples of groove binders as well. Here, we have shown that these properties are not necessarily dominant considerations for RNA binding. Although plenty of DEL hits contained heavily conjugated and electron rich π-systems evocative of nucleic acid stains, the most prevalent building block, the Freidinger’s lactam, lacks such features. Furthermore, we found that small-molecule diastereomer pairs exhibited markedly different RNA target binding fingerprints. Structural studies will be required to define the precise mode of molecular recognition; however, our data suggest that perhaps molecular recognition of RNA targets can be achieved by using ligands that resemble those that target proteins.

This combinatorial approach between DEL and RNA targets yielded bioactive ligands that inhibited processing of miR-27a, an important oncogenic miRNA associated with various cancers. Notably, the lead molecule bound miR-27a with nanomolar affinity, targeting an internal loop pocket present twice within the Drosha processing site. Compound **9** significantly decreased miR-27a expression in four different cancer cell lines at nanomolar concentrations with high selectivity across the miRnome. The inhibition of its biogenesis selectively induced the derepression of associated proteins and the diminution of the migratory phenotype associated with breast cancer disease.

Taken altogether, this study provides an unprecedented approach for the identification of RNA 3D fold ligands. By exploiting evolutionary principles at the earliest stages of drug discovery, target–ligand interactions were identified and the engagement of cellular RNAs by these ligands was predicted, ultimately inducing short circuiting of an oncogenic pathway. The human transcriptome sequence is a well-known reservoir of new targets. Sequence-based design of structure-specific ligands coupled with an evolutionary search for molecular recognition events could provide a streamlined platform to define bioactive interactions and deliver chemical probes that affect RNA function and biology.

## Materials and Methods

All materials and experimental methods can be found in the *SI Appendix*. They include synthetic methods, which describe the synthesis of the solid-phase DEL and quality control thereof, bar coding, and the synthesis, purification, and characterization of hit compounds. With regard to the screening of the DEL for binding the RNA library, methods are provided for analysis by FACS, deconvolution of the DEL binders, and chemoinformatic analysis. Methods describing in vitro studies include preparation of microarrays for 2DCS selection, the 2DCS selection itself, and binding affinity measurements. The *SI Appendix* also provides methods for cellular studies, including those for cell culture and compound treatment. Other cellular methods include those for RT-qPCR analysis to measure miRNA abundance and Western blotting to assess the effect of compound treatment on PDCD4 and ZBTB10 as well as β-actin (used for normalization). A description of the methods used for migration assays to assess phenotype with and without compound treatment, and global proteomics studies to assess the selectivity of compound treatment on the proteome are also provided.

## Supplementary Material

Supplementary File

## Data Availability

All data are included in the article and/or *SI Appendix*.
