# Supplementary material for: DNA-encoded library versus RNA-encoded library selection enables design of an oncogenic noncoding RNA inhibitor
Source: Proc Natl Acad Sci U S A. 2022 Feb 2;119(6):e2114971119. doi: 10.1073/pnas.2114971119 (PMC8833215; doi:10.1073/pnas.2114971119)
Supplement: Supplementary File [file pnas.2114971119.sapp.pdf]

Supporting Information:

DNA-encoded library-versus-RNA-encoded library selection enables design of an oncogenic non-coding RNA inhibitor

Raphael I. Benhamou[1,†], Blessy M. Suresh[1,†] , Yuquan Tong[1], Wesley G. Cochrane[2], Valerie Cavett[2], Simon Vezina-Dawod[1], Daniel Abegg[1], Jessica L Childs-Disney[1], Alexander Adibekian[1], Brian M. Paegel[1,2,*], and Matthew D. Disney[1,*]

[1]Department of Chemistry, The Scripps Research Institute, Jupiter, Florida 33458, USA

[2] Department of Chemistry and Pharmaceutical Sciences, University of California, Irvine, Irvine, CA 92617, USA

* Correspondence should be addressed to M.D. Disney, email: disney@scripps.edu or to B.M. Paegel, email: bpaegel@uci.edu

†These authors contributed equally.

SUPPLEMENTARY SCHEMES, TABLES & FIGURES

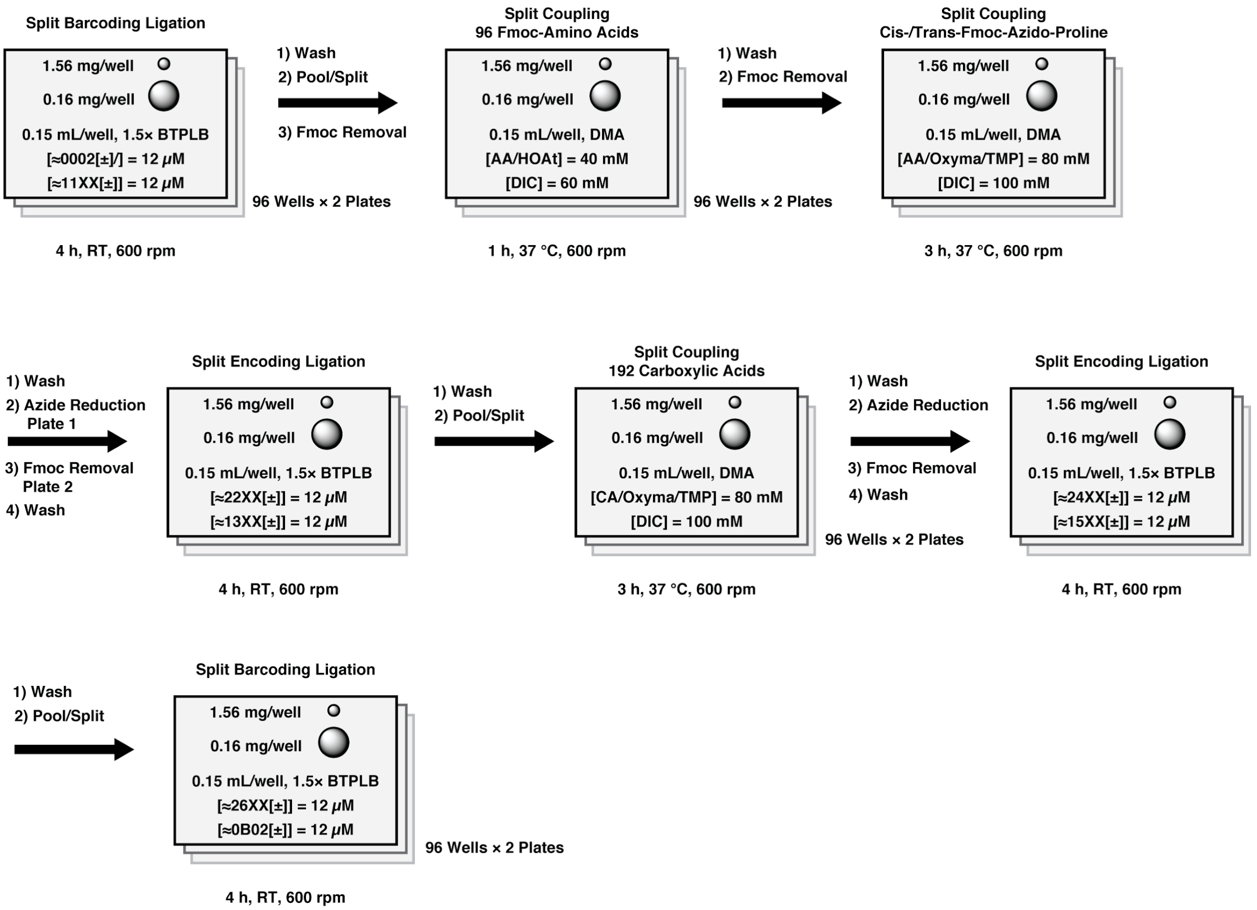

Scheme S1. Scheme for the DEL combinatorial synthesis.

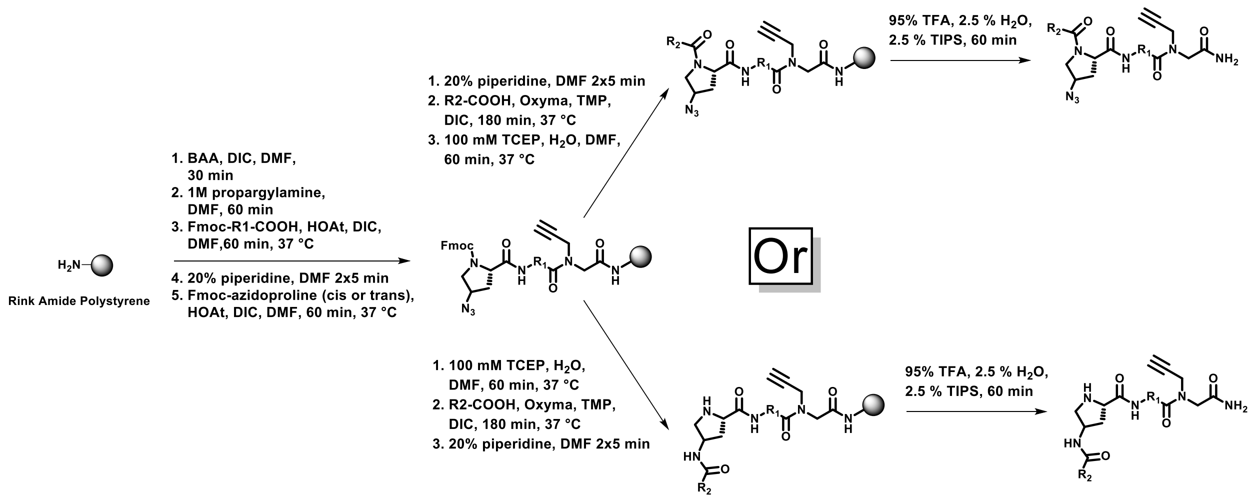

Scheme S2. Parallel synthesis of the hit compounds on solid support.

Table S1. Sequences of oligonucleotides overhangs, and PCR primers used to construct the DEL.

Overhang ID	Overhang [+]	Overhang [-]
≈X1XX[+]	/5Phos/ATGG	/5Phos/TGA
≈X2XX[+]	/5Phos/TCA	/5Phos/AAC
≈X3XX[+]	/5Phos/GTT	/5Phos/TAG
≈X4XX[+]	/5Phos/CTA	/5Phos/GAA
≈X5XX[+]	/5Phos/TTC	/5Phos/GCG
≈X6XX[+]	/5Phos/CGC	/5Phos/AGGC
PCR Primer ID	PCR Primer [+]	PCR Primer [-]
≈0002	/5Phos/GCCGCCGCCTTCGTCCTTCT CAGCGAC	5'/5Phos/CCATGTCGCTGAGAAGG ACGAAGGCGGCGGCGG
≈0B02	/5Phos/GCCTCCCAAACNNNNNNNNG TTTGCCCGCCAGTTGTTGTGCCAC	GTTTGGG
Phos indicates phosphate; N indicates any nucleotide (A, G, C, or T)		

**Table S2.** DNA tags used to decode DEL building blocks.					
Code 1 ID	Code 1 [+]	Code 1 [-]	Code 2 ID	Code 2[+]	Code 2 [-]
≈1X17	ACAAGAAA	TTTCTTGT	≈2X01	CCTCCTAA	TTAGGAGG
≈1X18	ACAAGGCT	AGCCTTGT	≈2X02	AACCTCAA	TTGAGGTT
≈1X19	ACAGGGTA	TACCCTGT	≈2X03	AATCCCAT	ATGGGATT
≈1X20	ACGAAAGA	TCTTTCGT	≈2X04	AACCCTAC	GTAGGGTT
≈1X21	ACGAGATT	AATCTCGT	≈2X05	ATCCTCTC	GAGAGGAT
≈1X22	ACGAGGGC	GCCCTCGT	≈2X06	CATTTCAA	TTGAAATG
≈1X23	ACGGAATC	GATTCCGT	≈2X07	CGCCTTCA	TGAAGGCG
≈1X24	ACGGGAAG	CTTCCCGT	≈2X08	CGTTCCTG	CAGGAACG
≈1X25	AGAAGACC	GGTCTTCT	≈2X09	TTCTTCAT	ATGAAGAA
≈1X26	AGGAAGGG	CCCTTCCT	≈2X10	TCCTCTTA	TAAGAGGA
≈1X27	AGGGAAAT	ATTTCCCT	≈2X11	AACCTTCG	CGAAGGTT
≈1X28	ATAAGGGA	TCCCTTAT	≈2X12	AACTCCCG	CGGGAGTT
≈1X29	ATAGAGCC	GGCTCTAT	≈2X13	AACTCTTT	AAAGAGTT
≈1X30	CAAAGACT	AGTCTTTG	≈2X14	AATCCTCA	TGAGGATT
≈1X31	CAAAGGAC	GTCCTTTG	≈2X15	AATCTCCC	GGGAGATT
≈1X32	CAAGAAGA	TCTTCTTG	≈2X16	AATCTTGT	ACAAGATT
≈1X33	CAAGAGTC	GACTCTTG	≈2X17	AATTCCGA	TCGGAATT
≈1X34	CAGAAGGA	TCCTTCTG	≈2X18	ACCCTCCT	AGGAGGGT
≈1X35	CAGAGAAA	TTTCTCTG	≈2X19	ACCCTTGA	TCAAGGGT
≈1X36	CAGGGACG	CGTCCCTG	≈2X20	ACCTCCAA	TTGGAGGT
≈1X37	CCGAAACT	AGTTTCGG	≈2X21	ACCTCTCC	GGAGAGGT
≈1X38	CCGAGGAG	CTCCTCGG	≈2X22	ACCTTCGC	GCGAAGGT
≈1X39	CCGGAGGG	CCCTCCGG	≈2X23	ACTCCCGC	GCGGGAGT
≈1X40	CGAGAACC	GGTTCTCG	≈2X24	ACTCCTTT	AAAGGAGT
≈1X41	CGAGGAGG	CCTCCTCG	≈2X37	CACCTCGC	GCGAGGTG
≈1X42	CGAGGGCA	TGCCCTCG	≈2X38	CACCTTAT	ATAAGGTG
≈1X43	CGGGAATA	TATTCCCG	≈2X39	CACTCCAT	ATGGAGTG
≈1X44	CGGGAGCT	AGCTCCCG	≈2X40	CATCCCTA	TAGGGATG
≈1X45	CTGAAGCC	GGCTTCAG	≈2X41	CCCTCCGG	CCGGAGGG
≈1X46	CTGGAAAC	GTTTCCAG	≈2X42	CCCTTCTA	TAGAAGGG
≈1X47	GAGAGGGT	ACCCTCTC	≈2X43	CCCTTTCG	CGAAAGGG
≈1X48	GAGGAACA	TGTTCCTC	≈2X44	CCTCTCAT	ATGAGAGG
≈1X49	GAGGGAAT	ATTCCCTC	≈2X45	CCTTTCCC	GGGAAAGG
≈1X50	GCAAAGGG	CCCTTTGC	≈2X46	CGCTCCCA	TGGGAGCG
≈1X51	GCAGAGAA	TTCTCTGC	≈2X47	CGTCCCAC	GTGGGACG
≈1X52	GCAGGACC	GGTCCTGC	≈2X48	CGTCCTGG	CCAGGACG
≈1X53	GCGGAAGT	ACTTCCGC	≈2X49	CGTCTCCG	CGGAGACG
≈1X54	GCGGGATA	TATCCCGC	≈2X50	CTCCCTCG	CGAGGGAG

≈1X55	GGAAGAGA	TCTCTTCC	≈2X51	CTTCCCGT	ACGGGAAG
≈1X56	GGAGAGGT	ACCTCTCC	≈2X52	GACTCCGC	GCGGAGTC
≈1X57	GGAGGATT	AATCCTCC	≈2X53	GCCCTCGG	CCGAGGGC
			≈2X54	GCCCTTCC	GGAAGGGC
			≈2X55	GCCTCCTT	AAGGAGGC
			≈2X56	GCTCCCTG	CAGGGAGC
			≈2X57	GGCCCTAA	TTAGGGCC
			≈2X58	GGCTCTCG	CGAGAGCC
			≈2X59	GGCTTCCC	GGGAAGCC
			≈2X60	GGTCCCGA	TCGGGACC
			≈2X61	GGTTTCGG	CCGAAACC
			≈2X62	GTTTCCCG	CGGGAAAC
			≈2X63	TACCTCTT	AAGAGGTA
			≈2X64	TACTTCGA	TCGAAGTA
			≈2X65	TCCCTCAC	GTGAGGGA
			≈2X66	TCCTTTGT	ACAAAGGA
			≈2X67	TCTCCTCC	GGAGGAGA
			≈2X68	TCTTCCTC	GAGGAAGA
			≈2X69	TGTCCCTT	AAGGGACA
			≈2X70	TGTCTTCT	AGAAGACA
			≈2X71	TGTTCTAA	TTAGAACA
			≈2X72	TTCCCTAT	ATAGGGAA

Table S3. Summary of the quality control (QC) for the synthesis of the DEL		
Compound ID	Compound Structure	Mass Observed (Expected [M+H] with Linker)
1	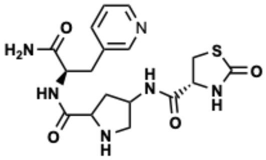	1396.8 (1396.6)
2	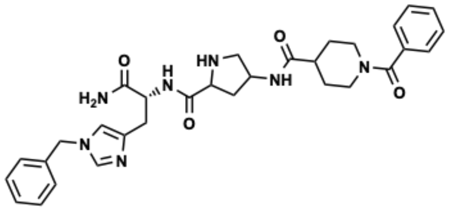	1562.1 (1561.7)
3	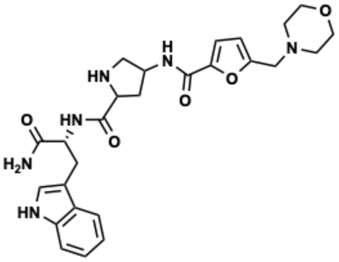	1498.9 (1498.7)
4	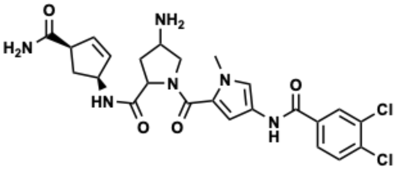	1522.9 (1522.6)
5	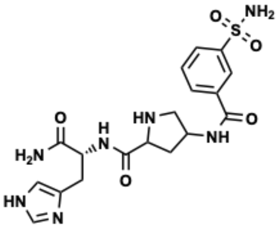	1440.0 (1439.6)
6	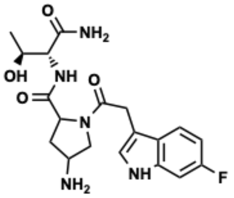	1396.0 (1395.6)
7	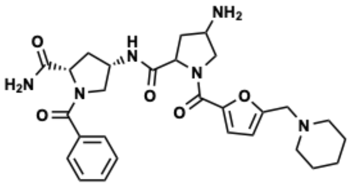	1527.0 (1526.7)

8	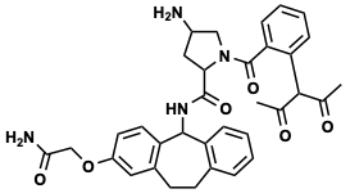	1384.9 (1384.6*)
9	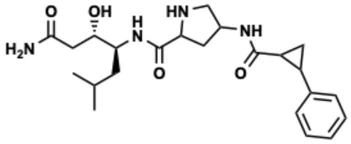	1421.0 (1420.7)
10	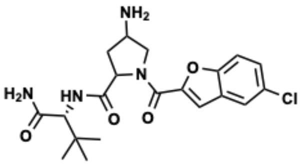	1410.8 (1410.6)
11	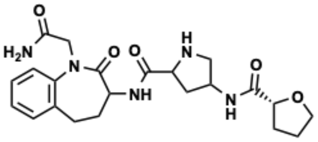	1433.8 (1433.6)
12	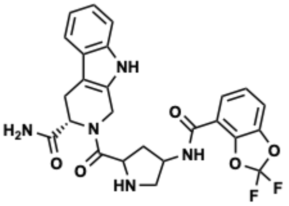	1501.9 (1501.6)
13	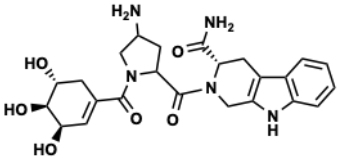	1473.9 (1473.6)
14	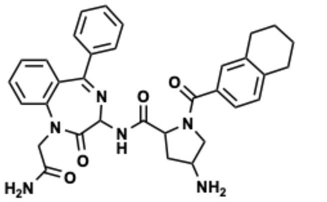	1568.9 (1568.7)
15	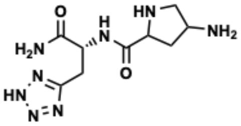	1258.8 (1258.6)
16	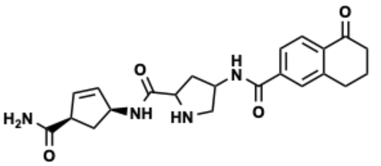	1400.8 (1400.6)

17	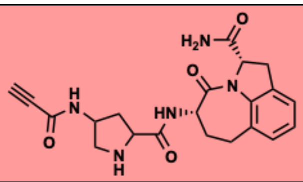	1417.8 (1449.6)
18	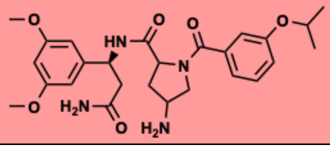	1518.9 (1488.7)
19	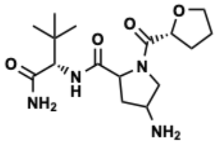	1330.8 (1330.6)
20	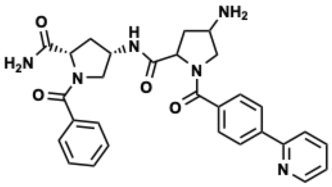	1516.7 (1516.7)

Red = Unmatched

Table S4: Physicochemical properties of small molecule libraries.					
Library	*MW (Da)*	*LogP*	*TPSA (Å)*	*H Acceptors*	*H Donors*
DrugBank	*345±198*	*2.0±3.5*	*92±114*	*5.0±6*	*2.0±4*
SPDEL – Starting Library	493±80	-0.54±2	163±55	10.0±2	4.0±1
SPDEL - Hits	*566±51*	*-0.87±2*	*168±20*	*6.4±1*	*3.8±1*
Inforna compounds	*457±203*	*0.16±5*	*156±118*	*8.6±6*	*5.2±5*

Table S5. Sequences of primers used RT-qPCR.

Gene	Forward Primer (5'→3')	Reverse Primer (5'→3')
RNU6	ACACGCAAATTCGTGAAGCGTTC	Universal: GAATCGAGCACCAGTTACGC
miR-27a	TTCACAGTGGCTAAGTTCCGC	Universal: GAATCGAGCACCAGTTACGC
miR-23a	ATCACATTGCCAGGGATTTCC	Universal: GAATCGAGCACCAGTTACGC
miR-24	TGGCTCAGTTCAGCAGGAACAG	Universal: GAATCGAGCACCAGTTACGC
pri-miR-27a	GAGCAGGGCTTAGCTGCTT	GTGAACACGACTTGGTGTGG
18S	GTAACCCGTTGAACCCCATT	CCATCCAATCGGTAGTAGCG
FBXW7	ACTGGAAAGTGACTCTGGGA	TACTGGGGCTAGGCAAACAA
GAPDH	AAGGTGAAGGTCGGAGTCAA	AATGAAGGGGTCATTGATGG

**Table S6.** Sequences of primers and template for transcription (5’→3’)	
Template for WT *pri-miR-27a*	CTGAGGAGCAGGGCTTAGCTGCTTGTGAGCAGGGTCCACACCA AGTCGTGTTCACAGTGGCTAAGTTCCGCCCCCCAG
Template for mutated *pri-miR-27a*	CTGAGGAGCAGGGCTTAGCTGCTTGTGAGCAGGGTCCACACCA AGTCGTGTTCACAGTGGCTAAGTTCCGCTCCTCAG
Forward primer	TAATACGACTCACTATAGAGAGAGGCCCCGAAGCCTGTGCCTG GCCTGAGGAGCAGGGCT
Reverse primer for WT	GGCAAGGCCAGAGGAGGTGAGGGCCTGGGGGGCGGAACT
Reverse primer for mutated	GGCAAGGCCAGAGGAGGTGAGGGCCTGAGGAGCGGAACT

* Red indicates mutated bases

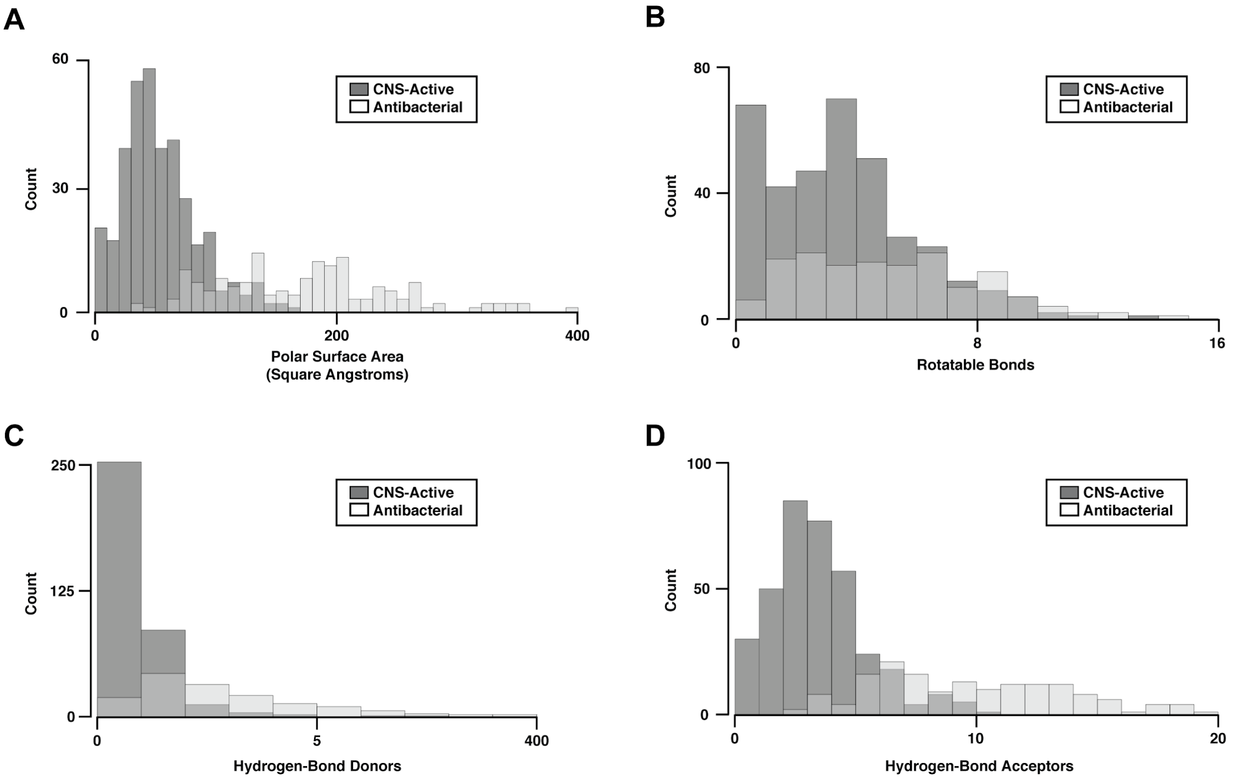

Figure S1. Figure S1. Molecular properties of all approved CNS-Active drugs (dark gray) and antibacterials (light gray). (A) polar surface area, (B) rotatable bonds, (C) hydrogen-bond donors, and (D) hydrogen-bond acceptors.

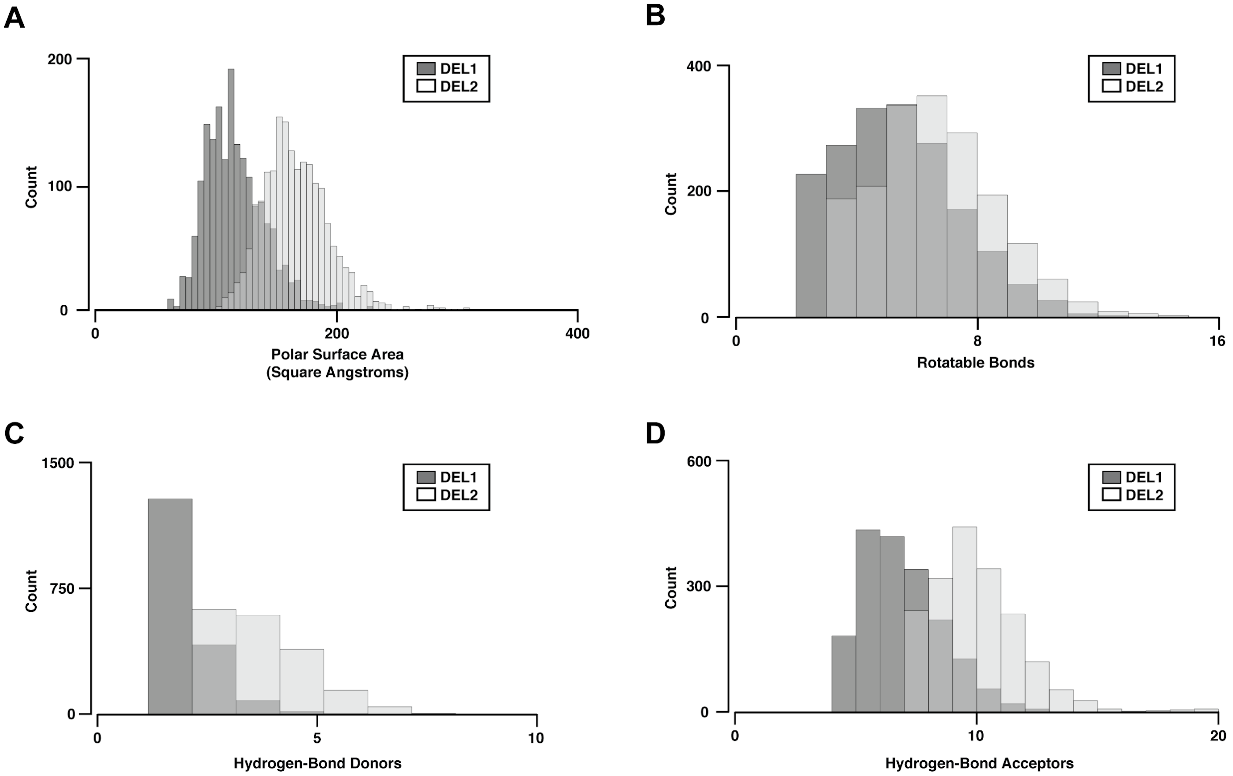

Figure S2. Molecular properties of DEL1 (drug-like) and DEL2 (antibacterial-like / RNA-targeting). (A) polar surface area, (B) rotatable bonds, (C) hydrogen-bond donors, and (D) hydrogen-bond acceptors.

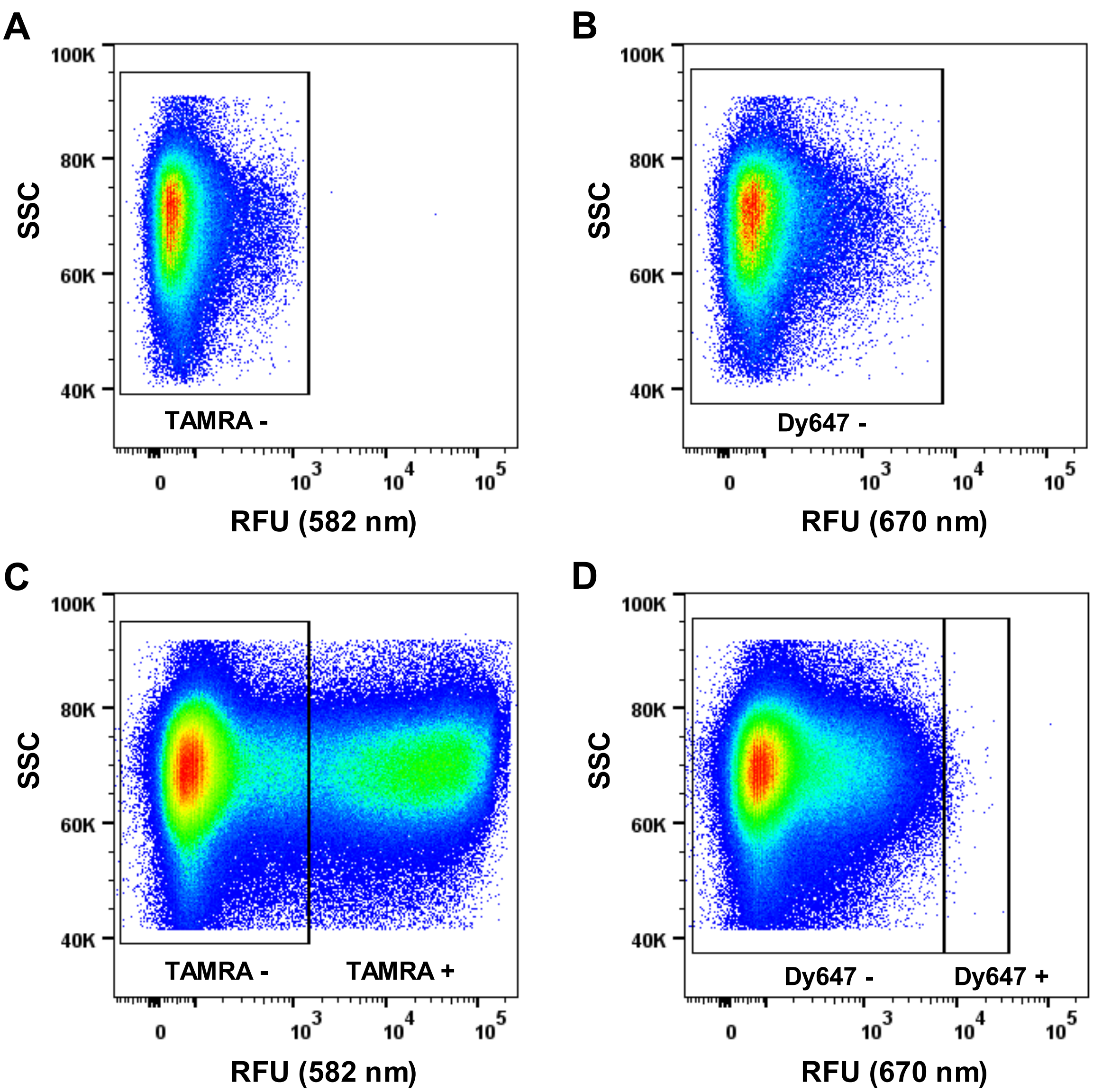

Figure S3. Gating strategy for 2-color FACS analysis of the binding of SPDEL to an RNA motif library labeled with DY647 and a control, fully paired RNA labeled with TAMRA. (A & B) Relative fluorescence units (RFU) at 582 nm and 670 nm vs. side scatter (SSC), respectively, for a SPDEL sample blocked with tRNA and BSA but in the absence of labeled RNAs. (C & D) SPDEL library sample incubated labeled RNAs DY647-3×3 ILL (C) and TAMRA-BP (D) and blocked with tRNA and BSA.

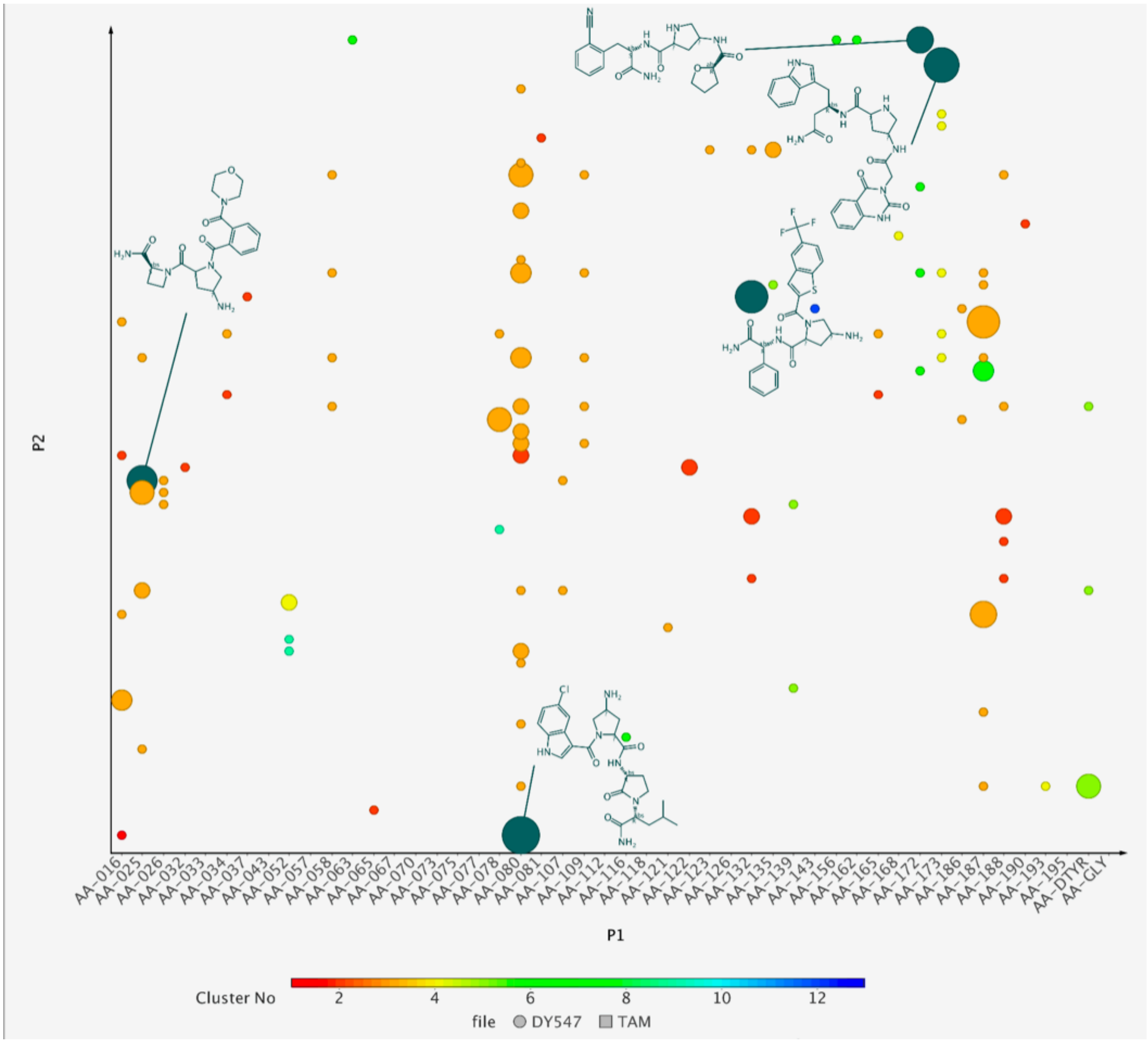

Figure S4. Structure deconvolution of the DNA tags from DEL compound beads that bind 3×3 ILL selectively, as determined by Next-Generation Sequencing (NGS).

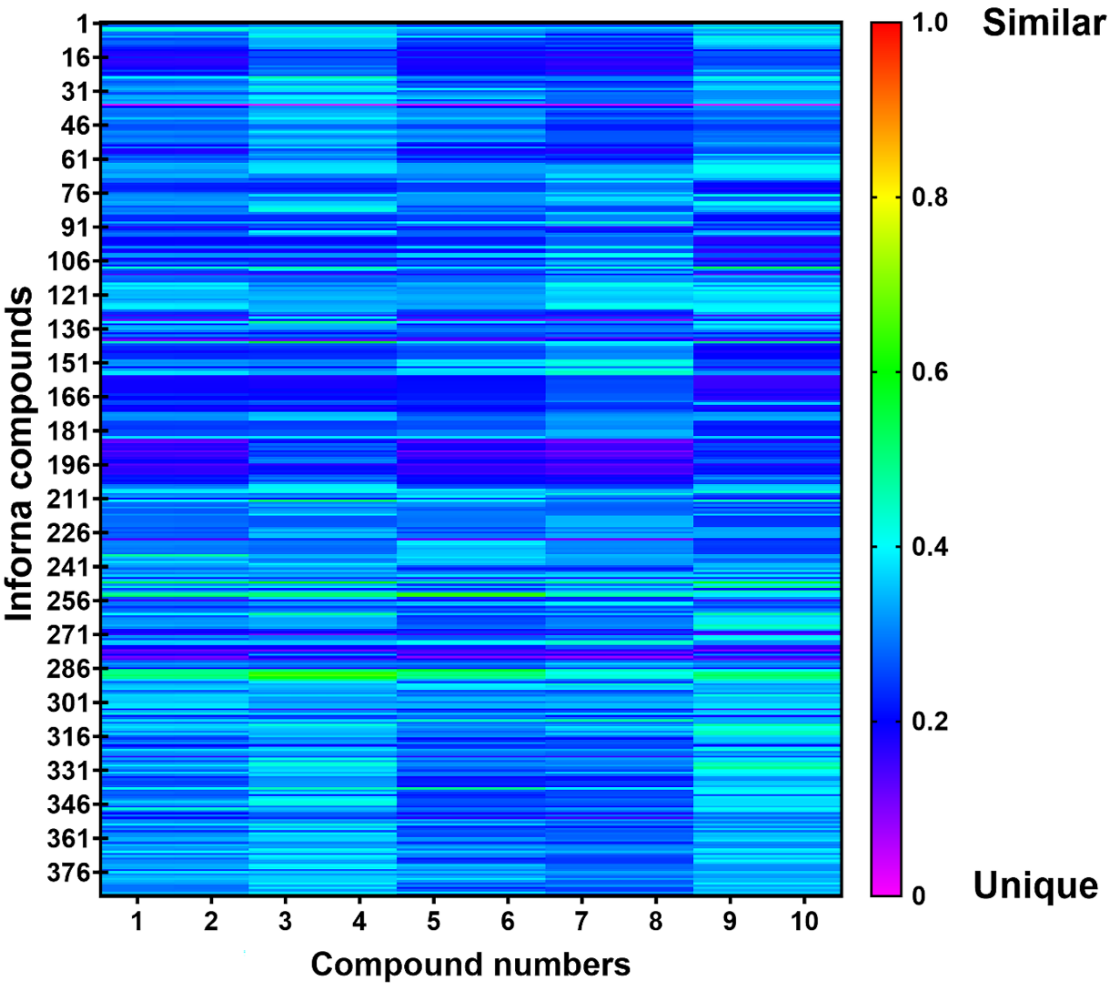

Figure S5. Structural similarity of hit compounds to previously identified RNA-binding small molecules housed in Inforna, as determined by Tanimoto coefficients. Heat map of the Tanimoto scores calculated for **1** – **10** as compared to small molecules in Inforna,(1) a database of all known RNA-binding small molecules reported in the literature by our laboratory and others. The average similarity of the DEL hit compounds compared to Inforna small molecules is 0.3 ± 0.01.

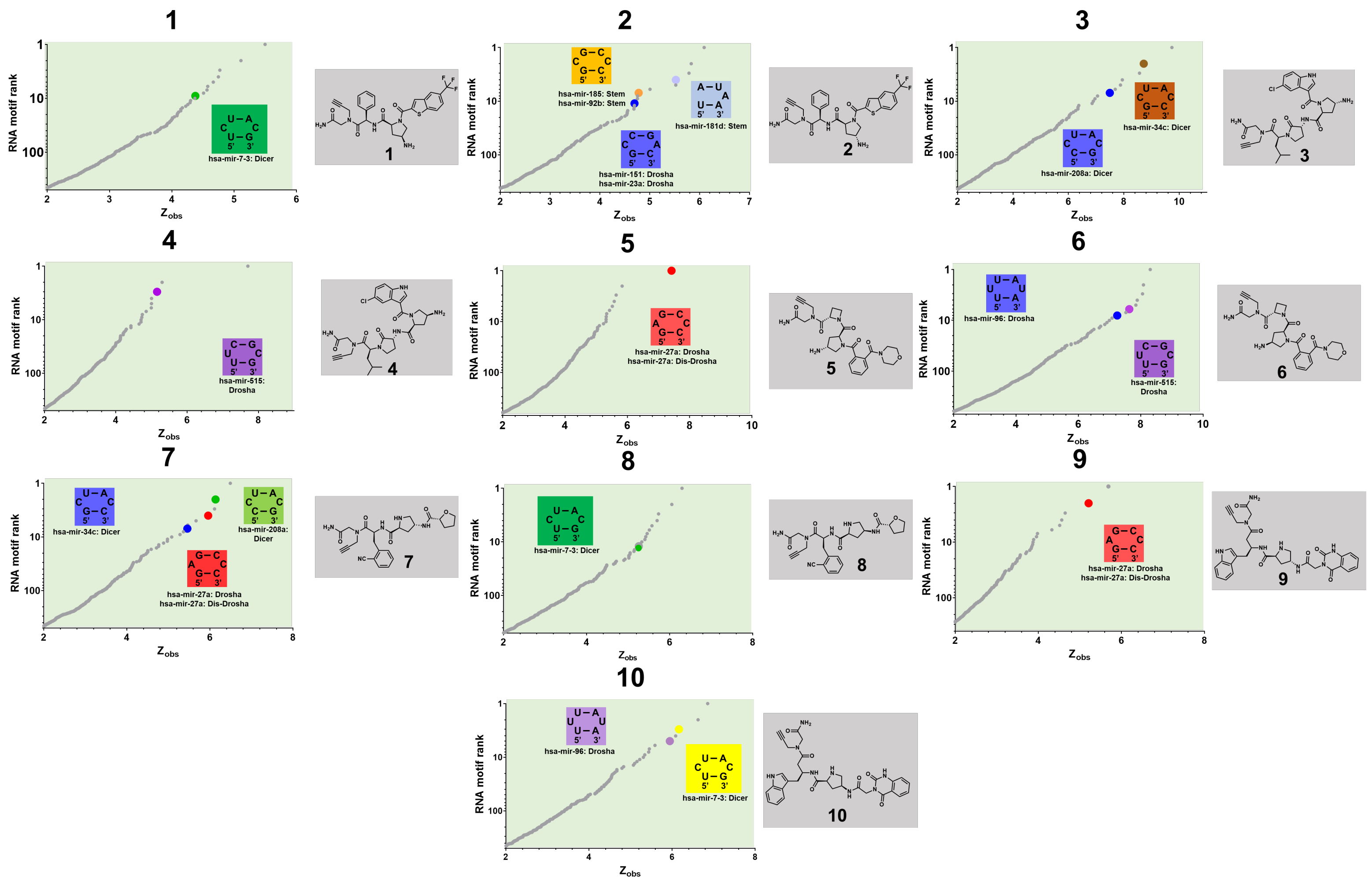

Figure S6. Affinity landscapes for compounds 1 – 10 and the motifs in miRNA Drosha and Dicer processing sites that they bind. Affinity landscapes were generated by plotting the RNA motif's rank in the sequencing data as a function of Z_{obs}. RNA 3D folds present in disease-associated miRNAs are highlighted

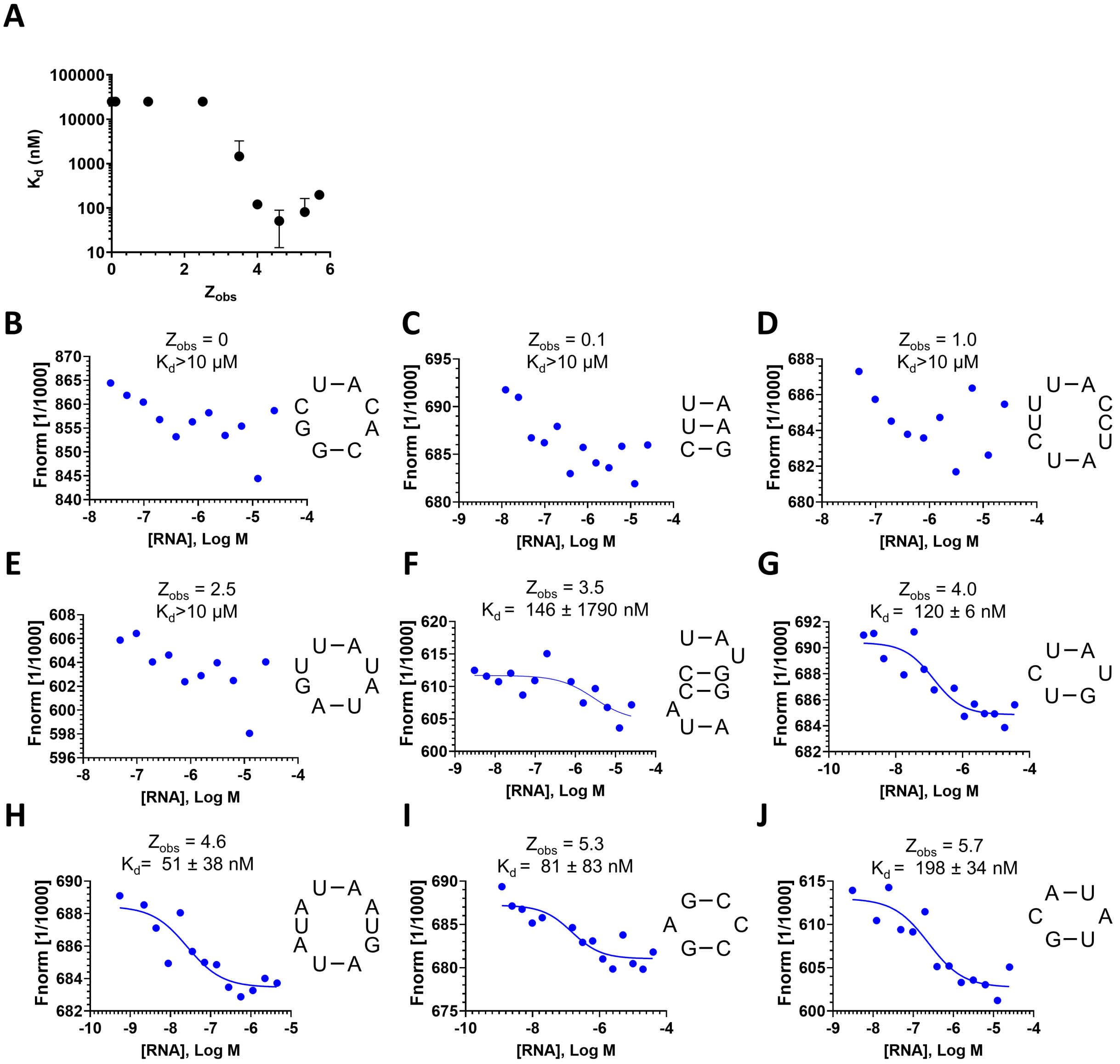

Figure S7. Correlation of binding affinity of compound 9 and Z_{obs}. (A) Summary of binding affinities of **9** for RNA 3D folds as a function of Z_{obs}, measured via competitive MST binding assay using the Cy5-labeled RNA model of miR-27a's Drosha site (**Figures 3 and S11**). Competitive dissociation constants (K_d) are reported as average and standard deviation from two independent experiments. (B-J) Representative MST binding curves for **9** and RNA loops with a range of Z_{obs}.

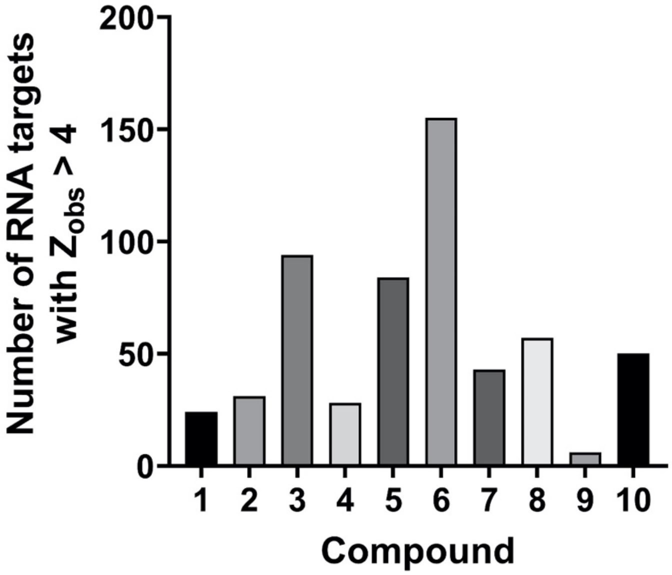

Figure S8. Number of RNA 3D folds that bind each small molecule with Z_{obs} > 4, as determined by Hit-StARTS analysis of the RNA-seq data used to deconvolute the 2DCS selection.

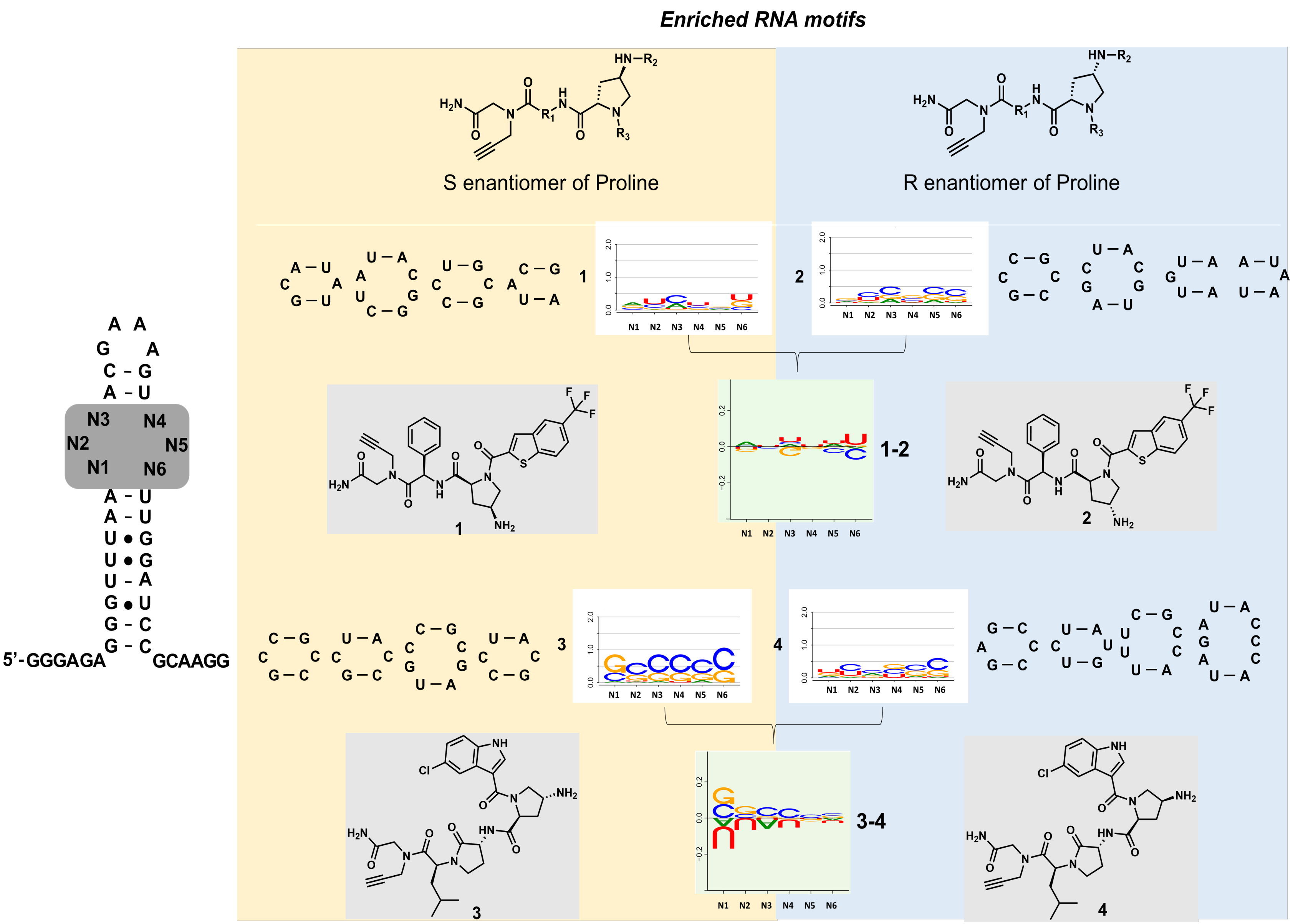

Enriched RNA motifs
S enantiomer of Proline
R enantiomer of Proline
1
2
1-2
3
4
3-4
N1
N2
N3
N4
N5
N6
5'-GGGAGA
GCAAGG

Enriched RNA motifs

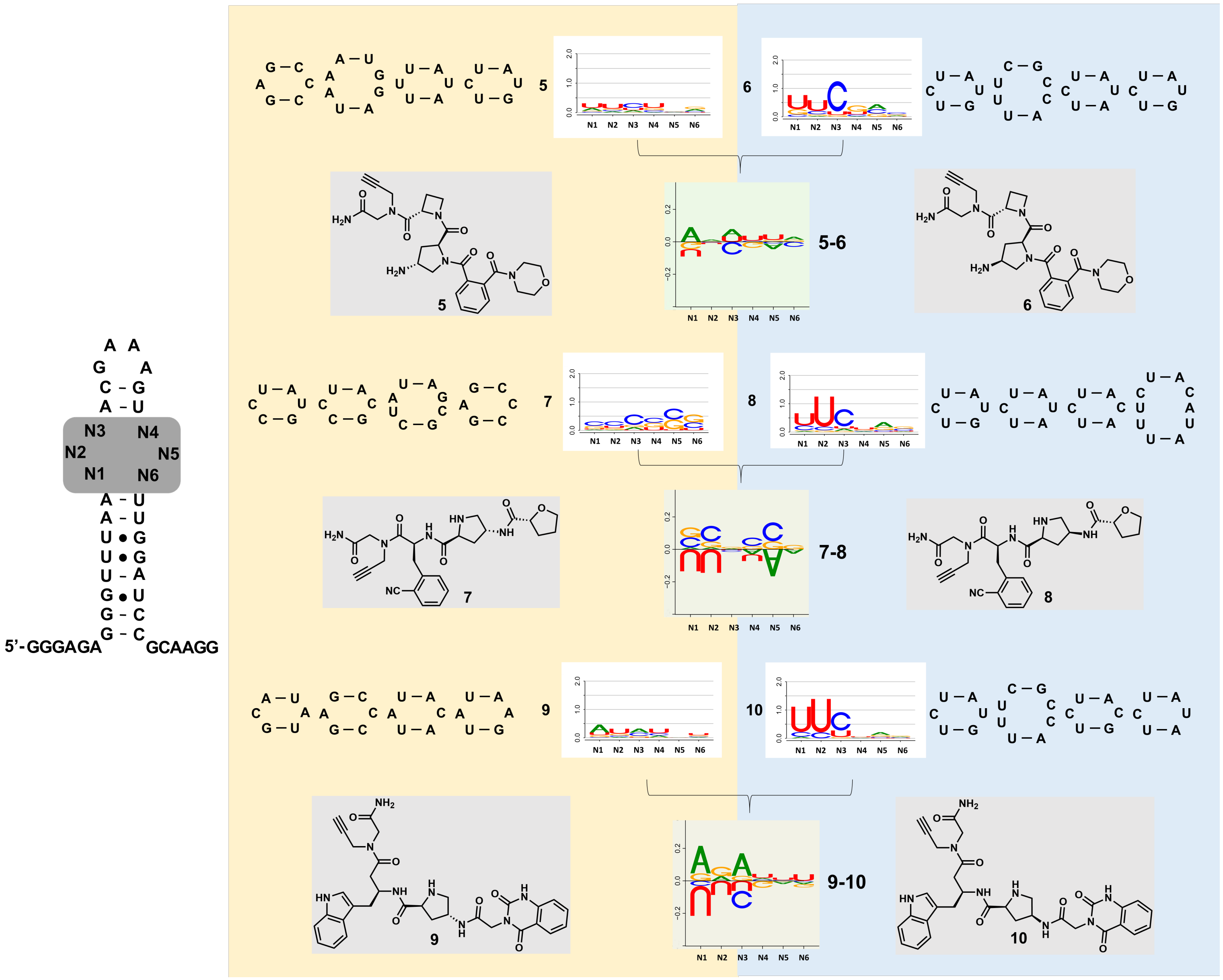

Figure S9. LOGOS analysis of preferred RNA 3D folds for compounds 1 - 10. LOGOS analysis(2) was completed on the RNA 3D folds with the top 0.5% of Z_{obs} score for each compound, and each nucleotide preference in the randomized region is reported as bits. DiffLOGO analysis(3) was also completed on diastereomer pairs and are shown beneath the corresponding LOGOS. The secondary structures of the RNA 3D folds with the four highest Z_{obs} scores are also shown.

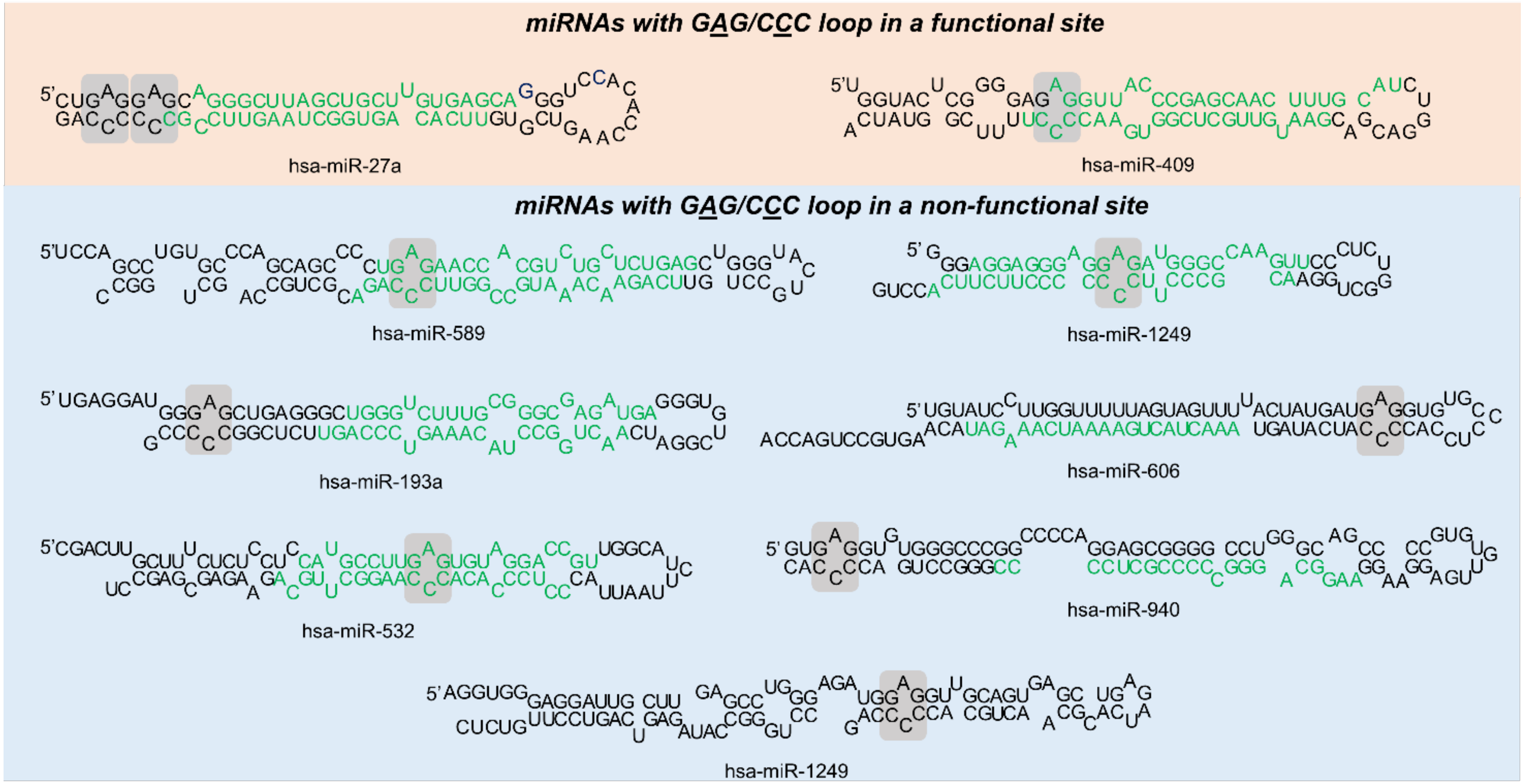

Figure S10. Secondary structures of miRNAs containing 5'GAG/3'CCC internal loops in functional (orange background) and non-functional (blue background) sites.

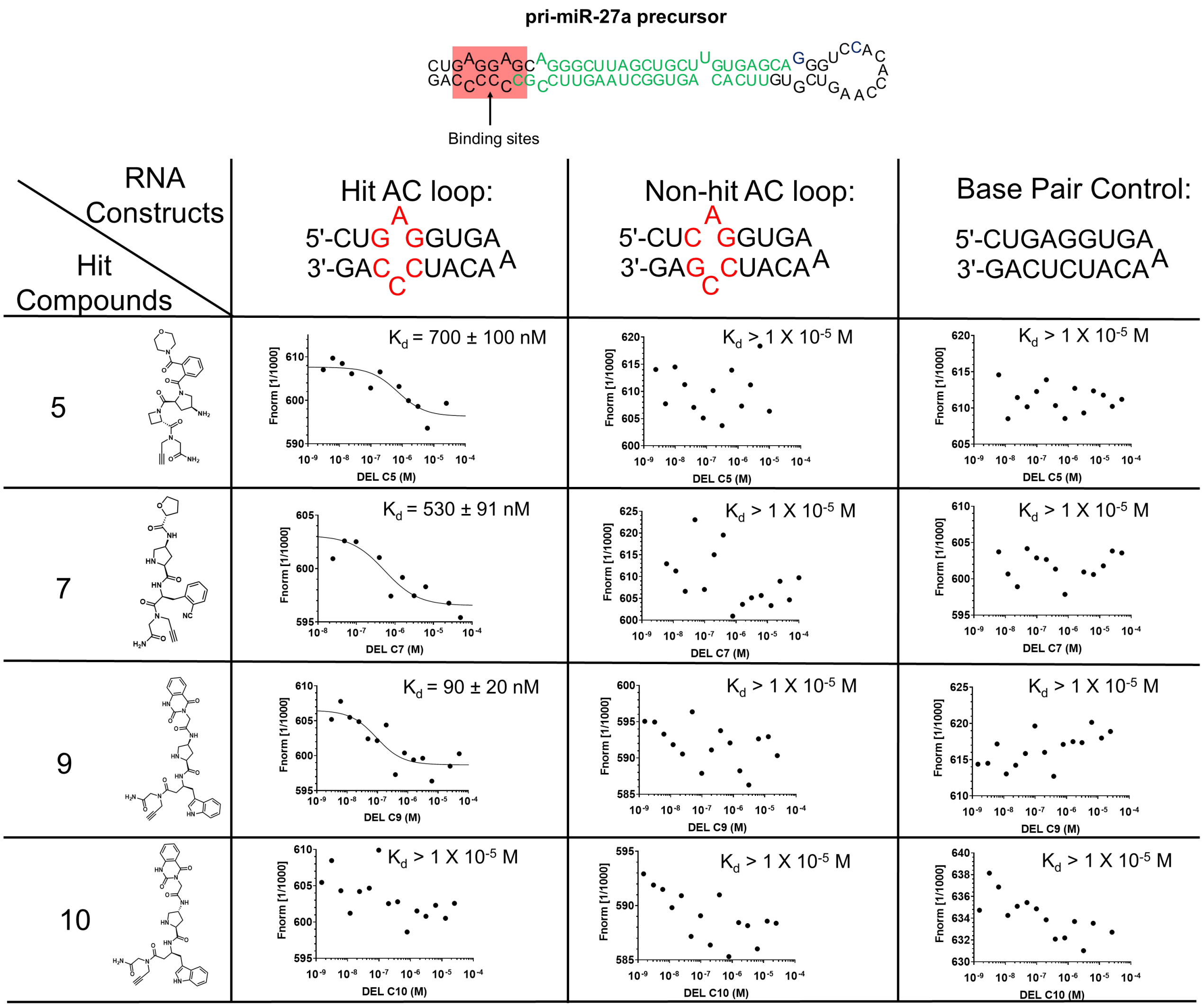

Figure S11. Representative binding affinity curves for 5, 7, 9, and 10 for the 3D fold in pri-miR-27a's Drosha processing site and for control RNAs. Binding affinities were measured by microscale thermophoresis (MST). Error is reported as SD, and the affinity of each interaction is the average of at least 3 independent experiments.

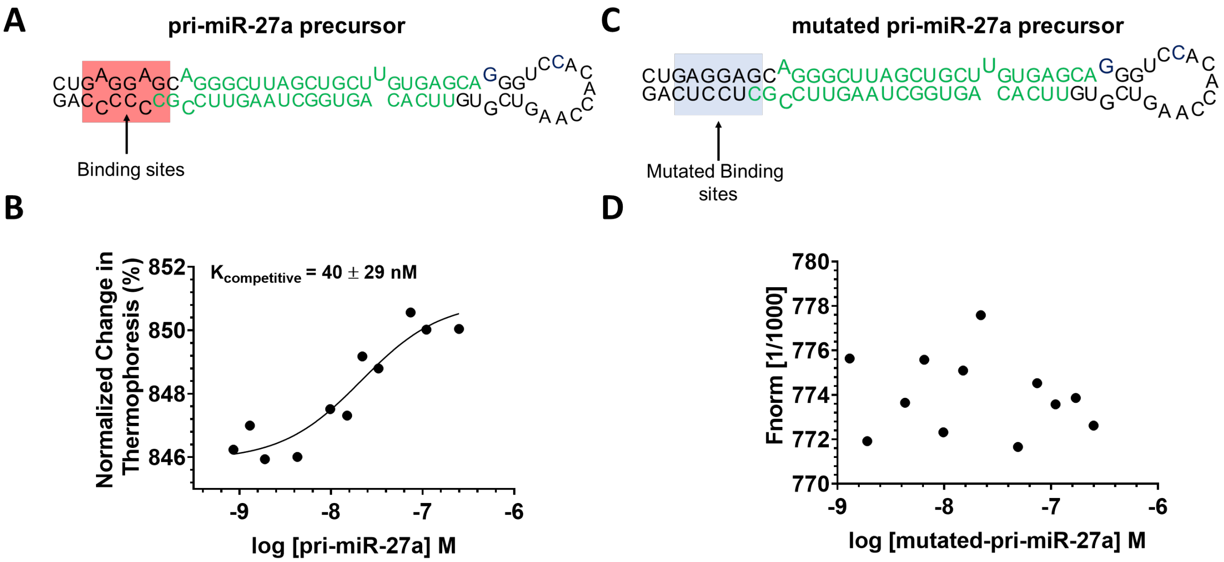

Figure S12. Affinity of 9 for pri-miR-27a and a mutant pri-miR-27a, as determined by a competitive binding assay. (A) Secondary structure of the pri-miR-27a RNA used in a competitive binding experiment with a Cy5-labeled model of its Drosha binding site. (B) Representative binding curve for the competitive binding experiment between **9**, unlabeled pri-miR-27a, and the Cy5-labeled WT Drosha site. (C) Secondary structure of the mutant pri-miR-27a RNA used in a competitive binding experiment with a Cy5-labeled model of WT pri-miR-27a's Drosha binding site. (D) Representative binding curve for the competitive binding experiment between **9**, unlabeled *mutated* pri-miR-27a, and the Cy5-labeled Drosha site. Error is reported as SD, and K_ds are reported as the average of 2 independent experiments.

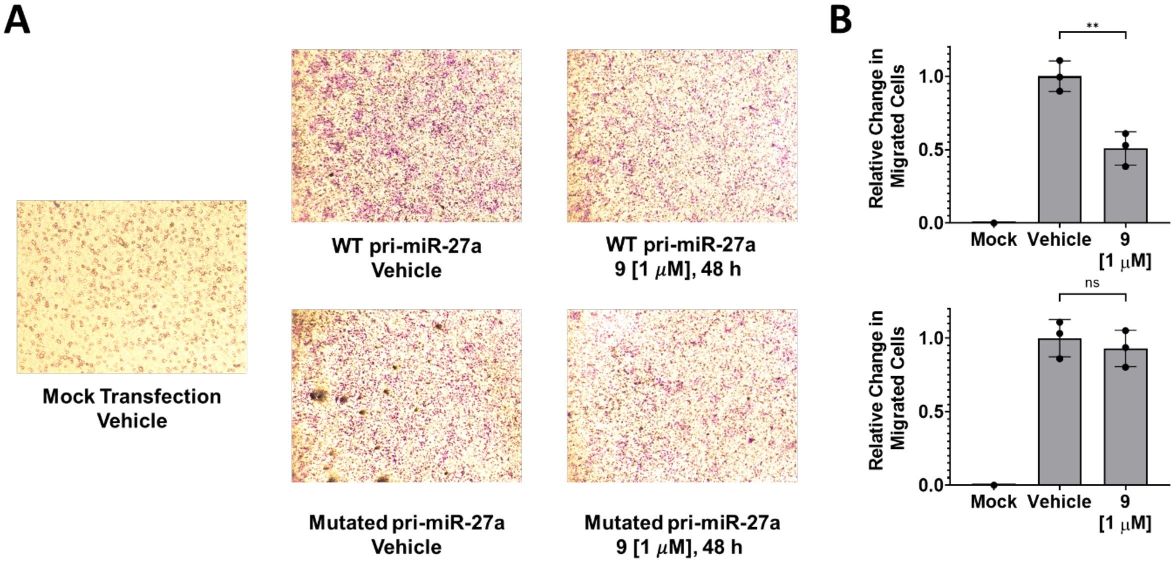

Figure S13. **Pri-miR-27a induces a migratory phenotype in MCF-10a, a cellular model of healthy breast epithelium, that is rescued by 9**. (A) Representative microscopic images of the migratory phenotype induced by WT pri-miR-27a and mutant pri-miR-27a in MCF-10a cells and effect of **9**-treatment. Compound **9** only reduced migration of MCF-10a cells that express WT pri-miR-27a as its binding site is abolished in the mutant pri-miR-27a. (B) Quantification of the number of migratory cells with or without treatment from microscopic images, relative to vehicle-treated samples. Error bars are reported as SD; n = 3 replicates from 2 independent experiments; 3 fields of view analyzed per sample. ***, $P < 0.001$; ****, $P < 0.0001$, as determined by Student t-test compared to “vehicle”.

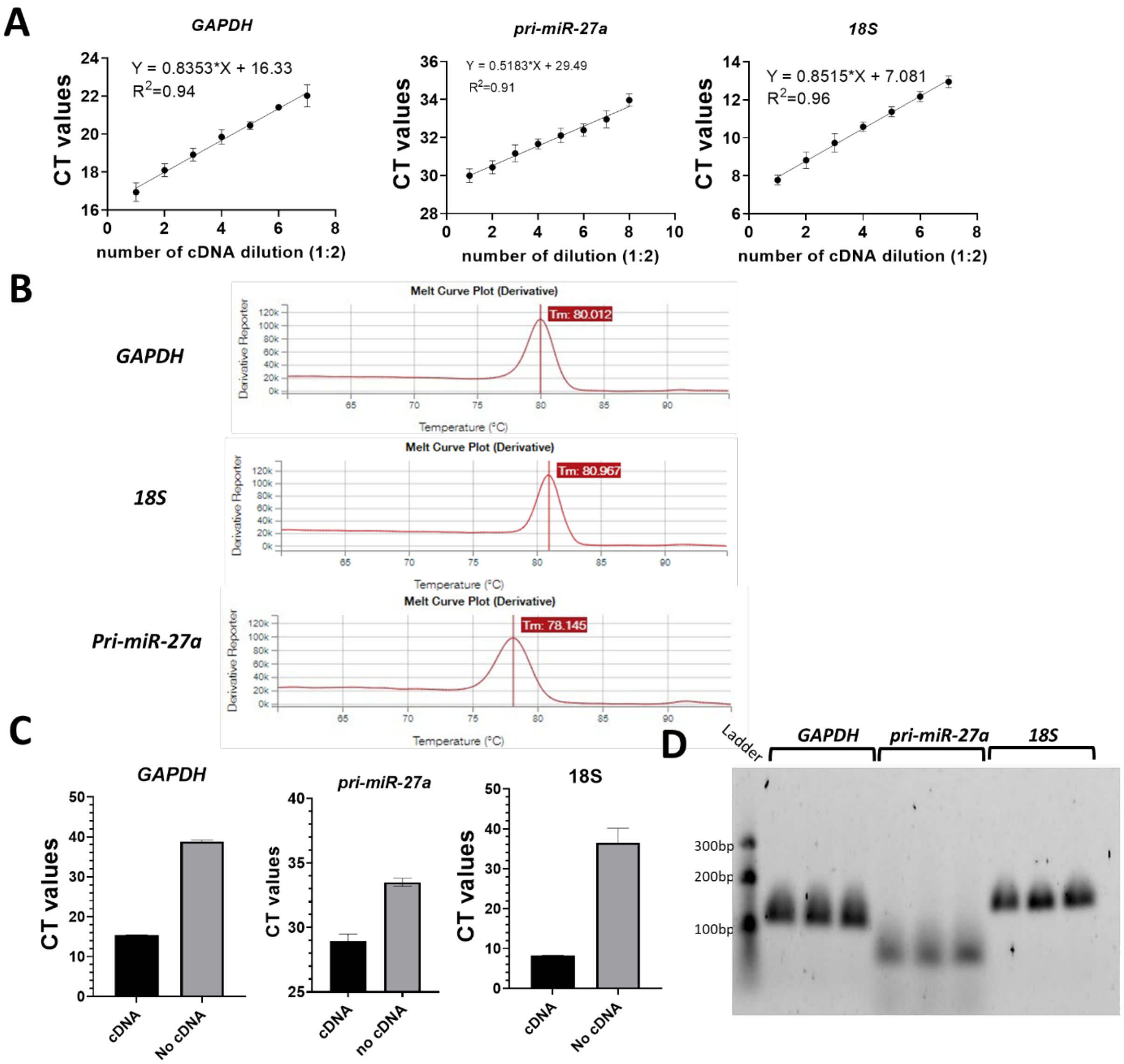

Figure S14. **Validation of RT-qPCR primers used to measure levels of pri-miR-27a and housekeeping (control) genes *GAPDH* and *18S* by RT-qPCR**. (A) C_t values as a function of cDNA dilutions from reverse transcription. (B) Melting curves of RT-qPCR products, supporting that only one species is amplified for each gene. (C) No template control does not amplify by RT-qPCR (C_t values > 32). (D) Gel analysis of RT-qPCR product shows only one band (species) is generated.

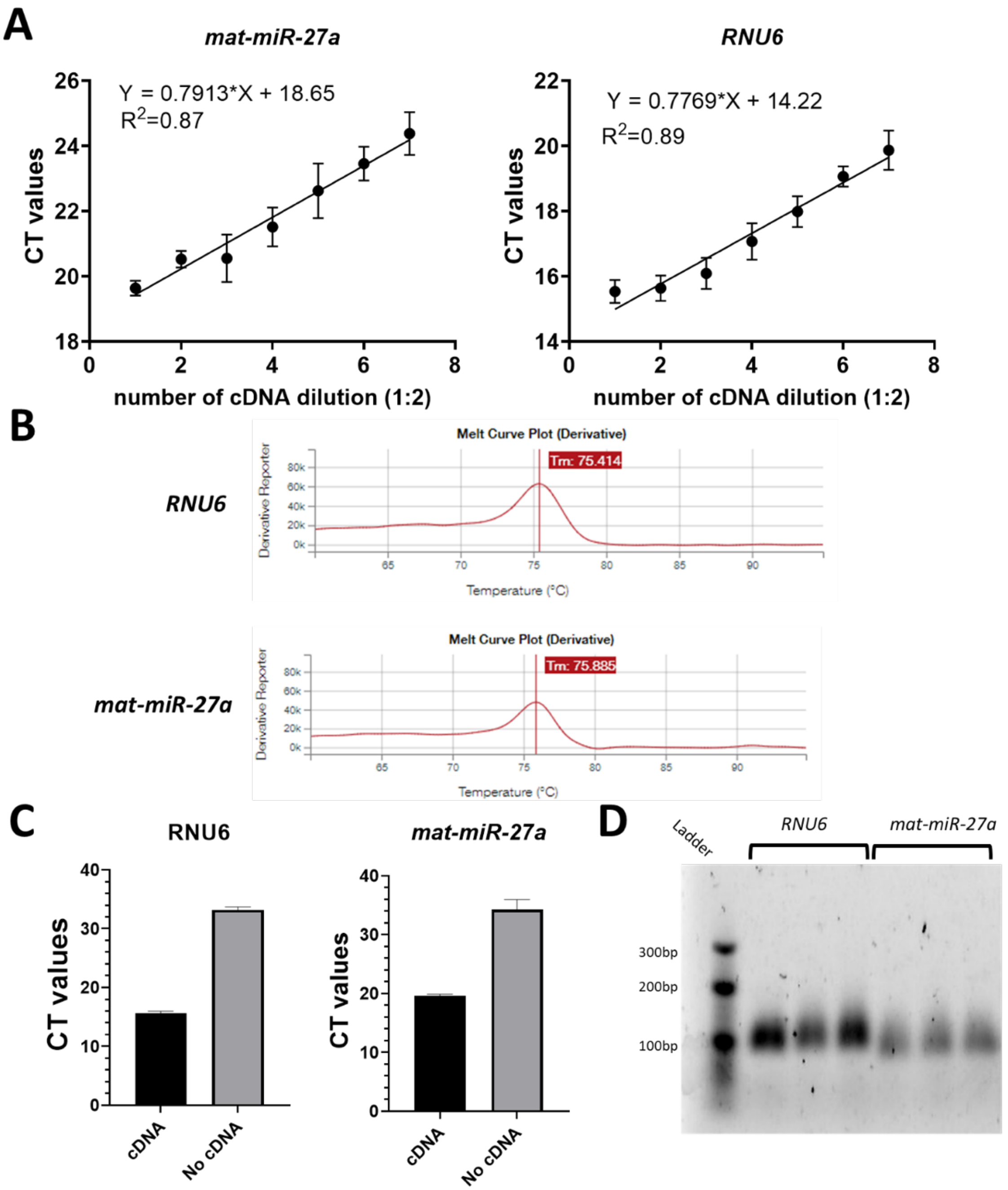

Figure S15. **Validation of RT-qPCR primers used to measure levels of mature miR-27a and housekeeping (control) gene U6 by RT-qPCR**. (A) C_t values as a function of cDNA dilutions from reverse transcription. (B) Melting curves of RT-qPCR products, supporting that only one species is amplified for each gene. (C) No template control does not amplify by RT-qPCR (C_t values > 32). (D) Gel analysis of RT-qPCR product shows only one band (species) is generated.

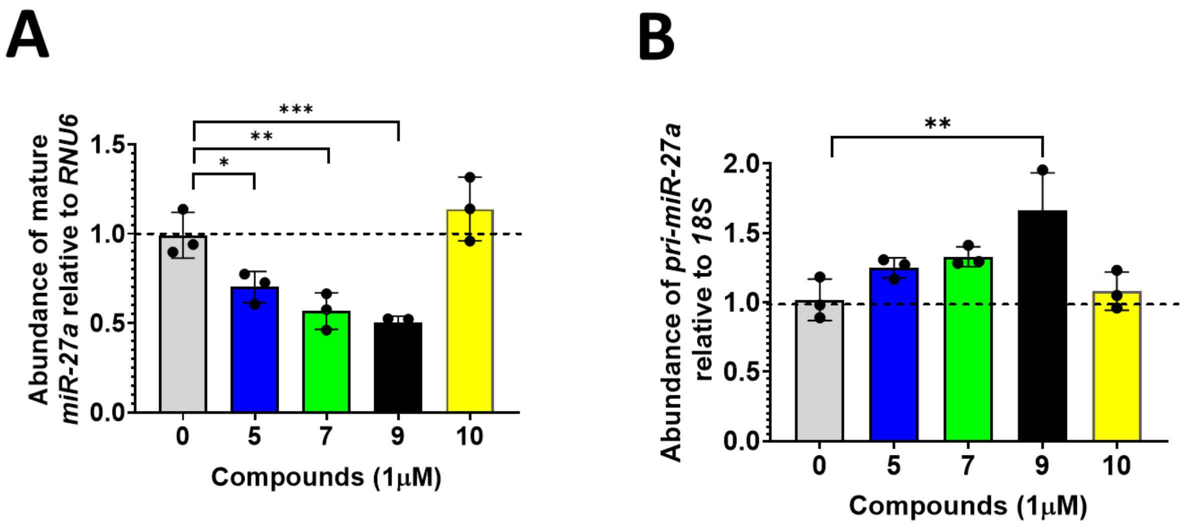

Figure S16. Comparison of 5, 7, 9, and 10 for inhibition of pri-miR-27a biogenesis in MDA-MB-231 cells. (A) Effect of **5, 7, 9,** and **10** (negative control) on mature miR-27a levels, as measured by RT-qPCR. (B) Effect of **5, 7, 9,** and **10** (negative control) on pri-miR-27a levels, as measured by RT-qPCR. Error bars represent SD; n = 3 biological replicates from 2 independent experiments. *, $P < 0.05$; **, $P < 0.01$; ***, $P < 0.001$, as determined by a one-way ANOVA relative to 0 (untreated).

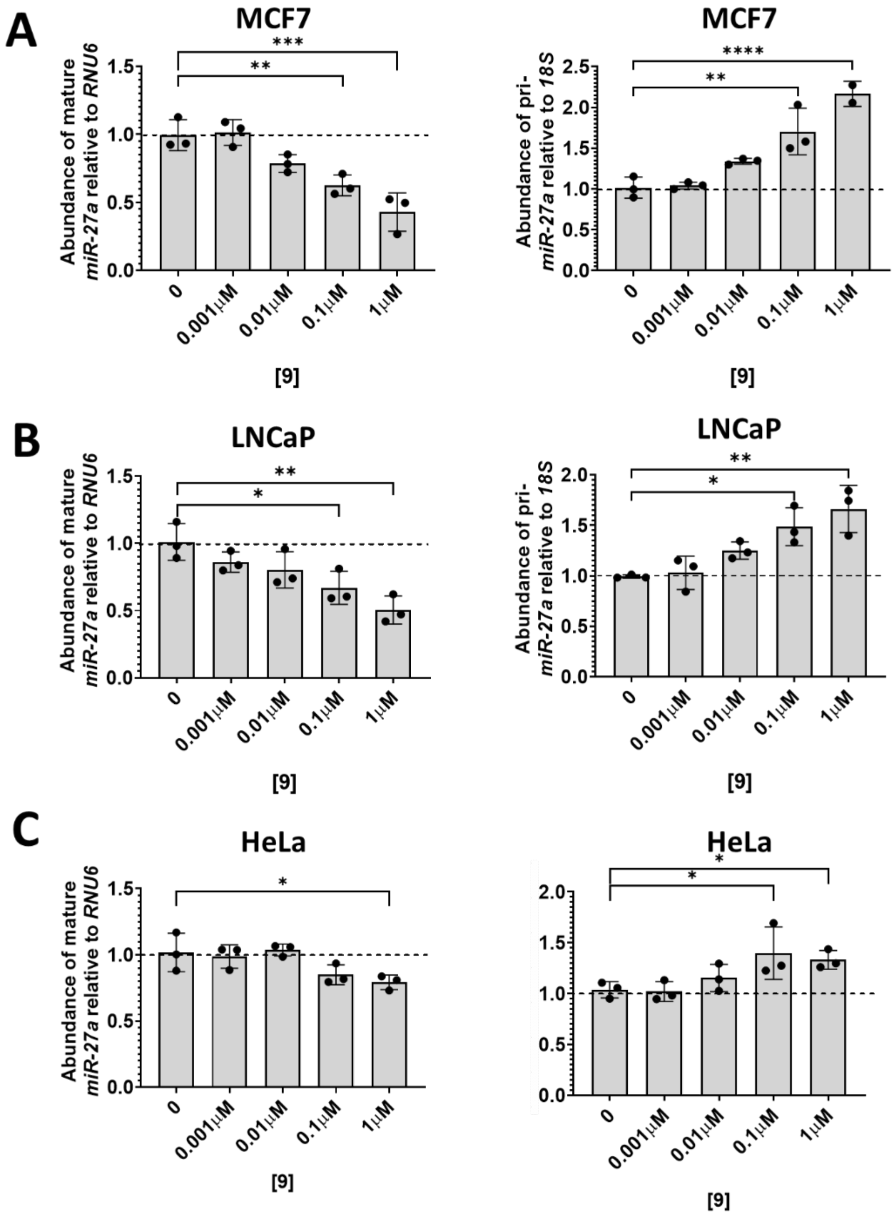

Figure S17. Compound 9 inhibits miR-27a biogenesis in various cancer cell lines. (A) Effect of **9** on mature and pri-miR-27a levels in MCF-7 cells, as determined by RT-qPCR. (B) Effect of **9** on mature and pri-miR-27a levels in LNCaP cells, as determined by RT-qPCR. (C) Effect of **9** on mature and pri-miR-27a levels in HeLa cells, as determined by RT-qPCR. For all panels, error bars represent SD; n = 3 replicates from 2 independent experiments. *, $P < 0.05$; **, $P < 0.01$; ***, $P < 0.001$; ****, $P < 0.0001$, as determined by a one-way ANOVA relative to 0 (untreated cells).

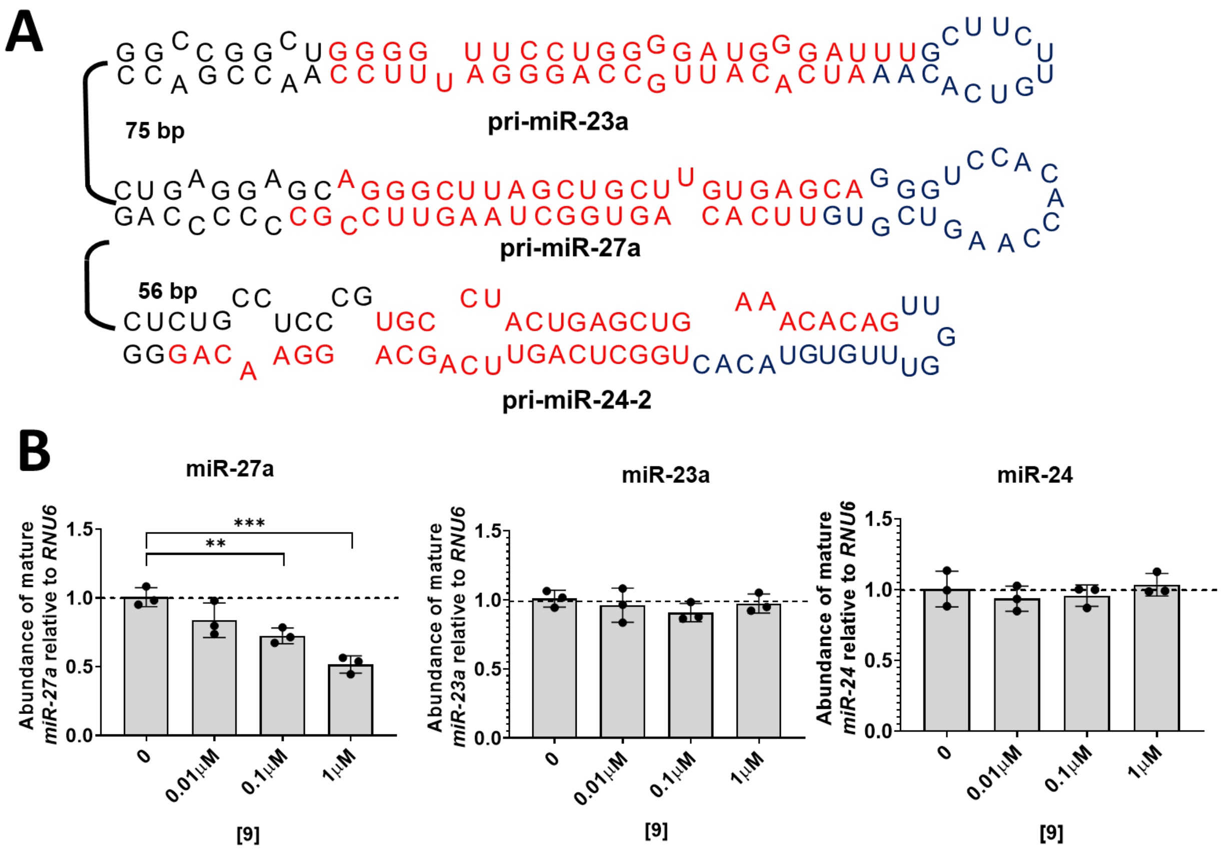

Figure S18. Specificity of 9 for pri-miR-27a vs. miRNAs in its cluster, pri-miR-23a and pri-miR-24. (A) Secondary structure of miR-23a/27a/24 cluster. (B) Effect of **9** on mature miRNA levels in MDA-MB-231 TNBC cells, as determined by RT-qPCR. Error bars represent SD; n = 3 replicates from 2 independent experiments. **, $P < 0.01$, ***, $P < 0.001$, as determined by a one-way ANOVA relative to 0 (untreated).

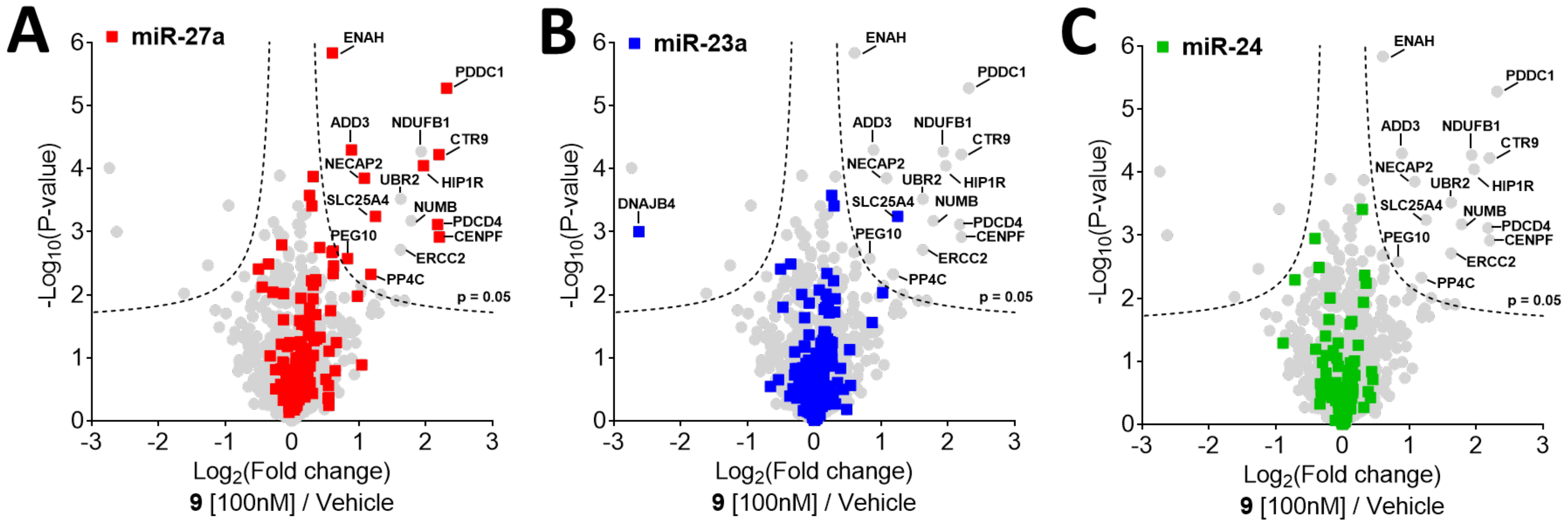

Figure S19. Effect of 9 on protein expression in MDA-MB-231 cells, evaluated by global proteomics analysis. (A) Volcano plot of the effect of **9** on the proteome of MDA-MB-231 cells, as determined by LC-MS/MS analysis, where the downstream targets of miR-27a are highlighted in red. (B) Volcano plot of the effect of **9** on the proteome of MDA-MB-231 cells, as determined by LC-MS/MS analysis, where the downstream targets of miR-23a are highlighted in blue. (C) Volcano plot of the effect of **9** on the proteome of MDA-MB-231 cells, as determined by LC-MS/MS analysis, where the downstream targets of miR-24 are highlighted in green. All downstream targets were predicted by TargetScanHuman v7.2.(4) Data in all plots are represented as Log_2 fold change; dotted lines represent a false discovery rate (FDR) of <5% and an S0 of 0.1 [where S0 is the minimum fold change required to be considered for significance], collectively an adjusted *P*-value of 0.05.

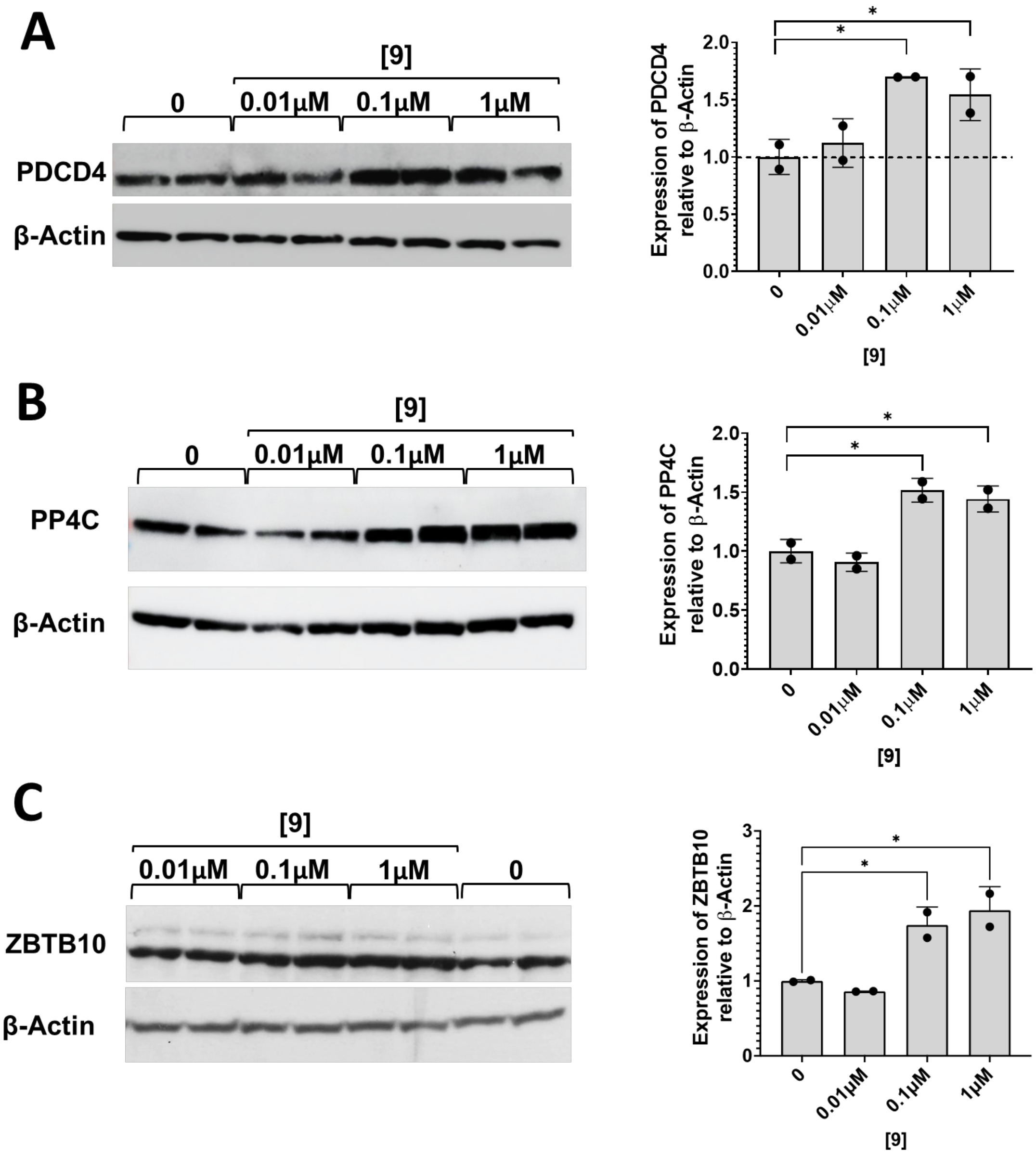

Figure S20. **Treatment of MDA-MB-231 TNBC cells where 9 de-represses three downstream targets of miR-27a, PDCD4, PP4C, and ZBTB10**. (A) Representative Western blot to evaluate the effect of **9** on PDCD4 expression, relative to β-actin, in MDA-MB-231 cells and its corresponding quantification. (B) Representative Western blot to evaluate the effect of **9** on PP4C expression, relative to β-actin, in MDA-MB-231 cells and its corresponding quantification. (C) Representative Western blot to evaluate the effect of **9** on ZBTB10 expression, relative to β-actin, in MDA-MB-231 cells and its corresponding quantification. Error bars represent SD; n = 2 replicates from independent experiments. *, $P < 0.05$ as determined by a one-way ANOVA relative to 0 (untreated cells).

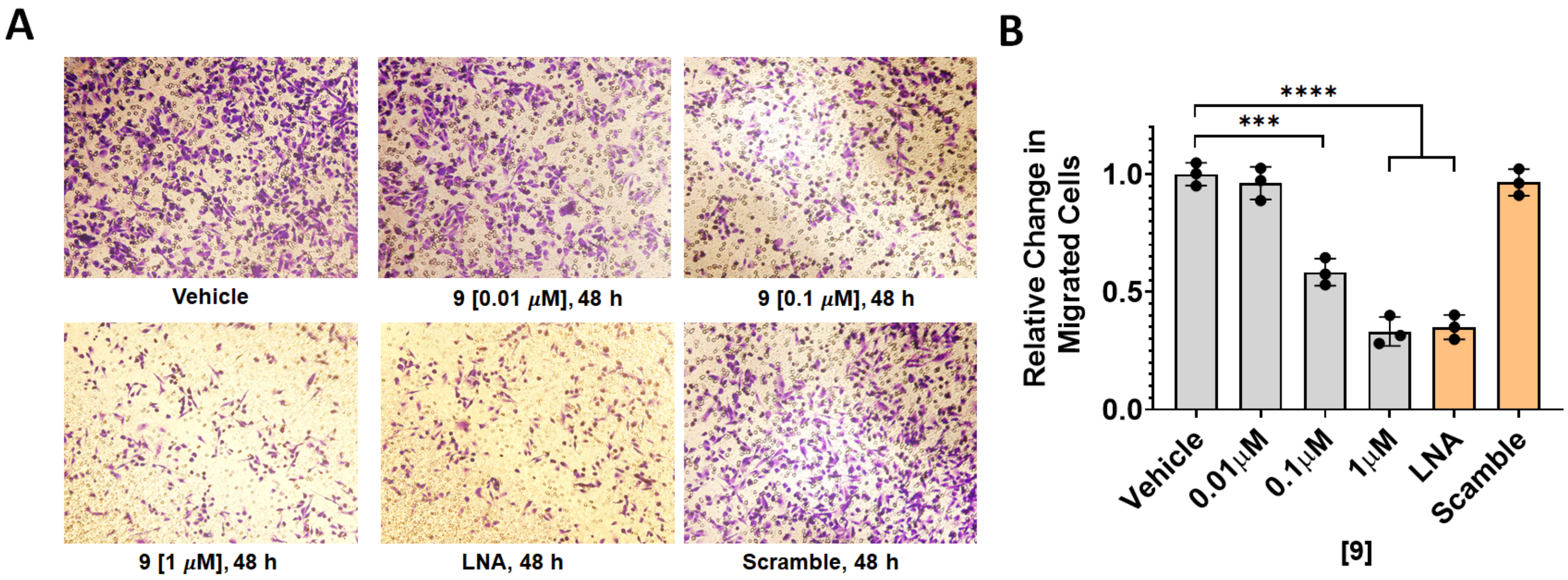

Figure S21. **Compound 9 reduces the miR-27a-mediated migration of MDA-MB-231 TNBC cells**. (A) Representative microscopic images of the effect of **9** at varying doses on the migration of MDA-MB-231, as compared to vehicle-, anti-miR-27a LNA-, and Scramble LNA (negative control)-treated cells. (B) Quantification of the number of migratory cells, relative to vehicle-treated samples. Error bars represent SD; n = 3 replicates from 2 independent experiments; 3 fields of view analyzed per sample. ***, $P < 0.001$: ****, $P < 0.0001$, as determined by a one-way ANOVA relative to "Vehicle".

METHODS

Abbreviations. ACN : acetonitrile, BP : base paired, BSA : bovine serum albumin, COMU : 1-Cyano-2-ethoxy-2-oxoethylidenaminooxy)dimethylamino-morpholino-carbenium hexafluorophosphate , DCM : dichloromethane, DIC : diisopropylcarbodiimide, DIEA : N,N-diisopropylethylamine, DI H_2O : deionized water, DMA : dimethylacetamide, DMEM : Gibco Dulbecco's Modified Eagle Medium, DMF : N, N-dimethylformamide, DMSO : dimethylsulfoxide, DPBS : Dulbecco's phosphate buffered saline, EDTA : ethylenediaminetetraacetic acid, FA : formic acid, FBS : fetal bovine serum, Fmoc : fluorenylmethyloxycarbonyl, FDR : false discovery rate, HATU : 1-[Bis(dimethylamino)methylene]-1H-1,2,3-triazolo[4,5-b]pyridinium 3-oxide hexafluorophosphate, HCCA : α-cyano-4-hydroxycinnamic acid, HDNA : DNA headpiece, HEPES : 4-(2-hydroxyethyl)-1-piperazineethanesulfonic acid, HOAt : 1-Hydroxy-7-azabenzotriazole, HPLC : high-performance liquid chromatography, HRMS : high-resolution mass spectrometry, ILL : internal loop library, LC : liquid chromatography, LC-MS/MS : liquid chromatography coupled with tandem mass spectrometry, MeOH : methanol, MS : mass spectrometry, MST : microscale thermophoresis, NGS : next generation sequencing, OP stock : oligonucleotide paired stock, PAGE : polyacrylaminde gel electrophoresis, PBS : phosphate buffered saline, PCR : polymerase chain reaction, PFA : paraformaldehyde, PTFE : polytetrafluoroethylene, QC : quality control, RPMI : Roswell Park Memorial Institute formulation , SDS : sodium dodecyl sulfate , TBS : tris buffered saline, TBST : tris buffered saline with 0.05% (v/v) Tween-20, TCEP : tris(2-carboxyethyl)phosphine, THPTA : Tris(3-hydroxypropyltriazolylmethyl)amine , TIPS : triisopropylsilane, TMP : 2,4,6-trimethylpyridine, TFA : trifluoroacetic acid, UMI : unique molecular identifier.

Mass Spectrometry. Matrix-assisted laser desorption ionization time-of-flight (MALDI-TOF) mass spectrometry was performed on an AB SCIEX 4800 Plus MALDI-TOF/TOF instrument using

α-cyano-4-hydroxycinnamic acid as matrix. Spectra were acquired using the 4000 Series Explorer software (AbSciex, v 3.2.3) and analyzed using open-source software mMass (v 5.5.0). HRMS spectra were collected by internal calibration using the Mass Standards Kit for Calibration of AB Sciex TOF/TOF TM Instruments (P/N 4333604).

Analytical HPLC. HPLC analyses were conducted on a system composed of Waters 2487 Dual Absorbance Detector and Waters 1525 Binary HPLC Pump equipped with a Sunfire C18 4.8 × 150 mm column. Analyses were conducted with a flow rate of 1 mL/min with a gradient of 0-100% MeOH (+ 0.1% TFA) in water (+ 0.1% TFA) over 40 min followed by 5 min at 100% MeOH (+ 0.1% TFA).

Preparative HPLC. HPLC analyses were conducted on a system composed of Waters 2487 Dual Absorbance Detector, Waters 1525 Binary HPLC Pump and Waters Fraction Collector III. The system was equipped with a Sunfire PREP C18 19 × 150 mm. Analyses were conducted with a flow rate of 5 mL/min with a gradient of 0-100% MeOH (+ 0.1% TFA) in water (+ 0.1% TFA) over 40 min followed by 5 min at 100% MeOH (+ 0.1% TFA).

SOLID-PHASE DEL SYNTHESIS & SCREENING

Materials Sources. All reagents were obtained from MilliporeSigma (St. Louis, MO) unless otherwise specified: 1,3-Bis[tris(hydroxymethyl)methylamino]propane (Bis-Tris), trifluoroacetic acid (TFA), triisopropylsilane (TIPS), tris(2-carboxyethyl)phosphine (TCEP), α-cyano-4-hydroxycinnamic acid (HCCA) (Life Technologies, Carlsbad, CA), N,N'-diisopropylcarbodiimide (DIC, Acrōs Organics, Fair Lawn, NJ), 1-hydroxy-7-azabenzotriazole (HOAt, Accela ChemBio Inc., San Diego, CA), 2,4,6-trimethylpyridine (TMP), ethyl cyanohydroxyiminoacetate (Oxyma), dimethylformamide (DMF, Thermo Fisher Scientific, Waltham, MA), dichloromethane (DCM, Thermo Fisher Scientific), N,N-diisopropylethylamine (DIEA, Thermo Fisher Scientific), acetonitrile (can, Thermo Fisher Scientific), dimethyl sulfoxide (DMSO, AMRESCO Inc., Solon, OH), (4-Fmoc-2-methoxy-5-nitrophenoxy)butanoic acid (Fmoc-PC-OH, Santa Cruz Biotechnology Inc., Dallas, TX), N-α-Fmoc-N-ε-7-methoxycoumarin-4-acetyl-L-lysine (N-α-Fmoc-K(Mca)-OH), Nα-Fmoc-Nω-(2,2,4,6,7-pentamethyldihydrobenzofuran-5-sulfonyl)-L-arginine (N-α-Fmoc-R(Pbf)-OH, Thermo Fisher Scientific), sodium acetate, (GPR, CPC Scientific, Sunnyvale, CA), calcium chloride, Taq DNA polymerase (Taq, New England Biolabs, Ipswich, MA), and 2′-deoxyribonucleotide triphosphate (dNTP, set of dATP, dTTP, dGTP, dCTP, Promega Corp., Milwaukee, WI), were used as provided. Solvents used in solid-phase synthesis were dried over molecular sieves (3 Å, 3.2 mm pellets).

Oligonucleotides (Integrated DNA Technologies, Inc. Coralville, IA) were purchased as desalted lyophilate and used without further purification. Oligonucleotide ligation substrates were 5′-phosphorylated (/5Phos/). The amino-modified precursor DNA piece used for conjugation on beads (NH_2-HDNA; /5Phos/GAGTCA/iSp9//iUniAmM//iSp9/TGACTCCC iSp9 indicates a 9 atom triethylene glycol spacer and iUniAmM indicates an amino-modified six carbon aliphatic spacer) was HPLC purified by the manufacturer and used without further purification. [Note: the HDNA is transformed to a clickable N_3-modified oligonucleotide via acylation w/ azidopentanoic acid.]

Buffers. Bis-Tris propane Wash Buffer (BTPWB, 50 mM NaCl, 0.04% Tween-20, 10 mM Bis-Tris, pH 7.6); 10× Bis-Tris propane Ligation Buffer (BTPLB, 500 mM NaCl, 100 mM $MgCl_2$, 10 mM ATP, 0.2% Tween-20, 100 mM Bis-Tris propane, pH 7.6); 10× PCR Buffer (2 mM dATP, 2 mM dGTP, 2 mM dCTP, 2 mM dTTP, 15 mM $MgCl_2$, 500 mM KCl, 100 mM Tris, pH 8.3), 1× GC-PCR Buffer (1× PCR buffer, 8% (v/v) DMSO, 1 M betaine), and Crush and Soak buffer (C&S, 50 mM NaCl, 10 mM Tris-HCl pH 7.5, 1 mM EDTA). For DEL synthesis, buffers were prepared in deionized water. Otherwise, buffers were prepared in Nanopure H_2O.

Bifunctional library resin synthesis and characterization. The azido-modified headpiece DNA (N_3-HDNA) was prepared as previously described.(5) Linker synthesis proceeded via iterative cycles of solid-phase synthesis. All spin-column wash and reaction volumes were identical (0.4 mL) unless noted. All fritted-syringe wash and reaction volumes were identical (3.0 mL) unless noted. All filtration microplate wash and reaction volumes were identical (0.15 mL) unless otherwise noted.

Quality-control (QC) TentaGel rink amide resin (160 µm, 0.40 mmol/g, 50 mg, Rapp-Polymere, Tuebingen, Germany) was transferred to a fritted spin-column (Mobicol, large filter, 10-µm pore size), swelled in solvent (DMF, 16 h, room temperature, 8 rpm), and washed (3 × DMF). Fmoc was removed (20% piperidine in DMF, 1 × 5 min, 1 × 15 min, room temperature, 8 rpm), and the resin was washed (3 × DMF, 3 × DCM, 3 × DMF). Then, N-α-Fmoc-K(Mca)-OH (60 µmol) was activated (2 min, room temperature) with COMU/DIEA (60/120 µmol) in DMF, added to resin, and the resin was incubated (30 min, 50 °C, 8 rpm, 2 ×). After washing the resin (3 × DMF, 3 × DCM, 3 × DMF), N-α-Fmoc-R(Pbf)-OH (60 µmol; 2 min, room temperature) activated with COMU/DIEA (60/120 µmol) in DMF was added to resin, and the resin was incubated (30 min, 50 °C, 8 rpm). The resin was washed (3 × DMF, 3 × DCM, 3 × DMF), unreacted sites were acetylated

(20% acetic anhydride in DMF, 15 min, 50 °C, 8 rpm), and the resin was washed again (6 × DMF, 6 × DCM, 3 × DMF).

Synthesis resin (amino-functionalized, 10 µm dia., 0.29 mmol/g, 300 mg, Rapp-Polymere) and the aforementioned 160-µm QC resin (30 mg) were transferred to a syringe (6 mL) equipped with a frit (10-µm polyethylene, 13 mm dia., Biotage, Charlotte, NC), swelled in solvent (DMF, 16 h, room temperature, 8 rpm), and washed (3 × DCM, 3 × DMF). Subsequent amino acid coupling cycles consisted of: (1) Fmoc removal (20% piperidine in DMF, 1 × 5 min, 1 × 15 min, room temperature, 8 rpm); (2) N-α-Fmoc-amino acid (1 mmol) activation with DIC/Oxyma/DIEA (1/1/2 mmol, 2 min, room temperature); (3) addition of activated N-α-Fmoc-amino acid to resin and incubation (1 h, 50 °C, 8 rpm). The N-substituted glycine coupling cycle consisted of: (1) Fmoc removal (20% piperidine in DMF, 1 × 5 min, 1 × 15 min, room temperature, 8 rpm); (2) bromoacetic acid (1 mmol) activation with DIC (2 mmol, 2 min, room temperature); (3) coupling of activated bromoacetic acid to resin (1 h , 50 °C, 8 rpm); (4) displacement (1 M propargylamine, 3 h, 50 °C, 8 rpm). Unless specified otherwise, following each Fmoc removal and building block coupling step, resin was washed (3 × DMF, 3 × DCM, 3 × DMF). N-α-Fmoc-Gly-OH, bromoacetic acid/propargylamine, and N-α-Fmoc-Gly-OH were coupled sequentially as described above, but without removing Fmoc from the pendant glycine. Fmoc-PC-OH (0.5 mmol) was activated with DIC/Oxyma/TMP (0.75/0.5/0.5 mmol), added to resin, incubated (1 × 2 h, 1 × 1 h, 37 °C, 8 rpm), and resin was washed (3 × DMF, 3 × DCM, 3 × DMF). Mixed-scale bifunctional-HDNA library resin was prepared and characterized as previously described.(5)

DNA-encoded library resin barcoding. All library synthesis and library handling was performed in a UV-free room. A general protocol for DNA-encoded solid-phase synthesis (DESPS) has previously been described.(5) Oligonucleotides are indicated in bold with the "≈" designation. Numeric identifiers were described previously.(5) Sequences used for DEL construction are listed

in Table S2. Oligonucleotide paired (OP) stock solutions of complementary oligonucleotides (60 μM [+], 60 μM [-], 50 mM NaCl, 1 mM Bis-Tris pH 7.6) were heated (5 min, 60 ℃) and cooled to ambient (5 min, room temperature) before each use. OP stocks bear the [±] designation, indicating "double-stranded." DEL barcoding and encoded combinatorial synthesis procedures are summarized in Scheme S1.

Ligation of ≈0002 and barcoding ≈11XX oligonucleotide. Mixed-scale bifunctional HDNA library resin was split into 192 wells (0.16 mg 160-μm resin, 63 nmol; 1.56 mg 10-μm resin, 450 nmol) of pre-wetted (3 × DCM, 3 × DMF) filtration microplates (2×, Millipore MultiScreen Solvinert 0.45 μm Hydrophobic PTFE), washed (3 × DMF, 3 × 1:1 DMF:BTPWB, 3 × BTPWB), resuspended (BTPWB), covered with adhesive foil (VWR International, Radnor, PA), incubated (1 h, room temperature, 600 rpm), washed (3× BTPWB, 1 × BTPLB), resuspended (BTPLB, 0.1 mL), and incubated while the encoding oligonucleotide ligation mixtures were prepared (~30 min, room temperature). An encoding oligonucleotide ligation mixture containing ≈0002[±] (370 nmol), and T4 DNA ligase (550 μg) in 2.25X BTPLB (20.3 mL) was prepared and aliquoted into all plate wells (100 μL) along with DI H_2O (38 μL). OP stocks of ≈11XX[±] (1.8 nmol, 12 μL) were added to the appropriate wells, and the plate was sealed with adhesive foil and incubated (4 h, room temperature, 600 rpm). Resin was washed (3 × BTPWB), resuspended (BTPWB, 0.15 mL) and incubated (16 h, room temperature, 600 rpm).

DNA-encoded solid-phase combinatorial library synthesis. Barcoded mixed-scale library resin was retrieved, washed (3 × BTPWB, 3 × 1:1 DMF:BTPWB, 3 × DMF), resuspended (DMF, 0.1 mL), pooled into a reservoir, split into 192 wells (0.16 mg 160-μm resin, 63 nmol; 1.56 mg 10-μm resin, 450 nmol) of 2 fresh pre-wetted (3 × DCM, 3 × DMF) filtration microplates, and washed

(2 × DMF). Fmoc was removed (20% piperidine in DMF, 1 × 5 min, 1 × 15 min, room temperature, 600 rpm), washed (3 × DMF, 3 × DCM, 3 × DMA), resuspended (DMA, 0.1 mL), incubated (30 min, room temperature, 600 rpm), and washed (1 × DMA) prior to the first building block coupling. Library synthesis proceeded in eight steps: acylation, Fmoc removal, acylation with Fmoc-azido-proline, Fmoc removal or azide reduction, encoding oligonucleotide ligation, acylation, encoding oligonucleotide ligation, and global Fmoc removal/azide reduction.

Building block couplings. The first and second building block couplings consisted of acylation with an N-Fmoc-protected amino acid, while the third coupling comprised acylation with a carboxylic acid. In the first coupling, resin was resuspended (DMA, 0.15 mL) with building block/HOAt/DIC (6/6/8.5 μmol, respectively). Plates were covered with adhesive foil and incubated (1 h, 37 °C, 600 rpm). Resin was washed (3 × DMA, 3 × DCM, 3 × DMF), resuspended (DMF, 0.1 mL), and incubated (16 h, room temperature, 600 rpm). Resin was retrieved, washed (2 × DMF), Fmoc was removed (20% piperidine in DMF, 1 × 5 min, 1 × 15 min, room temperature, 600 rpm), washed (3 × DMF, 3 × DCM, 3 × DMA), resuspended (DMA, 0.1 mL), and incubated (30 min, room temperature, 600 rpm). The second and third building block couplings proceeded identically except that the second coupling step entailed simultaneous coupling of the cis- and trans-Fmoc-azido-proline isomers. Otherwise, resin was resuspended (DMA, 0.15 mL) with building block/Oxyma/TMP/DIC (12/12/12/15 μmol, respectively). Plates were covered with adhesive foil and incubated (3 h, 37 °C, 600 rpm). Resin was washed (3 × DMA, 3 × DCM, 3 × DMF), resuspended (DMF, 0.1 mL), and incubated (30 min, room temperature, 600 rpm). After the second building block coupling, resin was washed (2 × DMF), Fmoc was removed from half the resin (20% piperidine in DMF, 1 × 5 min, 1 × 15 min, 600 rpm, room temperature) while azide was reduced on the other half of the resin (100 mM TCEP in DMF, 1 h, 37 °C, 600 rpm), resin was washed (3 × DMF, 3 × DCM, 3 × DMF, 3 × 1:1 DMF:BTPWB, 3 × BTPWB), resuspended

(BTPWB, 0.1 mL), and incubated (1 h, room temperature, 600 rpm). After the third building block coupling, an identical deprotection procedure was followed, except that all resin was subjected to both the Fmoc removal and azide reduction conditions as above.

Ligation of ≈22XX and ≈13XX encoding oligonucleotides. Resin was retrieved, washed (2 × BTPWB, 1 × BTPLB), resuspended (BTPLB, 0.1 mL), and incubated (30 min, room temperature, 600 rpm). An encoding oligonucleotide ligation mixture containing T4 DNA ligase (540 µg) in 2.25X BTPLB (20 mL) was prepared and aliquoted into all plate wells (0.1 mL) along with DI H2O (26 µL). OP stocks of ≈22XX[±] (1.8 nmol, 12 µL) and ≈13XX[±] (1.8 nmol, 12 µL) were then added to the appropriate wells, the plate was sealed with adhesive foil, and incubated (4 h, room temperature, 600 rpm). Resin was washed (3 × BTPWB, 3 × 1:1 DMF:BTPWB, 3 × DMF), resuspended (DMF, 0.1 mL) and incubated (16 h, room temperature, 600 rpm). Resin was pooled into a reservoir, split into 192 wells (0.16 mg 160-µm resin, 63 nmol; 1.56 mg 10-µm resin, 450 nmol) of two fresh pre-wetted (3 × DCM, 3 × DMF) filtration microplates, and washed (3 × DMF, 2 × DMA) prior to coupling the third building block set.

Ligation of ≈24XX and ≈15XX encoding oligonucleotides. Ligation proceeded identically to above, except that OP stocks of ≈24XX[±] and ≈15XX[±] were used. Following ligation, pooling, and splitting, resin was washed (3 × BTPWB, 3 × 1:1 DMF:BTPWB, 3 × DMF), resuspended (BTPWB, 0.1 mL), and incubated (1 h, room temperature, 600 rpm) prior to the final ligation step.

Ligation of barcoding ≈26XX and ≈0B02 encoding oligonucleotides. An encoding oligonucleotide ligation mixture containing ≈0B02[±] (360 nmol) and T4 DNA ligase (550 µg) in 2.25X BTPLB (20 mL) was prepared and aliquoted into plate wells (0.1 mL) along with DI H2O

(38 µL). OP stocks of ≈26XX[±] (1.8 nmol, 12 µL) were added to the appropriate wells, the plates were sealed with adhesive foil, and incubated (4 h, room temperature, 600 rpm). Resin was washed (3 × BTPWB, 3 × 1:1 DMF:BTPWB, 3 × DMF), resuspended (DMF, 0.1 mL), pooled, 160-µm QC beads were isolated by filtration (CellTrics 150 µm mesh, Sysmex Partec, Lincolnshire, IL), and stored in the dark (DMF, 4 °C).

Solid-phase DEL QC. An aliquot of 10-µm resin (0.06 mg) was transferred to a 1.5-mL tube, washed (BTPWB, 3 × 0.5 mL), and resuspended (BTPWB, 0.5 mL). The 10-µm bead concentration was determined by hemocytometer and diluted (1.2 beads/µL). The 160-µm QC resin was washed (10 × BTPWB), resuspended (BTPWB, 1 mL), and an aliquot (3 mg) was separated for analysis.

Resin cleavage and MALDI-TOF MS analysis. Individual 160-µm beads (MeOH, 0.1 mL) were dried in vacuo (60 ℃). A cleavage cocktail (90% TFA, 5% TIPS, 5% DCM, 10 µL) was added to dried single 160-µm bead samples, incubated (2 h, room temperature, 100 rpm), and dried in vacuo (60 ℃). Compound was resuspended (50% ACN, 0.1% TFA in H_2O; 6 µL), a diluted (1:10) aliquot (1 µL) was co-spotted onto a MALDI-TOF MS target plate with HCCA matrix solution, dried, and analyzed via MALDI-TOF MS (Microflex, Bruker Daltonics, Inc., Billerica, MA) (**Table S3**).

DEL FACS screening for specific binding to DY-647-3×3 ILL: An aliquot of approximately 7.5 x10^6 DEL beads (100-fold greater than the number of unique compounds in the DEL) was filtered and then washed (i) once with *N,N*-dimethylformamide (DMF); (ii) once with 1:1 DMF:water; (iii)

three times with Nanopure water; and (iv) three times with BTPWB. The beads were then equilibrated in BTPWB for 30 min at room temperature. The beads were counted using a hemocytometer under a microscope, diluted to a final concentration of 1×10^6 beads/mL in 1× Blocking Buffer (BTPWB + 200 nM BSA + 200 nM bulk yeast tRNA + 1% (v/v) Tween-20), and incubated overnight with shaking (300 rpm) at room temperature. The beads were then briefly vortexed and separated in two samples, a blank sample to establish a negative gate and establish thresholds (2.5×10^6 beads) and a screening sample to screen for RNA binding (5×10^6 beads).

Stocks of DY647-3×3 ILL (20 µM) and TAMRA-BP (200 µM) in Nanopure water were folded by heating at 60 °C for 5 min and cooled at room temperature for 5 min prior to use. To the screening sample, 5 µL of the TAMRA-BP stock was added to a final concentration of 200 nM, and the beads were briefly agitated. Then, 5 µL of the DY647-3×3 ILL stock was added to a final concentration of 20 nM. An equal volume of Nanopure water (10 µL) was added to the blank sample used for negative gating. The samples (blank and screening) were then incubated for 2 h with gentle shaking (300 rpm) at room temperature. They were filtered by using M1002 Mobicol Classic columns (Boca Scientific), washed three times with BTPWB, and resuspended in BTPWB at final concentration of 5×10^6 beads/mL. Finally, the beads were filtered into a 5 mL round-bottom tube through a cell-strainer cap and subjected to FACS on the BD FACSAria3 (BD Biosciences). At least 8×10^5 beads were analyzed for sorting to cover the library diversity with a redundancy of 10. Beads showing an increase of DY647 fluorescence without any increase of TAMRA fluorescence were isolated and pooled together in a 1.6 mL tube. Beads were washed five times with water, with centrifugation at 5000 rpm for 5 min and careful removal of the supernatant between washes.

qPCR analysis. qPCR analysis proceeded as previously described with modifications to the 160-µm bead DNA amplification procedure. qPCR matrix for 10-µm beads contained Taq DNA

Polymerase (0.05 U/μL), oligonucleotide primers 5′-GCCGCCGCCTTCGTCCTTCTCAGCGAC-3′ and 5′-/5AmMC6/GTGGCACAACAACTGGCGGGCAAAC-3′ (0.3 μM each), SYBR Green (0.2×, Life Technologies), and GC-PCR buffer (1×). Diluted (1.2 beads/μL) and undiluted (100 beads/μL) 10-μm library beads (BTPWB, 1 μL) were added to separate amplification wells containing qPCR matrix (20 μL, 33 and 10 replicates, respectively). The supernatant for each resin sample (1 μL) was added to separate amplification wells (20 μL, 2 replicates). Template standard solutions (1 fmol, 100 amol, 10 amol, 1 amol, 100 zmol, 10 zmol, 1 zmol, 100 ymol, and 10 ymol, each in 1 μL BTPWB) were added to separate amplification reactions (20 μL). Reactions were thermally cycled (96 °C, 10 s; [95 °C, 8s; 72 °C, 24 s] × 32 cycles; 72 °C, 2 min); with fluorescence monitoring (channel 1, CFX96 Real-Time System, Bio-Rad) and quantitated (CFX Manager, Version 3.1, Bio-Rad, baseline subtracted). The number of amplifiable tags per bead was calculated by dividing the qPCR result by the number of beads per well (confirmed using a stereo zoom microscope). Amplification proceeded identically for 160-μm beads, except that individual beads were added to separate wells for amplification, the qPCR matrix contained 0.6 μM 5′-/5AmMC6/GTGGCACAACAACTGGCGGGCAAAC-3′, and the reaction thermal cycling was modified (96 °C, 10 s; [95 °C, 8s; 72 °C, 120 s] × 29 cycles; 72 °C, 2 min).

Amplification and sequencing. Single 160-μm resin beads (33) were retrieved via pipet from PCR plate wells and deposited into a 96-well microplate (MeOH, 0.1 mL). Each 160-μm library bead PCR sample (5 μL) was purified by native PAGE (6%, 1× TBE, 12 W, 30 min). Gel slices containing 182-nt DNA products were excised and eluted prior to amplification (C&S, 0.1 mL, 16 h, room temperature, 8 rpm). PCR matrix contained Taq DNA Polymerase (0.05 U/μL), oligonucleotide primers 5′-GTTTTCCCAGTCACGAC-3′ (0.3 μM) and 5′- GTGGCACAACAACTG-3′ (0.28 μM) and 5′-CGCCAGGGTTTTCCCAGTCACGACCAACCACCCAAACCACAAA CCCAAACCCCAAACCCAACACACAACAACAGCCGCCGCCTTCGTCCTTCTCAGCGAC-3′

(0.02 μM, FOX primer), and GC-PCR buffer (1×). PAGE-purified PCR products (2 μL) were added to separate amplification reactions (50 μL) and thermally cycled (95 °C, 2 min; [95 °C, 20 s; 52 °C, 15 s; 72 °C, 20 s] × 34 cycles; 72 °C, 2 min). PCR products were purified (QIAquick PCR purification kit, QIAGEN, Valencia, CA) and sequenced using the primer 5'-CGCCAGGGTTTTCCCAGTCACGAC-3'. Sequencing reads were trimmed to remove all called bases prior to the opening primer sequence (5′-GCCGCCCAGTCCTGCTCGCTTCGCTAC-3′). Sequences were aligned to a degenerate reference sequence (5′-ATGGNNNNNNNNTCANNNNNNNNGTTNNNNNNNNCTANNNNNNNNTTCNNNNNNNNCGC NNNNNNNNGCCTCCCAAACNNNNNNNNGTT-3′) and the encoding regions (5′-NNNNNNNN-3′) were matched to the building block alpha-numeric identifier lookup table to assign the synthesis history for each compound.

Hit amplification and preparation for NGS. Samples for NGS analysis were prepared as previously described.(5, 6) The qPCR matrix was prepared containing Taq DNA polymerase (0.05 U/μL), oligonucleotide primers 5′-GCCGCCGCCTTCGTCCTTCTCAGCGAC-3′ (0.3 μM) and 5′-/5AmMC6/GTGGCACAACAACTGGCGGGCAAAC-3′ (0.6 μM), SYBR Green (0.2×, Life Technologies), and GC-PCR buffer (1X). qPCR matrix was added to 0.2 mL tubes (40 μL). Template standard solutions (100 amol, 10 amol, 1 amol, 100 zmol, 10 zmol, 1 zmol, 100 ymol, and 10 ymol, each in 1 μL BTPWB) were added to separate amplification reactions (40 μL). Hit beads (n = ~130; 0.017% hit rate) were washed (2 × 200 μL BTPWB, 1 × 200 μL PCR buffer) and resuspended in qPCR matrix (40 μL). Reactions were thermally cycled (96 °C, 10 s; [95 °C, 8s; 72 °C, 120 s] × 29 cycles; 72 °C, 2 min). Samples were centrifuged (5 s, 2,000 rcf), then the supernatant was collected and diluted (10,000-fold). PCR matrix contained Taq DNA polymerase (0.05 U/μL), oligonucleotide primer 5′-CCTCTCTATGGGCAGTCGGTGATGCCGCCGCCTTCGTCCTTCTCAGCGAC-3′ (0.3 μM),

sequencing barcode oligonucleotide primer 5′-CCATCTCATCCCTGCGTGTCTCCGACTCAGNNNNNNNNNNNGATGCCGCCCAGTCCTGCTCGCTTCGCTAC-3′ (0.3 μM), SYBR Green (0.2×, Life Technologies) DMSO (6%), betaine (1 M), $MgCl_2$ (1 mM) and PCR buffer (1×). Amplicon supernatant (2 μL) and a corresponding sequencing barcode oligonucleotide primer (5′-CCATCTCATCCCTGCGTGTCTCCGACTCAGNNNNNNNNNNNGATGCCGCCCAGTCCTGCTCGCTTCGCTAC-3′, 0.3 μM) were added to separate amplification wells (40 μL). Reactions were thermally cycled ([95 °C, 8 s; 70 °C, 24 s; 72 °C, 16 s] × 20 cycles; 72 ℃, 2 min). Amplicons were pooled and purified (25 μL) by native PAGE (6%, 1 × TBE, 8 W, 30 min) with SYBR Gold staining (Life Technologies, Inc.). Gel bands containing the 210-bp DNA products were excised, eluted (DI H2O, 0.1 mL, 16 h, room temperature, 8 rpm), and used for standard DNA sequencing library preparation and analysis (Ion Proton, Life Technologies, Inc.).

NGS data processing. Sequence trimming, pattern matching, and UMI aggregation proceeded as previously described. (5, 6) Sequences were ranked by UMI mean string distance and UMI count. Beads with mean string distance < 5 and UMI count < 10 were not considered. Compound replicates were calculated as the sum of remaining sequences having identical structure-encoding regions but distinct bead-specific barcodes.(7)

Cheminformatic analysis. All in silico combinatorial library and hit cheminformatic analysis was performed using open-access software (DataWarrior v 4.7.2).(8) Hits were clustered by chemical similarity (T > 0.75).

Synthesis of the hit compounds: Hit compounds emerging from the FACS screen were synthesized in parallel on Rink Amide Polystyrene resin (50 mg, 27.5 μmol). The resin was swollen in mL DMF for 10 min, filtered, and deprotected with 1 mL of 20% piperidine in DMF twice for 5 min each. The resin was washed (5 x DMF) followed by bromoacetylation with 1 mL of a cocktail containing 20% DIC and 80% 1.2M bromoacetic acid in DMF for 30 min. After washing (5 × DMF), bromide displacement was accomplished by treatment with 1 mL of 1 M propargylamine dissolved in DMF for 1 h, followed by additional washing (5 × DMF).

The first building block (Fmoc-Xaa-OH) coupling was performed in DMF (160 mM, 1 mL) in the presence of HOAt (160 mM) and DIC (114 mM) for 1 h at 37 °C. After washing (5 × DMF), the resin was treated with 1 mL of 20% piperidine in DMF twice for 5 min each, then washed again (5 × DMF). Fmoc-protected azidoproline (3S or 3R) was coupled as described for the previous coupling. Then, resin was either treated with A) 20% piperidine in DMF twice for 5 min each or B) TCEP (100 mM, 80% DMF and 20% water) for 1 h at 37 °C, depending on the deconvoluted regiochemistry, then washed (A : 5 × DMF; B : 2 × H_2O, 5 × DMF).

The last building block (R-COOH) was coupled in DMF (320 mM, 1 mL) in the presence of Oxyma (320 mM), DIC (200 mM) and TMP (160 mM) for 1 h at 37 °C. After washing (5 × DMF), the resin was either treated with A) 20% piperidine in DMF twice for 5 min each or B) TCEP (100 mM, 80% DMF and 20% water) for 1 h at 37 °C, depending on the deconvoluted regiochemistry, then washed (A: 5 × DMF, 5 × DCM; B: 2 × H_2O, 5 × DMF, 5 × DCM). The compounds were cleaved from the resin with a cocktail containing 95% TFA, 2.5% water, and 2.5% TIPS. The cleavage cocktail was evaporated under a stream of nitrogen to afford the crude compounds. Compounds were solubilized and purified by preparative HPLC as described above. Fractions containing the expected mass were pooled and evaporated to dryness under vacuum prior to full characterization.

Compound **1** was synthesized using the general procedure above following deconvoluted synthesis history: cas # 111524-95-9, cas # 263847-08-1, piperidine, cas # 244126-64-5, TCEP.

After HPLC purification 5.8 μmol of **1** was obtained (21% yield). ($C_{28}H_{26}F_3N_5O_4S$) calculated $[M+H]^+$: 586.1731 Da; found: 586.1646 Da (14 ppm); t_R: 36.0 min.

Compound **2** was synthesized using the general procedure above following deconvoluted synthesis history: cas # 111524-95-9, cas # 702679-55-8, piperidine, cas # 244126-64-5, TCEP. After HPLC purification 14.9 μmol of **1** was obtained (54% yield). ($C_{28}H_{26}F_3N_5O_4S$) calculated $[M+H]^+$: 586.1731 Da; found: 586.1566 Da (28 ppm); t_R: 35.9 min.

Compound **3** was synthesized using the general procedure above following deconvoluted synthesis history: cas # 145484-45-3, cas # 263847-08-1, piperidine, cas # 10406-05-0, TCEP. After HPLC purification 3.8 μmol of **1** was obtained (14% yield). ($C_{29}H_{36}ClN_7O_5$) calculated $[M+H]^+$: 598.2539 Da; found: 598.2488 Da (8 ppm); t_R: 34.8 min.

Compound **4** was synthesized using the general procedure above following deconvoluted synthesis history: cas # 145484-45-3, cas # 702679-55-8, piperidine, cas # 10406-05-0, TCEP. After HPLC purification 11.4 μmol of **1** was obtained (41% yield). ($C_{29}H_{36}ClN_7O_5$) calculated $[M+H]^+$: 598.2539 Da; found: 598.2350 Da (32 ppm); t_R: 34.0 min.

Compound **5** was synthesized using the general procedure above following deconvoluted synthesis history: cas # 136552-06-2, cas # 263847-08-1, piperidine, cas # 73728-40-2, TCEP. After HPLC purification 16.0 μmol of **1** was obtained (62% yield). ($C_{26}H_{32}N_6O_6$) calculated $[M+H]^+$: 525.2456 Da; found: 525.2299 Da (30 ppm); t_R: 17.9 min.

Compound **6** was synthesized using the general procedure above following deconvoluted synthesis history: cas # 136552-06-2, cas # 702679-55-8, piperidine, cas # 73728-40-2, TCEP. After HPLC purification 17.7 μmol of **1** was obtained (64% yield). ($C_{26}H_{32}N_6O_6$) calculated $[M+H]^+$: 525.2456 Da; found: 525.2225 Da (44 ppm); t_R: 17.9 min.

Compound **7** was synthesized using the general procedure above following deconvoluted synthesis history: cas # 401933-16-2, cas # 263847-08-1, TCEP, cas # 87392-05-0, piperidine. After HPLC purification 3.2 μmol of **1** was obtained (12% yield). ($C_{25}H_{30}N_6O_5$) calculated $[M+H]^+$: 495.2228 Da; found: 495.2228 Da (25 ppm); t_R: 22.8 min.

Compound **8** was synthesized using the general procedure above following deconvoluted synthesis history: cas # 401933-16-2, cas # 702679-55-8, TCEP, cas # 87392-05-0, piperidine. After HPLC purification 5.7 μmol of **1** was obtained (21% yield). ($C_{25}H_{30}N_6O_5$) calculated $[M+H]^+$: 495.2228 Da; found: 495.2242 Da (22 ppm); t_R: 22.2 min.

Compound **9** was synthesized using the general procedure above following deconvoluted synthesis history: cas # 353245-98-4, cas # 263847-08-1, TCEP, cas # 78754-94-6, piperidine. After HPLC purification 6.5 μmol of **1** was obtained (24% yield). ($C_{32}H_{34}N_8O_6$) calculated $[M+H]^+$: 627.2639 Da; found: 627.2639 Da (5.5 ppm); t_R: 31.3 min.

Compound **10** was synthesized using the general procedure above following deconvoluted synthesis history: cas # 353245-98-4, cas # 702679-55-8, TCEP, cas # 78754-94-6, piperidine. After HPLC purification 8.1 μmol of **1** was obtained (29% yield). ($C_{32}H_{34}N_8O_6$) calculated $[M+H]^+$: 627.2639 Da; found: 627.2833 Da (25 ppm); t_R: 29.9 min.

IN VITRO METHODS

2DCS: Preparation of small molecule microarrays and RNA selection: *Preparation of azide functionalized glass slides*: Briefly, a 2 mL aliquot of 1% (w/v) molten agarose solution (prepared in Nanopure water) was applied to a silane-coated glass slide, and the agarose was allowed to dry overnight. The slides were then immersed in 20 mM $NaIO_4$ and gently shaken at room temperature for 30 min. The slides were washed with Nanopure water twice for 15 min each and then immersed in 10% (v/v) ethylene glycol and shaken at room temperature for 1.5 h. After washing the slide twice in Nanopure water twice for 15 min each, they were immersed in 20 mM azido propylamine prepared in 0.1 M $NaHCO_3$ and shaken at room temperature overnight. The slides were then reduced by immersing the slides in a solution of 100 mg $NaBH_3CN$ in 10 mL ethanol and 40 mL 1× PBS for 30 min at room temperature. The slides were then washed twice with Nanopure water for 15 min each and dried completely on the benchtop.

Conjugation of compounds to azido-functionalized microarrays: Compounds at varying concentrations (0.5 μL in DMSO) were combined with an equal volume of "Click Reaction Mixture" comprised of $CuSO_4$ (10 mM, 0.1 μL), THPTA (50 mM, 0.1 μL), sodium ascorbate (250 mM, 0.1 μL) and phosphate buffer (0.2 μL, 20 mM sodium phosphate, pH 7.5). This mixture was then spotted onto the array surface, and the array was incubated at 37 °C for 3 h in a humidity chamber. After 3 h, the slides were washed with Nanopure water twice and allowed to dry completely on the benchtop.

2DCS selection: The 3×3 ILL was 5'-end labeled with ^{32}P and purified as previously described.(9) The RNA library was folded in 1× Binding Buffer (BB1; 8 mM Na_2HPO_4, pH 7.0, 185 mM NaCl, 1 mM EDTA) by heating at 60 °C for 10 min followed by cooling to room temperature on the bench top. All competitor oligos (C1-C8), each in an amount equivalent to the number of total compound delivered to the array surface, were folded separately in 1× AB1 as described for 3×3 ILL. The folded oligos were mixed together with 5'-^{32}P labeled 3×3 ILL

followed by addition of $MgCl_2$ (1 mM) and bovine serum albumin (BSA, 120 µg/mL) in a total volume of 600 µL. The array surface was preequilibrated with 1× BB2 (1× BB1 supplemented with 1 mM $MgCl_2$ and 120 µg/mL BSA) for 5 min, after which the excess buffer was removed. The mixture of 3×3 ILL and competitor oligonucleotides was then applied to the surface, and the array was incubated for 20 min at room temperature. The glass slide was then washed with 1× AB2 three times and dried for 1 h. The array was imaged by using Molecular Dynamics Typhoon variable mode phosphorimager.

Reverse transcription and PCR amplification to install barcodes to encode each compound were performed as previously described.(10) The DNA thus obtained was purified using native 8% polyacrylamide gel electrophoresis (PAGE) and its purity confirmed via bioanalyzer. The bar-coded samples were mixed in equimolar amounts and sequenced using an Ion Proton deep sequencer. The sequencing data obtained were statistically analyzed for enrichment according to previously reported protocol, affording Z_{obs} for each small molecule-RNA interaction.(10)

Binding affinity measurements: Binding affinities were measured by microscale thermophoresis (MST), performed on a Monolith NT.115 system (NanoTemper Technologies) with Cy5-labeled RNAs. These RNAs include: the 5'GAG/3'CCC loop at pri-miR-27a's Drosha site (Sequence:/5'-Cy5/rCrUrGrArGrGrUrGrArArArCrArUrCrCrCrArG; Dharmacon), a related internal loop not selected by **9**, 5'CAG/3'GCC loop (Sequence:/5'-Cy5/rCrUrCrArGrGrUrGrArArArCrArUrCrCrGrArG; Dharmacon), and an RNA in which the loop is mutated to a base pair (Sequence:/5-Cy5/rCrUrGrArGrGrUrGrArArArCrArUrCrUrCrArG; Dharmacon).

Briefly, Cy5-labeled RNA (10 nM) was prepared in 1× Binding Buffer and folded by heating at 60 °C for 5 min and then slowly cooling to room temperature. After cooling, Tween-20 was added to a final concentration of 0.1% (v/v). Compound solutions were prepared separately in 20

µL of 1× Binding Buffer at a final concentration of 5 µM (1% (v/v) DMSO), followed by 1:1 serial dilutions in 1× Binding Buffer. RNA and compound solutions were then mixed 1:1 by volume to a total of 20 µL.

Samples were incubated for 20 min at room temperature and then loaded into premium capillaries (NanoTemper Tech, Cat# MO-K025). The following parameters were used for MST measurements: 5 – 20% LED power (adjusted to keep fluorescence intensity between 2000 and 8000), 80% MST power, Laser-On time = 30 s, Laser-Off time = 25 s. The resulting data were analyzed by calculating the change in thermophoresis as a function of compound concentration and fitted by Equation 1, a one site binding model, in NanoTemper Tech's MST analysis software to yield the dissociation constant (K_d):

$$f(c) = unbound + \frac{bound - unbound}{2} * (F + c + K_d - \sqrt{((F + c + K_d)^2 - 4 * F * c)}) \quad \text{(Eq 1)}$$

Where F is the concentration of fluorescently labeled RNA; unbound and bound refer to the thermophoresis signal at completely unbound and bound state of RNA, respectively; c is the concentration of the compound; $f(c)$ is the thermophoresis signal at compound concentration of c; and K_d is the dissociation constant.

For competitive binding assays, both WT and mutant pri-miR-27a RNAs were transcribed as previously described (see **Table S6** for sequences).(11) The RNA was purified by gel electrophoresis and folded in 1× Binding Buffer at final concentration of 750 nM by heating at 60 °C for 5 min and then slowly cooling to room temperature. This RNA was aliquoted (10 µL) and diluted with an equal volume of 1× Binding Buffer. To each pri-miR-27a sample was added 10 µL of folded Cy5-labeled 5'G<u>A</u>G/3'C<u>C</u>C loop displayed in pri-miR-27a's Drosha site (folded as described above; 5 nM final concentration). To each tube was then added 10 µL of 750 nM of **9** (final concentration of 100 nM), prepared in1× Binding Buffer containing 0.05% (v/v) Tween-20. The samples were incubated at room temperature for 20 min, followed by thermophoresis

analysis as described above. The resulting data were analyzed by SigmaPlot and fitted by Equation 2 to yield the competitive dissociation constant ($K_{d\_competitive}$).

$$f(c) = a * \left(\frac{1}{2[F]}\right) * (K_c + \left(\frac{K_c}{K_d}\right) * c + 2[F] - (K_c + \left(\frac{K_c}{K_d}\right) * c + 2[F])^2 - 4[F]^2)^{0.5} + A \quad \text{(eq 2)}$$

where F is the concentration of fluorescence-labeled RNA; unbound and bound refers to the thermophoresis signal at completely unbound and bound state of RNA, respectively; c is the concentration of the compound; f(c) is the thermophoresis signal at compound concentration of c; K_d is the dissociation constant. K_c is the competitive dissociation constant.

CELLULAR METHODS

Cell lines. Compounds were tested in MDA-MB-231 TNBC cells (HTB-26, ATCC), LNCaP metastatic prostate cancer cells (gifted from Junli Luo lab), MCF-7 (HTB-22, ATCC) breast cancer cells, HeLa cervical adenocarcinoma cells (CCL-2, ATCC), and MCF-10a, a model of healthy breast epithelial cells (CRL- 10317, ATCC) .

Cell culture and compound treatment. All cells were maintained at 37 °C with 5% CO_2. MDA-MB-231 and LNCaP cells were cultured in RPMI 1640 medium with L-glutamine & 25 mM HEPES (Corning) supplemented with 10% (v/v) fetal bovine serum (FBS; Sigma) and 1× (v/v) Antibiotic-Antimycotic solution (Corning). MCF-7 and HeLa cells were cultured in DMEM medium with 4.5 g/L glucose (Corning), supplemented with 10% FBS, 1× (v/v) Glutagro (Corning), and 1× (v/v) Antibiotic-Antimycotic solution. MCF10a cells were cultured in DMEM/F12 50/50 with L-glutamine & 15 mM HEPES (Corning), supplemented with 10% FBS, 20 ng/mL human epidermal growth factor (Pepro Tech Inc.), 0.5 mg/mL hydrocortisone (Pfaltz & Bauer), 100 ng/mL cholera toxin (Sigma-Alrich), 10 µg/mL insulin (Sigma-Aldrich), and 1× (v/v) Antibiotic-Antimycotic solution.

MCF-10a cells were transfected with plasmids to express pri-miR-27a and mutant pri-miR-27a in 12- or 24-well plates with Lipofectamine 2000 per the manufacturer's protocol. For treatment of compounds, stocks were diluted in growth medium and added to cells for 48 h. The miRCURY miR-27a LNA inhibitor (QIAGEN, Cat# 339121) was used as a positive control by diluting in growth medium to a final concentration of 1 nM. The miRCURY LNA Negative Control (QIAGEN, Cat# YI00199007-DDA) was used as a negative control, which has also diluted in growth medium to a final concentration of 1 nM.

The plasmids encoding pri-miR-27a and mutant pri-miR-27a were custom synthesized by GenScript. Wild type miR-27a hairpin plasmid (Cat# SC1692) was produced via VectorArk Vector

MR04 with cloning direction consistent to promoter. Mutant miR-27 hairpin plasmid (Cat# SC1441) was generated by mutagenesis on the wild type plasmid.

Analysis of mRNA abundance: Each cell line was grown as a monolayer in 12- or 24-well plates and treated as described in "**Cell culture and compound treatment**". After 48 h, the cells were lysed, and total RNA was harvested using a Zymo Quick RNA Miniprep Kit per the manufacturer's protocol. To measure the abundance of mature miRNAs, approximately 250 ng of total RNA was reverse transcribed using the High Flex Buffer provided in a miScript II RT Kit (Qiagen) per the manufacturer's protocol (10 µL total reaction volume). For pri-miRNAs and mRNA, approximately 300 ng of total RNA was reverse transcribed using a qScript cDNA synthesis kit (10 µL total reaction volume, Quanta BioSciences). For all types of RNAs, 2 µL of the RT reaction was used for qPCR using SYBR Green Master Mix and a QuantStudio™ 5 Real-Time PCR System. Relative abundance of mature miRNAs was calculated by normalizing to *RNU6*, and relative abundance for pri-miRNAs and mRNAs was calculated by normalizing to *18S* ribosomal RNA, using the $\Delta\Delta C_t$ method.(12)

Western Blotting: MDA-MB-231 cells were grown in 6-well plates to ~80% confluency in complete growth medium and then incubated with **9** at the indicated concentrations for 48 h. Total protein was extracted using M-PER Mammalian Protein Extraction Reagent (Pierce Biotechnology) using the manufacturer's protocol and quantified using a Micro BCA Protein Assay Kit (Pierce Biotechnology). Approximately 50 µg of total protein was separated on a 10% SDS-polyacrylamide gel, and then transferred to a PVDF membrane. The membrane was washed with 1× Tris-buffered saline (TBS) and then blocked in 1× TBST (1× TBS containing 0.1% (v/v) Tween-20) containing 5% (w/v) milk for 1 h at room temperature. After washing with 1× TBST, the membrane was incubated with a 1:1000 dilution of rabbit anti-PDCD4 (Cell Signaling Technology:

D29C6), a 1:2000 dilution of rabbit anti-ZBTB10 (catalog number: ab117786; Abcam), or a 1:2000 of rabbit anti-PP4C (catalog number: ab227267; Abcam) in 1× TBST containing 5% milk overnight at 4 °C. The membrane was then washed with 1× TBST and incubated with 1:5000 anti-rabbit IgG horseradish-peroxidase secondary antibody conjugate (catalog number: 7076; Cell Signaling Technology) in 1× TBS for 2 h at room temperature. The membrane was again washed with 1× TBST, and protein expression was quantified using SuperSignal West Pico Chemiluminescent Substrate (Pierce Biotechnology) per the manufacturer's protocol.

To quantify β-actin expression, used for normalization, the membrane was stripped using 1× Stripping Buffer (200 mM glycine, pH 2.2 and 0.1% SDS) followed by washing in 1× TBST. The membrane was blocked and probed for β-actin as described above using a 1:5000 dilution of mouse anti-β-actin antibody (8H10D10; catalog number: 3700S; Cell Signaling Technology) at room temperature for 2 h and 1:5000 anti-mouse IgG horseradish-peroxidase secondary antibody conjugate (catalog number: 7074; Cell Signaling Technology) in 1× TBS for 2 h at room temperature. The fold change of the target protein expression (PP4C or PDCD4) was calculated by normalizing its band intensity to β-actin band intensity using ImageJ.

Migration assay: MDA-MB-231 cells were grown in 60 mm diameter dishes and treated as described in "**Cell culture and compound treatment**" for 12 h. The medium was then removed and replaced with growth medium lacking FBS but with the same concentration of compound. After 12 h of serum starvation, the cells were detached and seeded to ThinCert™ (GBO) 24-well hanging inserts with 8 µm pores (~5 × 10^4 cells per insert). Fresh growth medium with FBS was dispended in a 24-well plate (600 µL each well), and the ThinCert™ hanging inserts containing cells were then placed into the wells. After incubating for 24 h, the growth medium in the inserts was removed, and the remaining cells were washed twice with 1× DPBS. Cells were fixed by addition of 3% (w/v) paraformaldehyde (PFA) prepared in 1× DPBS at room temperature for 20

min. The PFA solution was removed, and cells were washed twice with 1× PBS. The cells were then stained with Crystal Violet (10 mg/mL; 4:1 H_2O:MeOH) at room temperature for 20 min. A cotton swab was used to gently remove non-migrating cells from the top side of the filter. The remaining cells were then imaged by microscopy and counted for quantification (3 fields of view per sample).

PROTEOMICS

Global proteomics profiling using LC-MS/MS: Treated cell pellets were resuspended in 1× PBS, lysed by sonication, and protein concentration was determined by using a Bradford assay (Bio-Rad). Samples (30 µg) were denatured in 6 M urea in 50 mM NH_4HCO_3, pH 8, reduced for 30 min with 10 mM TCEP, and alkylated for 30 min in the dark with 25 mM iodoacetamide. Samples were diluted to 2 M urea with 50 mM NH_4HCO_3, pH 8, and the proteins were digested with trypsin (1 µL of 0.5 µg/µL) in the presence of 1 mM $CaCl_2$ for 12 h at 37 °C. The samples were acidified by adding acetic acid to a final concentration of 5% (v/v). After desalting the samples over a self-packed C18 spin column, they were dried and analyzed by LC-MS/MS (see below). The resulting MS data were processed with MaxQuant as described below.

LC-MS/MS analysis: Peptides were dissolved in water containing 0.1% formic acid (FA) and analyzed using an EASY-nLC 1200 nano-UHPLC connected to a Q Exactive HF-X Quadrupole-Orbitrap mass spectrometer (Thermo Scientific). A 50 cm long, 75 µm i.d. chromatography column packed with ReproSil-Pur 120 C18-AQ 2.4 µm beads (Dr. Maisch GmbH) and capped by a 5 µm tip was used. Water with 0.1% FA in water (Buffer A) and 90% acetonitrile (MeCN):10% water with 0.1% FA (Buffer B) were used as liquid chromatography solvents. A flow rate of 300 nL/min over a 240 min linear gradient (5-35% Buffer B) at 65 °C was used to elute peptides

into the mass spectrometer. Data-dependent acquisition (top-20, NCE 28, R = 7,500) after full MS scan (R = 60,000, m/z 400-1,300) with a dynamic exclusion of 10 seconds was performed. Peptide match to prefer and isotope exclusion were selected and enabled.

MaxQuant analysis: The MaxQuant software(13) (V1.6.1.0) was used to analyzed the MS data and searched against the human proteome (Uniprot) and a common list of contaminants (included in MaxQuant). Peptide search tolerance was set to 20 ppm for the first search and 10 ppm for the main search while 0.02 Da was used for the fragment mass tolerance. The false discovery rate (FDR) for peptides, proteins and sites identification was set to 1%. Peptide length was set to at least 6 amino acids and peptide re-quantification was enabled. Label-free quantification (MaxLFQ) and "match between runs" were activated. Number of peptides per protein was set to ≥ 2. Searched modifications were methionine oxidation (variable modification) and carbamidomethylation of cysteines (fixed modification).

TargetScan analysis: TargetScanHuman v7.2 was used to predict downstream protein targets of miR-27a-3p (n = 1421), miR-23a-3p (n = 1342) and miR-24-3p (n = 761) containing conserved sites, all proteins with a context score ≤0 were included to the analysis per TargetScan recommendation. Approximately 15% of miR-27a-3p targets (220/1421), ~18% of miR-23a-3p targets (247/1342), and ~16% of miR-24-3p targets (122/761) were detectable in the global proteomics analysis. Cumulative distribution plots of the fold change of proteins in **9**-treated vs. vehicle-treated samples indicated a significant upregulation of only miR-27a-3p targets (red), while no significant change was observed with miR-23a-3p targets (green) and miR-24-3p (blue), relative to the cumulative distribution of all proteins (black) (**Figure 4B**). Targets of miR-23a-3p and miR-24-3p were used as comparison as the two miRNAs are expressed at similar levels as

miR-27a-3p in MDA-MB-231 cells and are located in the same cluster. *P* values between distributions were calculated using a two-tailed Kolmogorov-Smirnov test.

COMPOUND CHARACTERIZATION

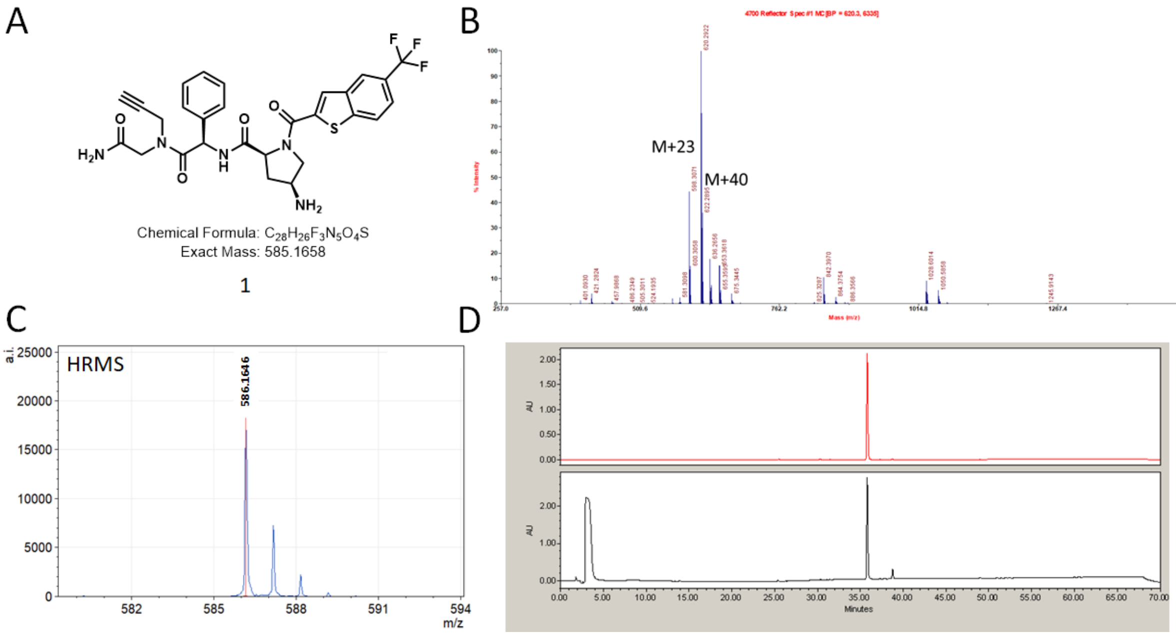

Figure S22. Characterization of compound 1. (A) Chemical structure of the compound. (B) full-length MALDI-MS spectrum. (C) zoom in on the internally calibrated HRMS spectrum. (D) HPLC traces at 220 nm (top) and 254 nm (bottom).

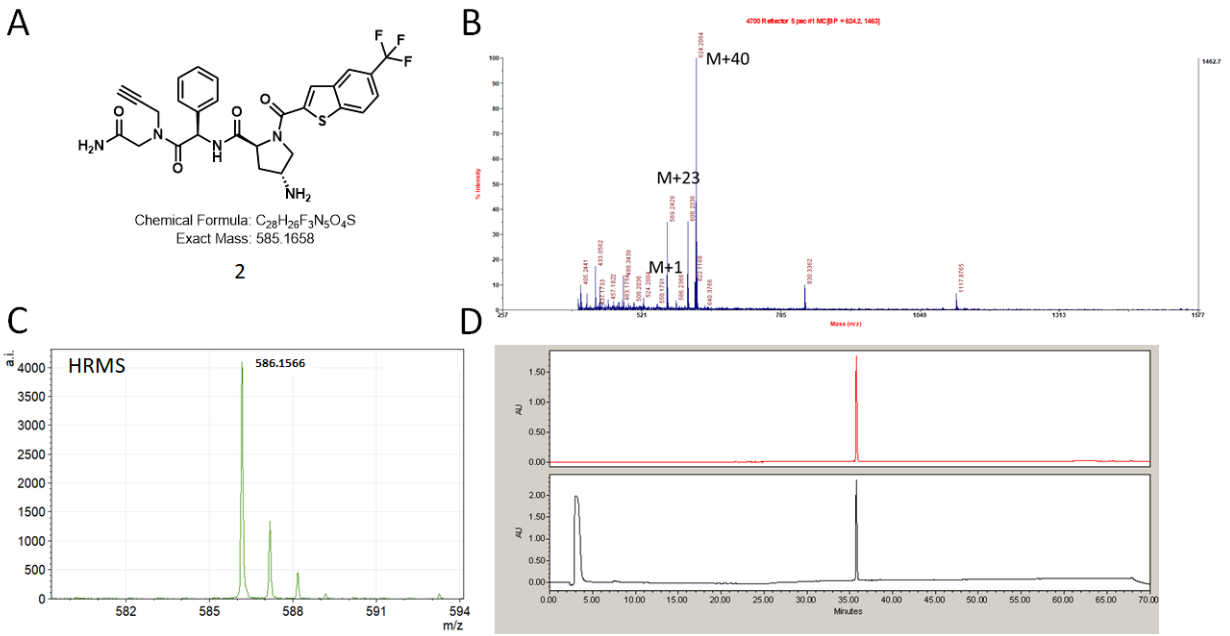

Figure S23. Characterization of compound 2. (A) Chemical structure of the compound. (B) full-length MALDI-MS spectrum. (C) zoom in on the internally calibrated HRMS spectrum. (D) HPLC traces at 220 nm (top) and 254 nm (bottom).

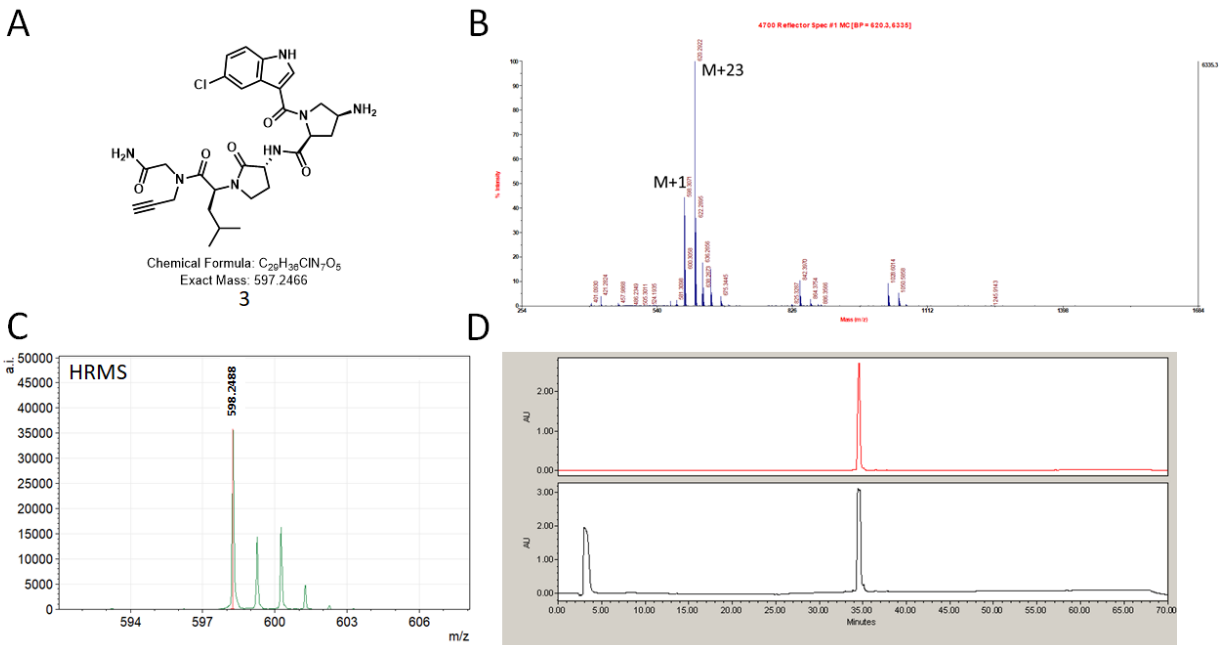

Figure S24. Characterization of compound 3. **(**A) Chemical structure of the compound. (B) full-length MALDI-MS spectrum. (C) zoom in on the internally calibrated HRMS spectrum. (D) HPLC traces at 220 nm (top) and 254 nm (bottom).

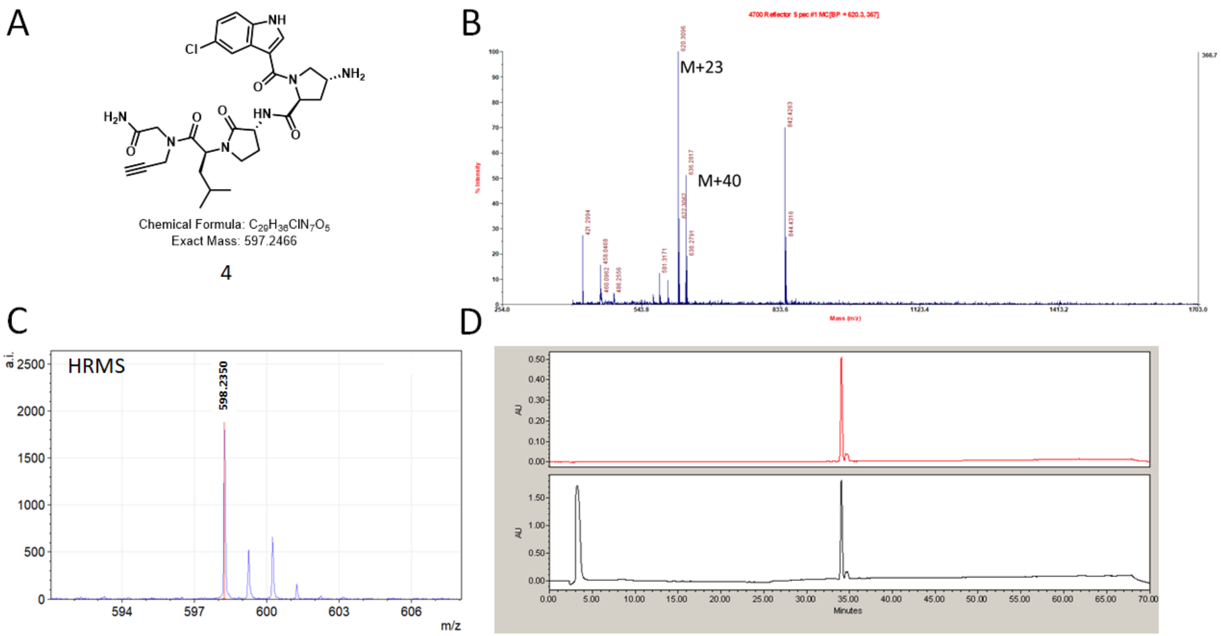

Figure S25. Characterization of compound 4. (A) Chemical structure of the compound. (B) full-length MALDI-MS spectrum. (C) zoom in on the internally calibrated HRMS spectrum. (D) HPLC traces at 220 nm (top) and 254 nm (bottom).

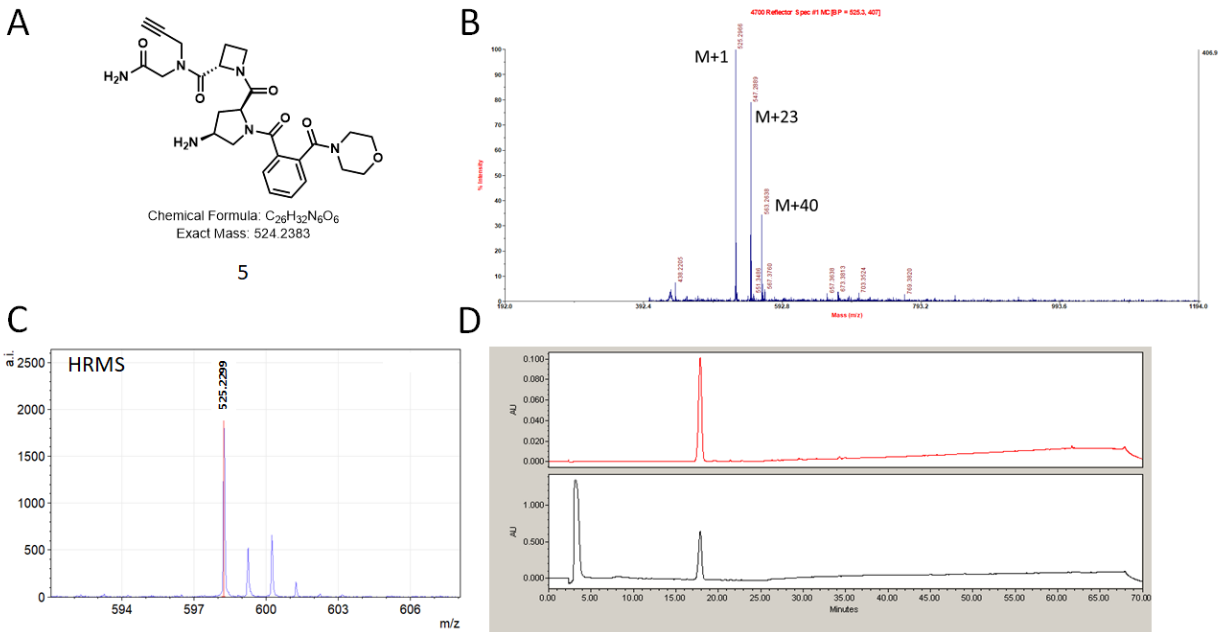

Figure S26. Characterization of compound 5. (A) Chemical structure of the compound. (B) full-length MALDI-MS spectrum. (C) zoom in on the internally calibrated HRMS spectrum. (D) HPLC traces at 220 nm (top) and 254 nm (bottom).

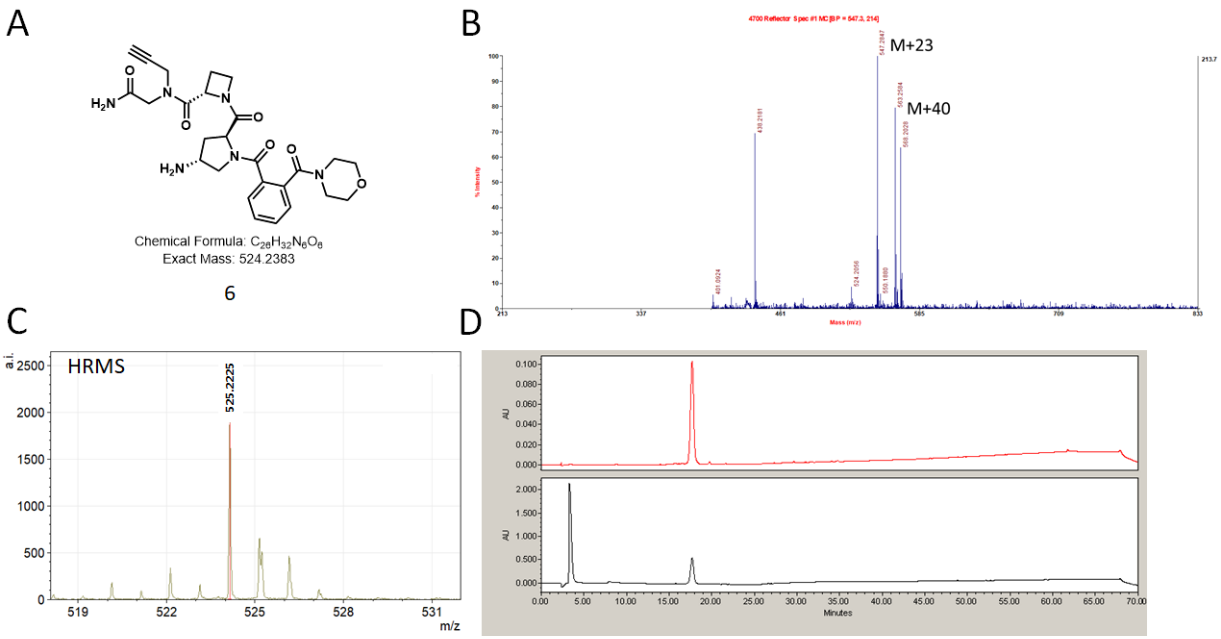

Figure S27. Characterization of compound 6. (A) Chemical structure of the compound. (B) full-length MALDI-MS spectrum. (C) zoom in on the internally calibrated HRMS spectrum. (D) HPLC traces at 220 nm (top) and 254 nm (bottom).

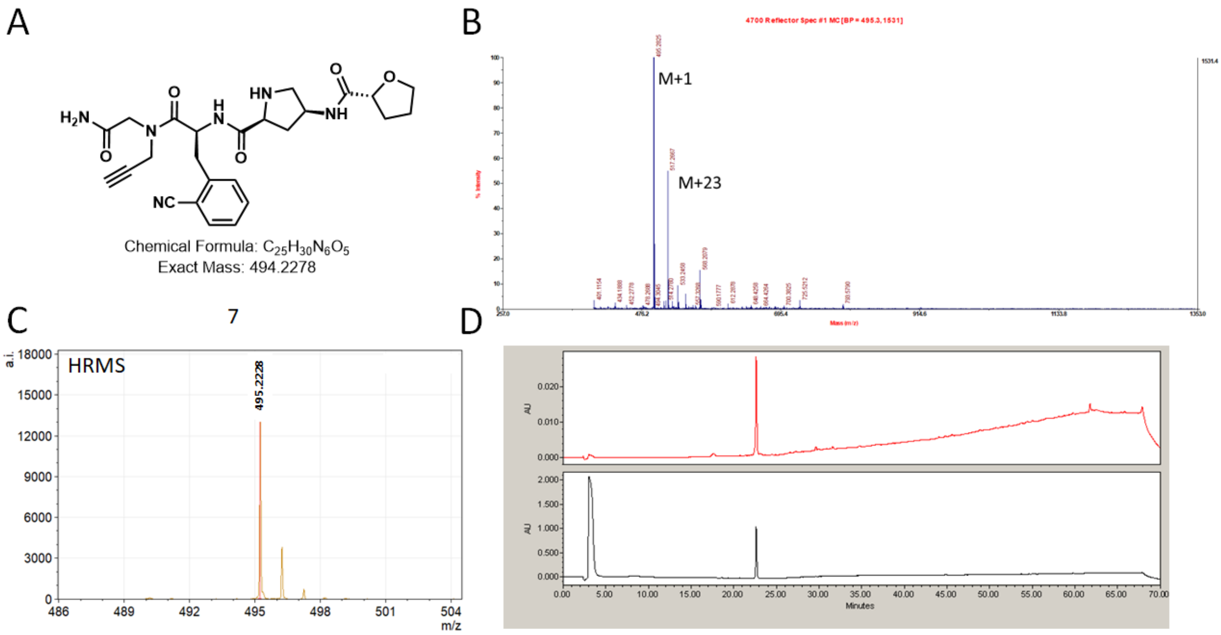

Figure S28. Characterization of compound 7. (A) Chemical structure of the compound. (B) full-length MALDI-MS spectrum. (C) zoom in on the internally calibrated HRMS spectrum. (D) HPLC traces at 220 nm (top) and 254 nm (bottom).

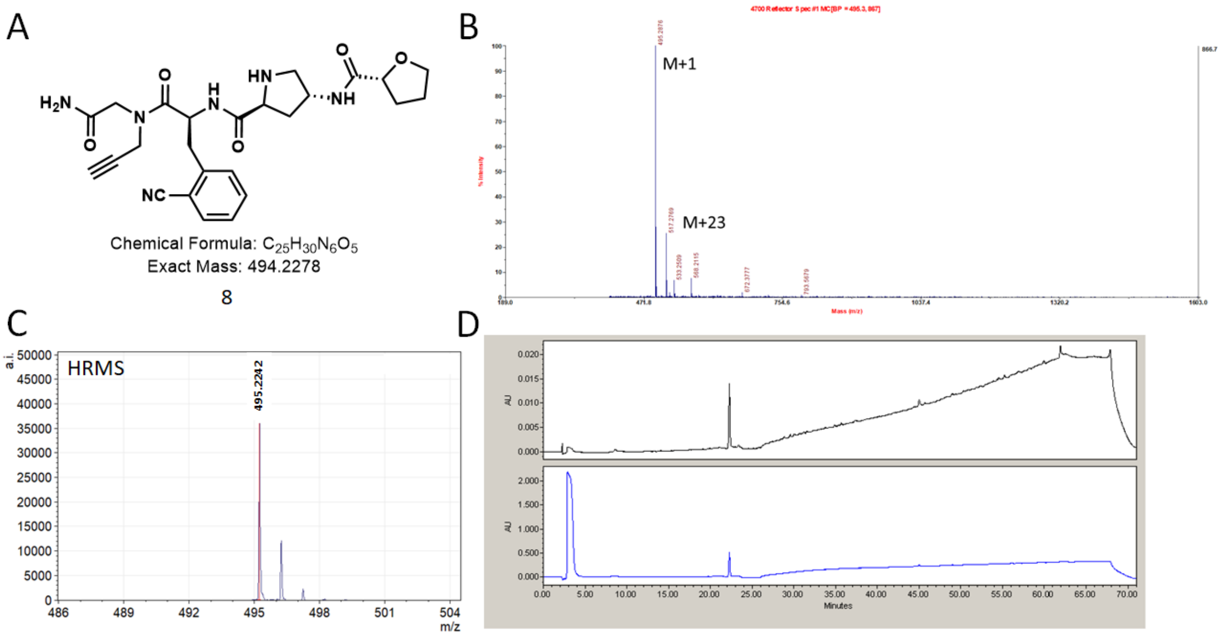

Figure S29. Characterization of compound 8. **(**A) Chemical structure of the compound. (B) full-length MALDI-MS spectrum. (C) zoom in on the internally calibrated HRMS spectrum. (D) HPLC traces at 220 nm (top) and 254 nm (bottom).

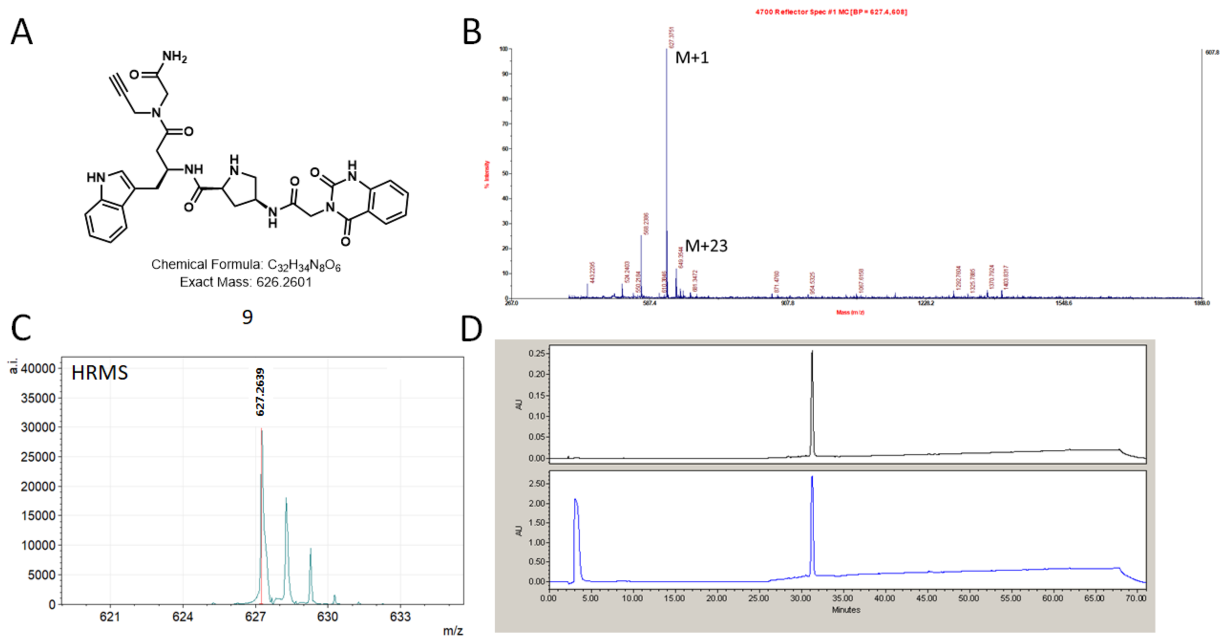

Figure S30. Characterization of compound 9. **(**A) Chemical structure of the compound. (B) full-length MALDI-MS spectrum. (C) zoom in on the internally calibrated HRMS spectrum. (D) HPLC traces at 220 nm (top) and 254 nm (bottom).

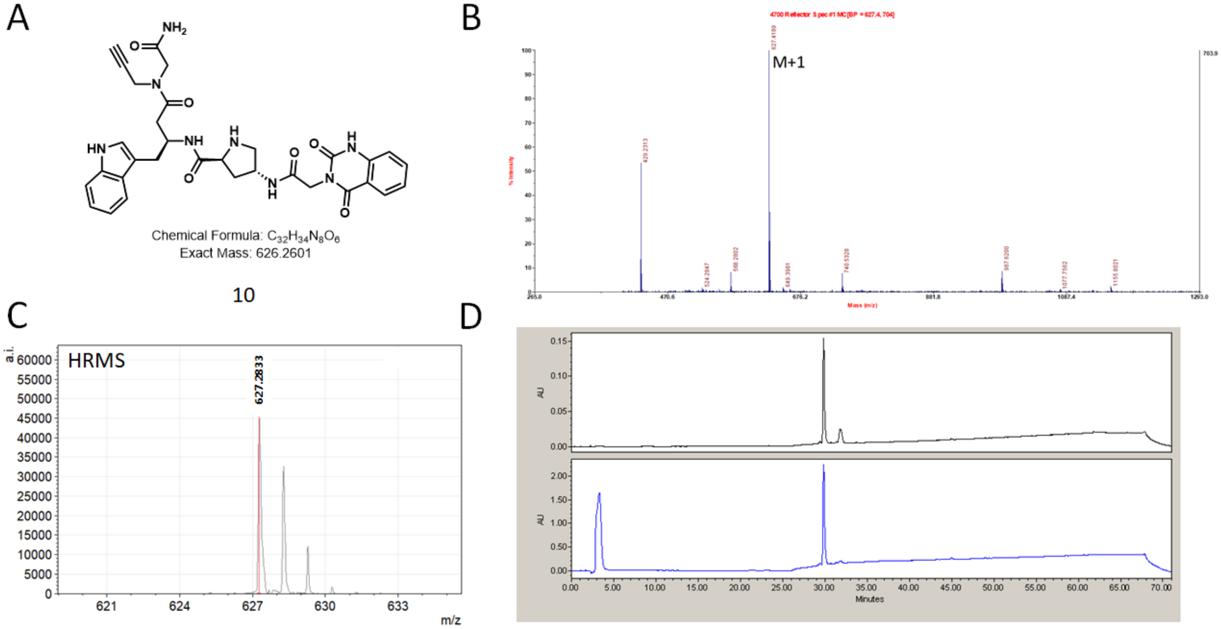

Figure S31. Characterization of compound 10. (A) Chemical structure of the compound. (B) full-length MALDI-MS spectrum. (C) zoom in on the internally calibrated HRMS spectrum. (D) HPLC traces at 220 nm (top) and 254 nm (bottom).

REFERENCES

1. M. D. Disney *et al.*, Inforna 2.0: A platform for the sequence-based design of small molecules targeting structured RNAs. *ACS Chem. Biol.* **11**, 1720-1728 (2016).
2. T. D. Schneider, R. M. Stephens, Sequence logos: a new way to display consensus sequences. *Nucleic Acids Res.* **18**, 6097-6100 (1990).
3. M. Nettling *et al.*, DiffLogo: a comparative visualization of sequence motifs. *BMC Bioinformatics* **16**, 387 (2015).
4. V. Agarwal, G. W. Bell, J. W. Nam, D. P. Bartel, Predicting effective microRNA target sites in mammalian mRNAs. *eLife* **4** (2015).
5. A. B. MacConnell, P. J. McEnaney, V. J. Cavett, B. M. Paegel, DNA-encoded solid-phase synthesis: encoding language design and complex oligomer library synthesis. *ACS Comb. Sci.* **17**, 518-534 (2015).
6. W. G. Cochrane *et al.*, Activity-based DNA-encoded library screening. *ACS Comb. Sci.* **21**, 425-435 (2019).
7. K. R. Mendes *et al.*, High-throughput identification of DNA-encoded IgG ligands that distinguish active and latent *Mycobacterium tuberculosis* infections. *ACS Chem. Biol.* **12**, 234-243 (2017).
8. T. Sander, J. Freyss, M. von Korff, C. Rufener, DataWarrior: An open-source program for chemistry aware data visualization and analysis. *J. Chem. Inf. Model.* **55**, 460-473 (2015).
9. T. Tran, M. D. Disney, Two-dimensional combinatorial screening of a bacterial rRNA A-site-like motif library: defining privileged asymmetric internal loops that bind aminoglycosides. *Biochemistry* **49**, 1833-1842 (2010).
10. S. P. Velagapudi *et al.*, Defining RNA-small molecule affinity landscapes enables design of a small molecule inhibitor of an oncogenic noncoding RNA. *ACS Cent. Sci.* **3**, 205-216 (2017).
11. M. G. Costales, Y. Matsumoto, S. P. Velagapudi, M. D. Disney, Small molecule targeted recruitment of a nuclease to RNA. *J. Am. Chem. Soc.* **140**, 6741-6744 (2018).
12. K. J. Livak, T. D. Schmittgen, Analysis of relative gene expression data using real-time quantitative PCR and the 2(-Delta Delta C(T)) Method. *Methods (San Diego, Calif.)* **25**, 402-408 (2001).
